# Supplementary material for: Cross-sectional health centre and community-based evaluation of the impact of pneumococcal and malaria vaccination on antibiotic prescription and usage, febrile illness and antimicrobial resistance in young children in Malawi: the IVAR study protocol
Source: BMJ Open. 2023 May 12;13(5):e069560. doi: 10.1136/bmjopen-2022-069560 (PMC10186476; doi:10.1136/bmjopen-2022-069560)
Supplement: Supplementary data [file bmjopen-2022-069560supp001.pdf]

**Supplementary file 1:** # Case Report Form – Health Center Audit

**Questionnaire ID.**
Label

## SCREENING

| | | |
|---|---|---|
| 1 | Today's date (dd-mmm-yyyy) | \|___\|___\| - \|___\|___\|___\| - \| 2 \| 0 \|___\|___\| |
| 2 | Health Center name | |

*Enumerator: The following questions should be answered using the child's Health Passport. If not available, the child is **ineligible** for study participation.*

**Inclusion Criteria**

| | | |
|---|---|---|
| 3 | Is the child's health passport available for review? | **No Yes UNK** |
| 4 | Is the child aged 3 years of age or younger? | **No Yes UNK** |
| 5 | Has the child presented today for investigation and/or treatment of ill health? | **No Yes UNK** |

**Exclusion Criteria**

| | | |
|---|---|---|
| 6 | Has the child presented today for a routine health check? | **No Yes UNK** |
| 7 | Has the child presented today for a vaccination? | **No Yes UNK** |

**Health Passport**

| | | |
|---|---|---|
| 8 | ***Enumerator***<br>*Is the child eligible, including:*<br>• *Health passport **available** for review*<br>• *Aged **3 years of age or under***<br>• *Presenting at the health center for investigation and/or treatment of ill health*<br>***Note:*** *If no, stop review and explain why not eligible.* | **No Yes** |

## CONSENT

| | | |
|---|---|---|
| 9 | *Has the carer had the opportunity to read (or had read to them) the study information sheet?* | **No Yes** |
| 10 | *Has the carer had the opportunity to ask questions about this study?* | **No Yes** |
| 11 | *Has verbal consent been obtained from carer?* | **No Yes** |

## HEALTH PASSPORT

**Vaccine status**

| | | |
|---|---|---|
| 12 | Has the child received at least the **initial course (3 doses)** of the **RTS,S/AS01 malaria** vaccination? | **No Yes Not yet eligible UNK** |
| 13 | Has the child received the **RTS,S/AS01 malaria booster** vaccination? | **No Yes Not yet eligible UNK** |
| 14 | Which **PCV13** vaccination schedule is the child in? | **3+0 2+1 UNK** |
| 15 | Has the child received all **PCV13** vaccinations that they are currently eligible for (according to their age)? | **No Yes UNK** |

## TODAY'S VISIT

The following questions ask about the child's visit to the health center today.

**Malaria rapid diagnostic test use:**

| | | |
|---|---|---|
| 20 | Was a **malaria rapid diagnostic test (RDT)** performed **today**? | **No Yes UNK** |

**Medicinal prescription:**

| | | |
|---|---|---|
| 21 | Is there any recorded **medicinal** prescription in **today's** visit? | **No Yes UNK** |
| | *If yes, please record medicine(s) prescribed:* | |



Singleton D, *et al. BMJ Open* 2023; 13:e069560. doi: 10.1136/bmjopen-2022-069560

**Supplementary file 1:** **Case Report Form – Health Center Audit**

| Medicine prescribed | Route of administration | Course length (days) |
|---|---|---|
| | | |
| | | |
| | | |
| | | |

| | | | |
|---|---|---|---|
| 22 | *Form completed by (Enumerator Code):* | *Code* | /___/___/___/ |
| 23 | *Form completed by:* | *Signature* | |



Singleton D, *et al. BMJ Open* 2023; 13:e069560. doi: 10.1136/bmjopen-2022-069560
