# Supplementary material for: Cross-sectional health centre and community-based evaluation of the impact of pneumococcal and malaria vaccination on antibiotic prescription and usage, febrile illness and antimicrobial resistance in young children in Malawi: the IVAR study protocol
Source: BMJ Open. 2023 May 12;13(5):e069560. doi: 10.1136/bmjopen-2022-069560 (PMC10186476; doi:10.1136/bmjopen-2022-069560)
Supplement: Supplementary data [file bmjopen-2022-069560supp004.pdf]

Supplementary file 4:

Case Report Form – Mangochi Survey

**Study ID No.** Label	**Lab ID No.** Label

SCREENING

	Today's date (dd-mmm-yyyy)	|___|___| - |___|___|___| - | 2 | 0 |___|___|
1	What is your child's date of birth? (dd-mmm-yyyy)	|___|___| - |___|___|___| - | 2 | 0 |___|___|
1a	What is your child's age?<br>*(Note to enumerator: If date of birth unknown)*	
2	*Enumerator: Is this a cluster where the RST,S/AS01 vaccine is implemented?*	**No Yes UNK**

Inclusion Criteria

3	Has your child received a full initial course of the RST,S/AS01 vaccine?	**No Yes UNK**
4	Is your child healthy?	**No Yes UNK**

Exclusion Criteria

5	Has your child received (any) antibiotics within the previous 14 days?	**No Yes UNK**
6	Is your child currently on TB treatment?	**No Yes UNK**
7	Has your child been hospitalized for pneumonia within the previous 14 days?	**No Yes UNK**
8	Does your child have a (gross) respiratory tract pathology?	**No Yes UNK**
9	Does your child have a terminal illness?	**No Yes UNK**
10	Has your child been previously recruited into this study during this survey?	**No Yes UNK**

Health Passport

11	***Enumerator***<br>*Is the child eligible, including:*<br>• *Aged **18-36 months***<br>• *Permanent resident in Mangochi District*<br>• *No antibiotic use/pneumonia in last 14 days*<br>• *Not currently on TB treatment*<br>• *For children in the RTS,S/AS01 vaccine cluster, they **MUST** have received the full initial vaccination course, but are **NOT** required to have received the booster vaccination*<br>***Note:*** *If no, stop interview and explain why not eligible.*	**No Yes**

RECRUITMENT - PRELIMINARY DATA

12	*Was consent obtained from carer?*	**No Yes**
13	*If yes, scan the barcode for Participant ID*	
14	*If scanner not available, write the Participant ID*	**EVAL - 1 - __ __**
15	Has this child been recruited during a previous survey?<br>*(Enumerator: A participant cannot be recruited into study during same survey)*	**No Yes UNK**

Singleton D, *et al. BMJ Open* 2023; 13:e069560. doi: 10.1136/bmjopen-2022-069560

Supplementary file 4: **Case Report Form – Mangochi Survey**

RECRUITMENT - METADATA

Child Characteristics

16	What is the sex of this child?	Male Female
17	Has the child ever tested positive for HIV?	No Yes UNK

Mother's HIV status (if mother is the carer consenting)

18	Have you ever tested positive for HIV?<br>*Enumerator: Only ask this question if interviewing the mother.*	No Yes UNK N/A
19	Was your HIV-infection confirmed before the recruited child was born?<br>*Enumerator: Only ask this question if date not known.*	No Yes UNK

The following questions are about vaccines your child may have received as part of the routine EPI.

20	Do you have the child's Health Passport with you?	No Yes
21	*Enumerator: Are you able to confirm RTS,S/AS01 vaccination dates by Health Passport?*	*No Yes*
22	*Enumerator: If yes, take a photo of the vaccination page of Health passport*	*Not Done Done*

Vaccine status

	Vaccine	*Vaccines received* (*Circle answer*)		*Date of Vaccination* (*dd-mmm-yyyy*)
	Birth / first contact			
23a	*BCG*	***No Yes UNK***	23b	|___|___| - |___|___|___| - |___|___|___|___|
24a	*OPV 0*	***No Yes UNK***	24b	|___|___| - |___|___|___| - |___|___|___|___|
	6 weeks of age			
25a	*OPV 1*	***No Yes UNK***	25b	|___|___| - |___|___|___| - |___|___|___|___|
26a	*Rota1*	***No Yes UNK***	26b	|___|___| - |___|___|___| - |___|___|___|___|
27a	*DPT-HepB-Hib1*	***No Yes UNK***	27b	|___|___| - |___|___|___| - |___|___|___|___|
28a	*PCV1*	***No Yes UNK***	28b	|___|___| - |___|___|___| - |___|___|___|___|
	10 weeks of age			
29a	*OPV22*	***No Yes UNK***	29b	|___|___| - |___|___|___| - |___|___|___|___|
30a	*Rota2*	***No Yes UNK***	30b	|___|___| - |___|___|___| - |___|___|___|___|
31a	*DPT-HepB-Hib2*	***No Yes UNK***	31b	|___|___| - |___|___|___| - |___|___|___|___|
32a	*PCV2*	***No Yes UNK***	32b	|___|___| - |___|___|___| - |___|___|___|___|
	14 weeks of age			
33a	*OPV33*	***No Yes UNK***	33b	|___|___| - |___|___|___| - |___|___|___|___|
34a	*DPT-HepB-Hib3*	***No Yes UNK***	34b	|___|___| - |___|___|___| - |___|___|___|___|
35a	*PCV3*	***No Yes UNK***	35b	|___|___| - |___|___|___| - |___|___|___|___|
36a	*IPV*	***No Yes UNK***	36b	|___|___| - |___|___|___| - |___|___|___|___|
	5-7 months of age			

Singleton D, *et al. BMJ Open* 2023; 13:e069560. doi: 10.1136/bmjopen-2022-069560

Supplementary file 4: **Case Report Form – Mangochi Survey**

37a	Malaria RTS,S/AS01 1	No Yes UNK	37b	|___|___| - |___|___|___|___| - |___|___|___|___|
38a	Malaria RTS,S/AS01 2	No Yes UNK	38b	|___|___| - |___|___|___|___| - |___|___|___|___|
39a	Malaria RTS,S/AS01 3	No Yes UNK	39b	|___|___| - |___|___|___|___| - |___|___|___|___|
	9-11 months of age			
40a	Measles-Rubella1	No Yes UNK	40b	|___|___| - |___|___|___|___| - |___|___|___|___|
	15-23 months of age			
42a	Measles-Rubella2	No Yes UNK	42b	|___|___| - |___|___|___|___| - |___|___|___|___|
44a	Malaria RTS,S/AS01 4	No Yes UNK	44b	|___|___| - |___|___|___|___| - |___|___|___|___|

Household information

The following questions will be about the house the child lives in, including who lives in the home and its location

45	GPS coordinates	lat |__|__|__|.|__|__|__|__|__| / long |__|__|__|__|.|__|__|__|__|__|
45a	Enumerator: If no GPS coordinates available, record why not available.	

46	How many bedrooms does the child's main house have?	
47	How many adults (16+ years of age) live in the main house?	
48	How many children 5-15 years of age live in the main house, including child recruited today?	
49	How many children 0-4 years of age live in the main house?	

Smoking

50	Does anybody in the child's household smoke tobacco (cigarettes, pipes, or cigars)?	**No Yes**

The following questions ask about the type of house the child lives in.

51 **What type of exterior wall does the house have?**

1 Burnt brick
2 Unburnt brick
3 Pounded thick mud
4 Plastered thin mud
5 Bamboo
6 Grass or no walls
7 Iron sheets
8 Concrete blocks
99 Other, specify: __________________________

52 **What type of roof does the house have?**

1 Grass or leaves
2 Grass+Iron sheets
3 Grass+plastic sheet
4 Iron sheets or tiles

53 **What is the condition of the roof?**

1 Good
2 Poor (leaks water)

54 **What type of floor does the house have inside?**

1 Mud
2 Concrete/ cement
3 Tiles
99 other (specify): ____

55 **What type of toilet does the house have?**

1 Simple pit latrine
2 VIP
3 Water toilet
3 None (including use the neighbour's toilet)

56 **What source of electricity does the house have?**

1 Escom
2 Solar
3 None

57 **What source of drinking water does the house have?**

Singleton D, *et al. BMJ Open* 2023; 13:e069560. doi: 10.1136/bmjopen-2022-069560

Supplementary file 4: **Case Report Form – Mangochi Survey**

	1 Tap to house	3 Bore hole	5 Open well
	2 Shared communal tap	4 Covered well	6 River
58	**Does the house have glass windows?**		
	0 No	1 Yes	

Possessions

The following questions ask about some possessions you may have. We are not able to give you any of these items, even if you report not having them.

59	Are you comfortable answering questions about items owned by people in your household?	**No Yes**

Does anyone in the household possess any of the following **working** items?

60	Watch or clock	**No Yes**	71	Bed	**No Yes**
61	Radio	**No Yes**	72	Upholstered chair/sofa	**No Yes**
62	Bank account (or bank book)	**No Yes**	73	Table	**No Yes**
63	Charcoal iron	**No Yes**	74	Bicycle	**No Yes**
64	Sewing machine	**No Yes**	75	Motorbike	**No Yes**
65	Mobile phone	**No Yes**	76	Car	**No Yes**
66	Tape/CD player	**No Yes**	77	Television	**No Yes**
67	Fan, electric	**No Yes**	78	Refrigerator	**No Yes**
68	Mosquito net	**No Yes**	79	Other electric items	**No Yes**
69	Number of mosquito nets		80	If other **working** electrical items, specify:	
70	Mattress	**No Yes**			

Education

The following questions ask about the head of your household's education. It maybe you, or it may be someone else

81	Are you comfortable answering questions about the head of your household's education?	**No Yes**

82	**What is the highest educational qualification the household head has acquired?**			
	1 None	3 JCE	5 Non-university diploma	7 Postgraduate degree
	2 PSLCE	4 MSCE	6 University diploma/degree	
83	**Is the household head able to read and write in English?**			
	1 No	2 Yes		

MALARIA, FEBRILE ILLNESS & MEDICINE USE

The following questions ask about your child's history of malaria and/or febrile illness, and their use of medicines.

Body temperature history and malaria rapid diagnostic test use:

84	*Enumerator: If the child's Health Passport is available, are there any occasions where their **body temperature** has been recorded?*	No Yes
85	*If yes, please record date(s) of recording(s) and temperature:*	

Date of recording (dd-mmm-yyyy)	*Temperature Recorded (°C)*
|___|___| - |___|___|___| - |___|___|___|___|	
|___|___| - |___|___|___| - |___|___|___|___|	

Singleton D, *et al. BMJ Open* 2023; 13:e069560. doi: 10.1136/bmjopen-2022-069560

Supplementary file 4: **Case Report Form – Mangochi Survey**

/___/___/ - /___/___/___/ - /___/___/___/___/	
/___/___/ - /___/___/___/ - /___/___/___/___/	
/___/___/ - /___/___/___/ - /___/___/___/___/	
/___/___/ - /___/___/___/ - /___/___/___/___/	
/___/___/ - /___/___/___/ - /___/___/___/___/	
/___/___/ - /___/___/___/ - /___/___/___/___/	

86	*Enumerator: If the child's Health Passport is available, are there any recorded usages of **malaria rapid diagnostic tests (RDT)**?*	No Yes
87	*If yes, please record date(s) of malaria rapid diagnostic test(s):*	

Date of malaria RDT (dd-mmm-yyyy)	*Result*
/___/___/ - /___/___/___/ - /___/___/___/___/	*Negative Positive UNK*
/___/___/ - /___/___/___/ - /___/___/___/___/	*Negative Positive UNK*
/___/___/ - /___/___/___/ - /___/___/___/___/	*Negative Positive UNK*
/___/___/ - /___/___/___/ - /___/___/___/___/	*Negative Positive UNK*
/___/___/ - /___/___/___/ - /___/___/___/___/	*Negative Positive UNK*
/___/___/ - /___/___/___/ - /___/___/___/___/	*Negative Positive UNK*
/___/___/ - /___/___/___/ - /___/___/___/___/	*Negative Positive UNK*
/___/___/ - /___/___/___/ - /___/___/___/___/	*Negative Positive UNK*

Enumerator: The following questions are to be directly asked to the questionnaire respondent.

88	*When did your child last suffer from a fever?*	
89	*Has your child suffered from fever in the last **14 days?***	**No Yes UNK**
89a	*If yes, how many times?*	
89b	*If yes, how many times did they need to see a doctor for a fever in the last **14 days?***	
89c	*If yes, how many times did they have to stay in hospital for fever in the last **14 days?***	
90	*Has your child suffered from fever in the last **14 days to 3 months?***	**No Yes UNK**
90a	*If yes, how many times?*	
90b	*If yes, how many times did they need to see a doctor for a fever in the last **14 days to 3 months?***	
90c	*If yes, how many times did they have to stay in hospital for fever in the last **14 days to 3 months?***	
91	*Has your child suffered from fever in the last **3 to 12 months?***	**No Yes UNK**
91a	*If yes, how many times?*	
91b	*If yes, how many times did they need to see a doctor for a fever in the last **3 to 12 months?***	
91c	*If yes, how many times did they have to stay in hospital for fever in the last **3 to 12 months?***	

Medicine use:

Singleton D, *et al. BMJ Open* 2023; 13:e069560. doi: 10.1136/bmjopen-2022-069560

Supplementary file 4: **Case Report Form – Mangochi Survey**

92	*Enumerator: If the child's Health Passport is available, are there any recorded prescription of **medicines**?*	No Yes
93	*If yes, please record date(s) of medicine prescription:*	

Date of medicine prescription (dd-mmm-yyyy)	Medicine prescribed	Diagnosis (if stated)	Route of administration	Course length (days)
|__|__| - |__|__|__| - |__|__|__|__|				
|__|__| - |__|__|__| - |__|__|__|__|				
|__|__| - |__|__|__| - |__|__|__|__|				
|__|__| - |__|__|__| - |__|__|__|__|				
|__|__| - |__|__|__| - |__|__|__|__|				
|__|__| - |__|__|__| - |__|__|__|__|				
|__|__| - |__|__|__| - |__|__|__|__|				
|__|__| - |__|__|__| - |__|__|__|__|				

94	*Other than those listed within your child's health passport, have you ever given your child any other **medicines**?*	**No Yes UNK**
94a	*If yes, what **medicines** have you given?*	
	*Enumerator: If the health passport is **NOT** available, the following questions are to be directly asked to the questionnaire respondent:*	
95	*Has your child been given **antibiotics** in the last **14 days to 3 months?***	***No Yes***
95a	*If yes, what antibiotics (active substance)?*	
95b	*If yes, how many courses (prescriptions) of antibiotics have they received in the last **14 days to 3 months?***	
95c	*Why was your child given antibiotics?*	
96	*Has your child been given **antibiotics** in the last **3 to 12 months?***	***No Yes***
96a	*If yes, what antibiotics (active substance)?*	
96b	*If yes, how many courses (prescriptions) of antibiotics have they received in the last **3 to 12 months?***	
96c	*Why was your child given antibiotics?*	

Antibiotic drug bag capture method:

Enumerator: These questions are to be asked to ALL study participants.

97 *We would now like to ask you further questions about **antibiotics**, and would like to show you some **antibiotics** that we have brought with us (Enumerator: Present antibiotic library to responder). We will be asking you to sort these antibiotics into different piles. This is not a test of your knowledge, but to find out whether you recognise these drugs, and whether you have given them to your child. We are carrying out this exercise to help you remember which ones you might have given to your child.*

98 *Which of the antibiotics in front of you do you **recognise**? Please pick the ones you **recognise** and put them into one pile.*

Enumerator: The list below is representative of potentially available antibiotics; the actual list will vary according to local availability.

Antibiotic (Formulation)	*Recognise*	*Antibiotic (Formulation)*	*Recognise*
Amoxicillin (Tablets)	*No Yes*	*Cloxacillin (Tablets)*	*No Yes*

Supplementary file 4: **Case Report Form – Mangochi Survey**

Amoxicillin (Suspension)	*No*	*Yes*	*Cloxacillin (Suspension)*	*No*	*Yes*
Ampicillin (Tablets)	*No*	*Yes*	*Cotrimoxazole (Tablets)*	*No*	*Yes*
Azithromycin (Tablets)	*No*	*Yes*	*Cotrimoxazole (Suspension)*	*No*	*Yes*
Benzathene Penicillin (Injectable)	*No*	*Yes*	*Doxycycline (Tablets)*	*No*	*Yes*
Benzylpenicillin (Injectable)	*No*	*Yes*	*Erythromycin (Tablets)*	*No*	*Yes*
Cefalexin (Tablets)	*No*	*Yes*	*Erythromycin (Suspension)*	*No*	*Yes*
Cefixime (Tablets)	*No*	*Yes*	*Flucloxacillin (Tablets)*	*No*	*Yes*
Ceftriaxone (Injectable)	*No*	*Yes*	*Flucloxacillin / amoxicillin (Tablets)*	*No*	*Yes*
Cefuroxime (Tablets)	*No*	*Yes*	*Gentamicin (Injectable)*	*No*	*Yes*
Chloramphenicol (Tablets)	*No*	*Yes*	*Levofloxacin (Tablets)*	*No*	*Yes*
Chloramphenicol (Injectable)	*No*	*Yes*	*Metronidazole (Tablets)*	*No*	*Yes*
Ciprofloxacin (Tablets)	*No*	*Yes*	*Metronidazole (Suspension)*	*No*	*Yes*
Clarithromycin (Tablets)	*No*	*Yes*	*Norfloxacin / metronidazole (Tablets)*	*No*	*Yes*
Amoxicillin / clavulanic acid (Tablets)	*No*	*Yes*	*Ofloxacin / ornidazole (Tablets)*	*No*	*Yes*
Clindamycin (Tablets)	*No*	*Yes*	*Phenoxymethylpenicillin (Tablets)*	*No*	*Yes*
Clindamycin (Injectable)	*No*	*Yes*	*Tetracycline (Tablets)*	*No*	*Yes*

Enumerator: Remove the unrecognised pile and put the recognised pile in front of the respondent.

99 *Which of the antibiotics in front of you have you* ***ever given*** *to your child? Please pick the ones you have* ***ever given*** *to your child and put them into one pile.*

Antibiotic (Formulation)	*Recognise*		*Antibiotic (Formulation)*	*Recognise*	
Amoxicillin (Tablets)	*No*	*Yes*	*Cloxacillin (Tablets)*	*No*	*Yes*
Amoxicillin (Suspension)	*No*	*Yes*	*Cloxacillin (Suspension)*	*No*	*Yes*
Ampicillin (Tablets)	*No*	*Yes*	*Cotrimoxazole (Tablets)*	*No*	*Yes*
Azithromycin (Tablets)	*No*	*Yes*	*Cotrimoxazole (Suspension)*	*No*	*Yes*
Benzathene Penicillin (Injectable)	*No*	*Yes*	*Doxycycline (Tablets)*	*No*	*Yes*
Benzylpenicillin (Injectable)	*No*	*Yes*	*Erythromycin (Tablets)*	*No*	*Yes*
Cefalexin (Tablets)	*No*	*Yes*	*Erythromycin (Suspension)*	*No*	*Yes*
Cefixime (Tablets)	*No*	*Yes*	*Flucloxacillin (Tablets)*	*No*	*Yes*
Ceftriaxone (Injectable)	*No*	*Yes*	*Flucloxacillin / amoxicillin (Tablets)*	*No*	*Yes*
Cefuroxime (Tablets)	*No*	*Yes*	*Gentamicin (Injectable)*	*No*	*Yes*
Chloramphenicol (Tablets)	*No*	*Yes*	*Levofloxacin (Tablets)*	*No*	*Yes*
Chloramphenicol (Injectable)	*No*	*Yes*	*Metronidazole (Tablets)*	*No*	*Yes*
Ciprofloxacin (Tablets)	*No*	*Yes*	*Metronidazole (Suspension)*	*No*	*Yes*
Clarithromycin (Tablets)	*No*	*Yes*	*Norfloxacin / metronidazole (Tablets)*	*No*	*Yes*
Amoxicillin / clavulanic acid (Tablets)	*No*	*Yes*	*Ofloxacin / ornidazole (Tablets)*	*No*	*Yes*
Clindamycin (Tablets)	*No*	*Yes*	*Phenoxymethylpenicillin (Tablets)*	*No*	*Yes*
Clindamycin (Injectable)	*No*	*Yes*	*Tetracycline (Tablets)*	*No*	*Yes*

Enumerator: Remove the unused antibiotics and put the used antibiotics in front of the respondent.

100 *Which of the antibiotics in front of you have you to your child in the* ***last 12 months****? Please pick the ones you have given to your child in the* ***last 12 months*** *and put them into one pile.*

Antibiotic (Formulation)	*Recognise*		*Antibiotic (Formulation)*	*Recognise*	
Amoxicillin (Tablets)	*No*	*Yes*	*Cloxacillin (Tablets)*	*No*	*Yes*
Amoxicillin (Suspension)	*No*	*Yes*	*Cloxacillin (Suspension)*	*No*	*Yes*
Ampicillin (Tablets)	*No*	*Yes*	*Cotrimoxazole (Tablets)*	*No*	*Yes*
Azithromycin (Tablets)	*No*	*Yes*	*Cotrimoxazole (Suspension)*	*No*	*Yes*
Benzathene Penicillin (Injectable)	*No*	*Yes*	*Doxycycline (Tablets)*	*No*	*Yes*

Singleton D, *et al. BMJ Open* 2023; 13:e069560. doi: 10.1136/bmjopen-2022-069560

Supplementary file 4: **Case Report Form – Mangochi Survey**

Benzylpenicillin (Injectable)	*No Yes*	*Erythromycin (Tablets)*	*No Yes*
Cefalexin (Tablets)	*No Yes*	*Erythromycin (Suspension)*	*No Yes*
Cefixime (Tablets)	*No Yes*	*Flucloxacillin (Tablets)*	*No Yes*
Ceftriaxone (Injectable)	*No Yes*	*Flucloxacillin / amoxicillin (Tablets)*	*No Yes*
Cefuroxime (Tablets)	*No Yes*	*Gentamicin (Injectable)*	*No Yes*
Chloramphenicol (Tablets)	*No Yes*	*Levofloxacin (Tablets)*	*No Yes*
Chloramphenicol (Injectable)	*No Yes*	*Metronidazole (Tablets)*	*No Yes*
Ciprofloxacin (Tablets)	*No Yes*	*Metronidazole (Suspension)*	*No Yes*
Clarithromycin (Tablets)	*No Yes*	*Norfloxacin / metronidazole (Tablets)*	*No Yes*
Amoxicillin / clavulanic acid (Tablets)	*No Yes*	*Ofloxacin / ornidazole (Tablets)*	*No Yes*
Clindamycin (Tablets)	*No Yes*	*Phenoxymethylpenicillin (Tablets)*	*No Yes*
Clindamycin (Injectable)	*No Yes*	*Tetracycline (Tablets)*	*No Yes*

Enumerator: Remove the unused antibiotics and put the used antibiotics in front of the respondent.

101 *Which of the antibiotics in front of you have you to your child in the **last 3 months**? Please pick the ones you have given to your child in the **last 3 months** and put them into one pile.*

Antibiotic (Formulation)	*Recognise*	*Antibiotic (Formulation)*	*Recognise*
Amoxicillin (Tablets)	*No Yes*	*Cloxacillin (Tablets)*	*No Yes*
Amoxicillin (Suspension)	*No Yes*	*Cloxacillin (Suspension)*	*No Yes*
Ampicillin (Tablets)	*No Yes*	*Cotrimoxazole (Tablets)*	*No Yes*
Azithromycin (Tablets)	*No Yes*	*Cotrimoxazole (Suspension)*	*No Yes*
Benzathene Penicillin (Injectable)	*No Yes*	*Doxycycline (Tablets)*	*No Yes*
Benzylpenicillin (Injectable)	*No Yes*	*Erythromycin (Tablets)*	*No Yes*
Cefalexin (Tablets)	*No Yes*	*Erythromycin (Suspension)*	*No Yes*
Cefixime (Tablets)	*No Yes*	*Flucloxacillin (Tablets)*	*No Yes*
Ceftriaxone (Injectable)	*No Yes*	*Flucloxacillin / amoxicillin (Tablets)*	*No Yes*
Cefuroxime (Tablets)	*No Yes*	*Gentamicin (Injectable)*	*No Yes*
Chloramphenicol (Tablets)	*No Yes*	*Levofloxacin (Tablets)*	*No Yes*
Chloramphenicol (Injectable)	*No Yes*	*Metronidazole (Tablets)*	*No Yes*
Ciprofloxacin (Tablets)	*No Yes*	*Metronidazole (Suspension)*	*No Yes*
Clarithromycin (Tablets)	*No Yes*	*Norfloxacin / metronidazole (Tablets)*	*No Yes*
Amoxicillin / clavulanic acid (Tablets)	*No Yes*	*Ofloxacin / ornidazole (Tablets)*	*No Yes*
Clindamycin (Tablets)	*No Yes*	*Phenoxymethylpenicillin (Tablets)*	*No Yes*
Clindamycin (Injectable)	*No Yes*	*Tetracycline (Tablets)*	*No Yes*

Enumerator: Remove the unused antibiotics and put the used antibiotics in front of the respondent.

102 *Which of the antibiotics in front of you have you to your child in the **last 14 days**? Please pick the ones you have given to your child in the **last 14 days** and put them into one pile.*

Antibiotic (Formulation)	*Recognise*	*Antibiotic (Formulation)*	*Recognise*
Amoxicillin (Tablets)	*No Yes*	*Cloxacillin (Tablets)*	*No Yes*
Amoxicillin (Suspension)	*No Yes*	*Cloxacillin (Suspension)*	*No Yes*
Ampicillin (Tablets)	*No Yes*	*Cotrimoxazole (Tablets)*	*No Yes*
Azithromycin (Tablets)	*No Yes*	*Cotrimoxazole (Suspension)*	*No Yes*
Benzathene Penicillin (Injectable)	*No Yes*	*Doxycycline (Tablets)*	*No Yes*
Benzylpenicillin (Injectable)	*No Yes*	*Erythromycin (Tablets)*	*No Yes*
Cefalexin (Tablets)	*No Yes*	*Erythromycin (Suspension)*	*No Yes*
Cefixime (Tablets)	*No Yes*	*Flucloxacillin (Tablets)*	*No Yes*
Ceftriaxone (Injectable)	*No Yes*	*Flucloxacillin / amoxicillin (Tablets)*	*No Yes*

Singleton D, *et al. BMJ Open* 2023; 13:e069560. doi: 10.1136/bmjopen-2022-069560

Supplementary file 4: **Case Report Form – Mangochi Survey**

Cefuroxime (Tablets)	*No* *Yes*		*Gentamicin (Injectable)*	*No* *Yes*	
Chloramphenicol (Tablets)	*No* *Yes*		*Levofloxacin (Tablets)*	*No* *Yes*	
Chloramphenicol (Injectable)	*No* *Yes*		*Metronidazole (Tablets)*	*No* *Yes*	
Ciprofloxacin (Tablets)	*No* *Yes*		*Metronidazole (Suspension)*	*No* *Yes*	
Clarithromycin (Tablets)	*No* *Yes*		*Norfloxacin / metronidazole (Tablets)*	*No* *Yes*	
Amoxicillin / clavulanic acid (Tablets)	*No* *Yes*		*Ofloxacin / ornidazole (Tablets)*	*No* *Yes*	
Clindamycin (Tablets)	*No* *Yes*		*Phenoxymethylpenicillin (Tablets)*	*No* *Yes*	
Clindamycin (Injectable)	*No* *Yes*		*Tetracycline (Tablets)*	*No* *Yes*	
If any antibiotics are in the given in the last 14 days pile, the child is ***ineligible*** *for study participation.*					

Nasal Sample Collection:

103	*NP swab collected?*	***No*** ***Yes***
104	*If no swab was collected, specify why not.*	
105	*Was the sample you collected 'adequate'?*<br>*(Adequate: swab passed to the back of nasopharynx for at least 3 seconds and twisted 360°)*	***No*** ***Yes***
106	*Is there nasal mucus on swab?*	***No*** ***Yes***

107	*Scan/enter the Lab barcode*	

Rectal Sample Collection:

108	*Rectal swab collected?*	***No*** ***Yes***
109	*If no swab was collected, specify why not.*	
110	*Was the sample you collected 'adequate'?*<br>*(Adequate: swab passed to the rectum for at least 3 seconds and twisted 360°)*	***No*** ***Yes***
111	*Is there faeces on swab?*	***No*** ***Yes***

112	*Scan/enter the Lab barcode*	

113	*Form completed by (Enumerator Code):*	*Code*	|___|___|___|
114	*Form completed by:*	*Signature*	

Singleton D, *et al. BMJ Open* 2023; 13:e069560. doi: 10.1136/bmjopen-2022-069560
