# Supplementary material for: Cross-sectional health centre and community-based evaluation of the impact of pneumococcal and malaria vaccination on antibiotic prescription and usage, febrile illness and antimicrobial resistance in young children in Malawi: the IVAR study protocol
Source: BMJ Open. 2023 May 12;13(5):e069560. doi: 10.1136/bmjopen-2022-069560 (PMC10186476; doi:10.1136/bmjopen-2022-069560)
Supplement: Author's manuscript [file bmjopen-2022-069560.draft_revisions.pdf]

BMJ Open is committed to open peer review. As part of this commitment we make the peer review history of every article we publish publicly available.

When an article is published we post the peer reviewers' comments and the authors' responses online. We also post the versions of the paper that were used during peer review. These are the versions that the peer review comments apply to.

The versions of the paper that follow are the versions that were submitted during the peer review process. They are not the versions of record or the final published versions. They should not be cited or distributed as the published version of this manuscript.

BMJ Open is an open access journal and the full, final, typeset and author-corrected version of record of the manuscript is available on our site with no access controls, subscription charges or pay-per-view fees (http://bmjopen.bmj.com).

If you have any questions on BMJ Open's open peer review process please email info.bmjopen@bmj.com

BMJ Open

The impact of pneumococcal and malaria vaccination on antibiotic prescription and usage, febrile illness and antimicrobial resistance in young children in Malawi: The IVAR Study protocol

Journal:	*BMJ Open*
Manuscript ID	bmjopen-2022-069560
Article Type:	Protocol
Date Submitted by the Author:	26-Oct-2022

Complete List of Authors:	Singleton, David; University of Liverpool<br>Ibarz-Pavon, Ana; University of Liverpool; Kamuzu University of Health Sciences, Malawi-Liverpool-Wellcome Trust Clinical Research Programme<br>Swarthout, Todd; UCL, Division of Infection and Immunity; Kamuzu University of Health Sciences, Malawi-Liverpool-Wellcome Trust Clinical Research Programme<br>Bonomali, Farouck; Malawi-Liverpool-Wellcome Trust Clinical Research Programme, Kamuzu University of Health Sciences<br>Cornick, Jennifer; University of Liverpool; Malawi-Liverpool-Wellcome Trust Clinical Research Programme<br>Gori, Andrea; University College London, Research Department of Infection<br>Kalizang'oma, Akuzike; Kamuzu University of Health Sciences, Malawi-Liverpool-Wellcome Trust Clinical Research Programme; University College London, Research Department of Infection<br>Ntiza, Noah; Kamuzu University of Health Sciences, Malawi-Liverpool-Wellcome Trust Clinical Research Programme<br>Brown, Comfort; Kamuzu University of Health Sciences, Malawi-Liverpool-Wellcome Trust Clinical Research Programme<br>Chipatala, Raphael; Kamuzu University of Health Sciences, Department of Public Health<br>Nyangulu, Wongani; Kamuzu University of Health Sciences, Department of Public Health<br>Chirombo, James; Kamuzu University of Health Sciences, Malawi-Liverpool-Wellcome Trust Clinical Research Programme<br>Kawalazira, Gift; Ministry of Health<br>Chibowa, Henry; Mangochi District Council<br>Mwansambo, Charles; Ministry of Health<br>Maleta, Kenneth ; Kamuzu University of Health Sciences, Department of Public Health<br>French, Neil; University of Liverpool, Department of Clinical Infection, Microbiology & Immunology, Institute of Infection and Global Health<br>Heyderman, Robert; UCL, Division of Infection and Immunity

Keywords:	BACTERIOLOGY, EPIDEMIOLOGY, Clinical audit < HEALTH SERVICES ADMINISTRATION & MANAGEMENT, Protocols & guidelines < HEALTH SERVICES ADMINISTRATION & MANAGEMENT, IMMUNOLOGY, INFECTIOUS DISEASES

SCHOLARONE™
Manuscripts

The impact of pneumococcal and malaria vaccination on antibiotic prescription and usage, febrile illness and antimicrobial resistance in young children in Malawi: The IVAR Study protocol

David A. Singleton[1*#], Ana Ibarz-Pavon[1,2*], Todd D. Swarthout[2,3], Farouck Bonomali[2], Jennifer E. Cornick[1,2], Andrea Gori[3], Akuzike Kalizang'oma[2,3], Noah Ntiza[2], Comfort Brown[2], Raphael Chipatala[4], Wongani Nyangulu[4], James Chirombo[2], Gift Kawalazira[5], Henry Chibowa[6], Charles Mwansambo[7], Kenneth Maleta[4], Neil French[1¥*], Robert S. Heyderman[3¥]

Author Affiliations

[1] Institute of Infection, Veterinary, and Ecological Science, University of Liverpool, Liverpool, United Kingdom

[2] Malawi-Liverpool-Wellcome Research Programme, Kamuzu University of Health Sciences, Blantyre, Malawi

[3] Research Department of Infection, Division of Infection and Immunity, University College London, London, United Kingdom

[4] Department of Public Health, School of Public Health & Family Medicine, Kamuzu University of Health Sciences, Blantyre, Malawi

[5] Blantyre District Health Office, Blantyre District Council, Blantyre, Malawi

[6] Mangochi District Health Office, Mangochi District Council, Mangochi, Malawi

[7] Malawi Ministry of Health, Lilongwe, Malawi

[*] David A. Singleton and Ana Ibarz-Pavon have contributed equally to this manuscript

[¥] Neil French and Robert Heyderman have contributed equally to this manuscript

[#] Corresponding author: David A. Singleton, University of Liverpool, 8 West Derby Street, Liverpool, United Kingdom, L69 7BE; Email: D.A.Singleton@liverpool.ac.uk; Phone: +44 (0)7904 111125

For peer review only

Abstract

Introduction

Vaccination is a potentially critical component of efforts to arrest the development and dissemination of antimicrobial resistance, though little is known about the impact vaccination may have within low-and-middle-income countries. This study will evaluate the impact of vaccination on reducing the carriage prevalence of resistant *Streptococcus pneumoniae* and extended spectrum beta-lactamase-producing *Escherichia coli* and *Klebsiella* species. We will leverage two large ongoing cluster-randomised vaccine evaluations in Malawi assessing; firstly, the impact of adding a booster dose to the 13-valent pneumococcal conjugate vaccine (PCV13) schedule, and secondly, the introduction of the RTS,S/AS01 malaria vaccine.

Methods and analysis

Six cross-sectional surveys will be implemented within primary healthcare centres and their local communities: three surveys in Blantyre District (PCV13 component) and three surveys in Mangochi District (RTS,S/AS01 component). We will evaluate common motivators for antibiotic prescription (*e.g.*, febrile illness), antibiotic prescription practices, and AMR carriage in children ≤3 years. For the PCV13 component, surveys will be conducted 9, 18 and 33 months following a 3+0 to 2+1 schedule change. For the RTS,S/AS01 component, surveys will be conducted 32, 44 and 56 months post-RTS,S/AS01 introduction. Six health centres in each study component will be randomly selected for inclusion in this study. Between intervention arms, the primary outcome will be the difference in prevalence of penicillin non-susceptibility amongst *Streptococcus pneumoniae* nasopharyngeal carriage isolates in healthy children. The study is powered to detect an absolute change of 13 percentage points (%) in the primary outcome (*i.e.,* 35% vs 22% penicillin non-susceptibility).

Ethics and dissemination

This study has been approved by the Kamuzu University of Health Sciences (Ref: P01-21-3249), the University College London (Ref: 18331/002) and the University of Liverpool (Ref: 9908) Research Ethics Committees. Results will be disseminated to the Malawi Ministry of Health and World Health Organisation, and through peer-reviewed publications and conference presentations.

For peer review only

For peer review only - http://bmjopen.bmj.com/site/about/guidelines.xhtml

Article Summary

The strengths and limitations of this study are:

- The study builds upon two large-scale vaccine evaluations, leveraging the infrastructure, methodology, and community engagement developed by such evaluations.
- The study adapts a range of methodologies previously employed in Malawi, and where new methods have been devised it utilises established methods as comparators.
- This will be, to the authors' knowledge, the first study to evaluate the impact of the newly WHO-approved RTS,S/AS01 vaccine on antimicrobial prescription and usage, and AMR.
- This will also be the first study to evaluate the impact of varying PCV13 vaccine delivery schedules on antimicrobial prescription and usage, and AMR.
- There is a risk of contamination between intervention and non-intervention arms (*i.e.,* children receiving an RTS,S/AS01 vaccine who relocate to a zone where RTS,S/AS01 is not being introduced, or vice-versa). Study activities will monitor this in initial and subsequent surveys.

Current word count: 4000

Key Words: *Streptococcus pneumoniae*; malaria; 13-valent pneumococcal conjugate vaccine; PCV13; Malawi; Africa; children; pneumococcal conjugate vaccines; malaria vaccine; RST,S/AS01; antimicrobial resistance; antibiotic prescription; antibiotic use; antibiotic access

For peer review only - http://bmjopen.bmj.com/site/about/guidelines.xhtml

Introduction

Antimicrobial resistance (AMR) is a leading global health threat [1], with 1.27 million deaths being attributable to AMR in 2019 alone [2]. AMR development is thought to be primarily driven by antimicrobial use [3], but with resistant genes and their host bacteria capable of passing between people, animals, and the environment [4] multi-faceted approaches are needed if we are to curb AMR development and dissemination [4]. In 2016 a global review on AMR set out ten recommendations for tackling this global pandemic [5], one of which was vaccination [5,6].

Vaccines may directly and indirectly impact on AMR [7]. Directly, vaccines target bacterial species with emerging clinical resistance issues (*e.g., Streptococcus pneumoniae, Salmonella typhi*) [8–10]. However, vaccines that target viruses and parasites may also deliver an indirect effect on AMR [7,11]. This may be *via* (i) removal of a 'gateway' pathogen for bacterial infection, (ii) improving general health, or (iii) reducing frequency of symptoms commonly associated with antibiotic prescription (*e.g.,* fever) [12], thereby reducing selection pressure for AMR development [7,11]. Conversely, vaccines may also exert a resistance selection pressure on target and/or 'bystander' pathogens [13,14]. Thus, for us to understand these complex interactions more fully, it is crucial that the putative impact of vaccines on AMR is evaluated in a systematic manner.

Varying responses to vaccination have been observed between High Income (HIC) and Low-and-Middle-Income Countries (LMICs), with vaccines frequently under-performing expectations in LMICs despite high vaccine coverage rates [15]. However, many LMICs also have severely limited access to diagnostics and appropriate antibiotics [16], with empirical and potentially unnecessary antibiotic prescriptions being a common reality of clinical practice [17]. Hence, whilst there is an intrinsic need to conduct vaccine impact evaluations in both HICs and LMICs, there is also a need to evaluate whether vaccination can play a cost effective [18] role in assisting equitable provision of antibiotics to those at greatest need.

Pneumococcal conjugate vaccines (PCVs) have arguably received the most attention of any vaccine regarding their potential to reduce AMR [7]. In the USA and the UK, the introduction of 7-valent (PCV7) and, later, 13-valent (PCV13) vaccines were associated with considerable

For peer review only - http://bmjopen.bmj.com/site/about/guidelines.xhtml

reductions in resistant pneumococcal infections [19–21]. Malawi introduced PCV13 in November 2011 using a 3+0 delivery schedule (1 dose at 6, 10 and 14 weeks of age), with vaccine coverage exceeding 90% [22]. Though introduction was associated with significant reductions in invasive pneumococcal disease [23] and all-cause mortality [24], vaccine serotype carriage has remained persistently high [25], and penicillin non-susceptibility in both carriage [15,26] and disease samples [27] has not decreased, particularly in non-vaccine serotypes [15]. Hence, some have queried whether PCV13 delivery can be further optimised [28]. The two PCV13 delivery schedules recommended by WHO include a 3+0 and a 2+1 (1 dose at 6 and 14 weeks of age and a booster at 9 months of age) schedule [29]. In 2021 a pragmatic, cluster-randomised evaluation on the impact on pneumococcal carriage of changing the existing 3+0 PCV13 delivery schedule to a 2+1 schedule was implemented in the Blantyre district of Malawi (known as the 'PAVE' study) [30]; our study will leverage the PAVE study to also assess the impact of delivery schedule change on AMR.

In 2021, RTS,S/AS01 became the first malaria vaccine to be recommended by the WHO for widespread use in young children [31]. A subunit vaccine targeting *Plasmodium falciparum* (*P. falciparum*), it is delivered via three doses at five, six and seven months of age followed by a fourth dose at 18-21 months of age [32]. Phase III trials indicated that the RTS,S/AS01 vaccine was effective at reducing clinical malaria [33]. However, although rare, increases in febrile convulsions, meningitis, cerebral malaria, and mortality rates in RTS,S/AS01 vaccinated individuals [34] led to a recommendation for further safety profiling and impact assessment [32]. Following this, the WHO announced phase IV evaluations in selected areas in Ghana, Kenya and Malawi (known as the 'EPI-MAL' study) [32]. We hypothesised that reductions in malaria-associated febrile illness in young children may be associated with reductions in antibiotic use and therefore AMR [12]. RTS,S/AS01 vaccination may also be associated with generalised improvements in health, reducing antibiotic exposure [7,11,12], and this study will work in conjunction with the ongoing phase IV evaluations in the Mangochi district of Malawi [32] to assess these hypotheses.

Hence, the "Impact of Vaccines on Antimicrobial Resistance" (IVAR) study aims to leverage the PAVE and EPI-MAL studies to evaluate the impact of (i) changing from a 3+0 to a 2+1 PCV13 delivery schedule, and (ii) introducing a novel non-bacterial vaccine (RTS,S/AS01) on

For peer review only - http://bmjopen.bmj.com/site/about/guidelines.xhtml

antibiotic prescription, febrile illness, and AMR carriage in children ≤3 years of age in Malawi. The IVAR study will also deploy methods for measuring antibiotic prescription and use in primary healthcare and community settings. As such, this study addresses three key research questions:

1) Can vaccination reduce the prevalence of antibiotic-resistant pneumococcal and extended spectrum beta-lactamase-producing (ESBL) *Escherichia coli (E. coli)* and *Klebsiella* species carriage in healthy children?
2) Can vaccination reduce incidence of febrile illness?
3) Can vaccination reduce incidence of antibiotic prescription?

Methods and analysis

Study setting

The IVAR study, in conjunction with the PAVE study [30] (ClinicalTrials.gov NCT04078997, implemented 16 March 2021), will evaluate the impact on AMR of changing the 3+0 to a 2+1 delivery schedule in children ≤3 years of age in the Blantyre District of southern Malawi (Figure 1) [30]. Blantyre District is a mixed urban and rural setting composed of 1.3 million residents over 1,800km^2, with children under the age of five comprising 16% of the population [35]. Throughout the district there are 28 governmental primary care Health Centres (HCs) [30] which serve defined catchment areas (Figure 2). The Malawi Ministry of Health and Blantyre District Health Office (DHO) have randomly selected ten HCs to implement a WHO-approved 2+1 PCV13 delivery schedule (intervention arm), with ten other HCs randomly selected to continue the 3+0 schedule and serve as the comparator arm [30].

The IVAR Study, in collaboration with an ongoing RTS,S/AS01 phase IV evaluation (ClinicalTrials.gov NCT03806465; RTS,S/AS01 introduced April 2019) will also work to evaluate the impact of RTS,S/AS01 on AMR in Mangochi District, southern Malawi (Figure 1). Mangochi District is rural and is composed of 1.1 million residents over 6,700km^2, with children under the age of five comprising 18% of the population [35]. Throughout the district there are 29 governmental primary care HCs [36] which serve defined catchment areas (Figure 3). For IVAR, RTS,S/AS01-exposed (*n*=5 HCs) and non-exposed (*n*=3 HCs) clusters have been selected, with respective HCs being geographically adjacent to each other (*i.e.,* exposed HCs are in

For peer review only - http://bmjopen.bmj.com/site/about/guidelines.xhtml

Mangochi township itself or within <13 miles of the town, whereas non-exposed HCs are 14-38 miles outside of Mangochi township) [32].

Study design

There will be six HC- and community-based cross-sectional surveys, split between the PCV13 (*n*=3 surveys) and the RTS,S/AS01 component (*n*=3 surveys) evaluations. For the PCV13 component, surveys will be conducted 9-, 18- and 33-months post-schedule change implementation. For the RTS,S/AS01 component, surveys will be conducted 32, 44 and 56 months post-introduction. The surveys will be composed of two concurrent data collection activities. Firstly, a community-based carriage survey of healthy children will be implemented, with collection of biological samples (nasopharyngeal and rectal swabs) and collection of information pertaining to the history of the child's febrile illness, malaria and antibiotic prescription history. Secondly, we will implement an anonymised audit of malaria rapid diagnostic test (mRDT) use and medicinal prescriptions (with a focus on antibiotics) in children presenting unwell to the outpatient department (OPD) of HCs, as recorded within each child's Health Passport (HP). Whilst for all surveys the HC audit will summarise children ≤3 years of age, the age-based eligibility of the community carriage survey will vary according to survey and vaccine evaluation; a study sampling frame is included in Figure 4.

Primary objectives

The primary objective is to evaluate the reduction in carriage prevalence of penicillin resistant *S. pneumoniae* (see section 2c for further details), ESBL *E.coli* and *Klebsiella* species following a PCV13 schedule change (Blantyre District) and, separately, following RTS,S/AS01 vaccine introduction (Mangochi District). This will answer the question of whether vaccines can play a role in reducing the carriage prevalence of resistant pathogens in young children in LMICs.

Secondary objectives

The secondary objectives are to evaluate (a) incidence of febrile illness and antibiotic prescription and use (thus providing a mechanism for any reduction in AMR), (b) incidence of macrolide, tetracycline and trimethoprim-sulfamethoxazole non-susceptibility in *S. pneumoniae,* and (c) changes to the wider upper respiratory tract and gastrointestinal resistome variation following PCV13 schedule change and RTS,S/AS01 vaccine introduction.

For peer review only - http://bmjopen.bmj.com/site/about/guidelines.xhtml

Study site selection

For the PCV13 component of this study, we will randomly select six HCs for IVAR study inclusion, including *n*=3 HCs switching to 2+1 and *n*=3 HCs continuing to provide 3+0, stratified by setting (urban, peri-urban and rural HCs) (Figure 2). These will be selected from the ten 2+1 and ten 3+0 HCs that have already been selected for inclusion in the aforementioned PCV13 schedule change evaluation. Of note, a 3+1 PCV13 schedule was implemented amongst a subset of children following the schedule change, targeting children living in the catchment area of a 2+1 HC and who had received their first or second PCV13 primary doses prior to the HC implementing the schedule change [30]. These children will be eligible for recruitment.

For peer review only

For the RTS,S/AS01 component of this study, we will randomly select six HCs for IVAR study inclusion (*n*=3 RTS,S/AS01-exposed and *n*=3 non-exposed HCs) from the five RTS,S/AS01 exposed and three non-exposed HCs that have already been selected for the RTS,S/AS01 introduction evaluation [32], stratified by setting (urban, peri-urban and rural HCs) (Figure 3).

Data collection activities

(1) Health centre audit

Data will be collected on vaccine cluster designation (*i.e.,* receiving intervention or not), vaccine schedule compliance, medicines prescribed, and febrile illness presentation (using mRDT use as a proxy) during that visit via review of the child's HP. This audit is intended to assess the impact of PCV13 schedule change or RTS,S/AS01 introduction on mRDT use and antibiotic prescription frequency.

a) Population and sampling

The audit will include children ≤3 years of age presenting to the OPD of selected HCs. Each HC will be assessed over at least two weeks during each survey; HCs in opposing study arms *(i.e.,* 2+1 *vs.* 3+0; RTS,S/AS01 *vs.* no RTS,S/AS01) will be paired according to setting and will be surveyed concurrently or on adjacent weeks depending on staff availability and local conditions (including weather patterns and related accessibility). Children's HPs will be reviewed upon HC exit. Study teams will aim to review the HP of every eligible child; however,

For peer review only - http://bmjopen.bmj.com/site/about/guidelines.xhtml

to avoid disrupting HC workflow, during busy periods study teams will not request that potentially eligible children wait until a member of the study team is available to review their HP.

b) Inclusion and exclusion criteria

Inclusion criteria will be: (1) ≤3 years of age, (2) HC OPD attendance for investigation and/or treatment of ill health, (3) verbal consent has been provided by parent/caregiver for HP review, and (4) HP available for review. Exclusion criteria will be: (1) HC attendance for vaccination, and (2) HC attendance for routine health examination (*e.g.,* weighing) and found to be well.

c) Intervention

Children's HPs will be reviewed upon children exiting selected HCs, having presented to the OPD.

d) Expected outcomes

The primary outcome will be the difference in antibiotic prescription incidence between intervention arms, measured as a proportion of total OPD visits of children ≤3 years of age. A secondary outcome will be difference in mRDT use, again as a proportion of total OPD visits. In this study, we use mRDT as a proxy for febrile illness. Additional outcomes include sub-analyses by specific antibiotic agent/class.

e) Study power and sample size calculation

A previous HC OPD review estimated that 70% of children under five years of age were prescribed antibiotics (Priyanka Patel, MLW, personal communication). Hence, the primary outcome is powered to detect a difference of 5% in antibiotic prescription incidence between intervention groups (65% *vs*. 70%). Two-tailed sample sizes were calculated setting confidence at 95% and power at 80%, indicating that 1,417 HPs would be required in each study arm. Hence, we will review 3,000 HPs per survey (*n*=18,000 for total study). HC-level sample sizes will be weighted according to estimated or actual population sizes within their respective catchment areas [32].

For peer review only - http://bmjopen.bmj.com/site/about/guidelines.xhtml

f) Informed consent process

Due to an anticipated high participation rate, we have developed a method to maximise efficiency in attaining verbal consent and capturing the needed information. Parents/caregivers will receive verbal (and written if they wish) information about the study activity, and will have the opportunity to ask questions and express their doubts and concerns before accepting to take part. Verbal parent/caregiver consent will then be provided voluntarily if they choose to participate.

g) Data collection, management and anonymisation procedures

Data will be collected using password-protected electronic data capturing. Participation will be completely anonymised, with no personal data collected. Data will be uploaded daily to a secured on-site server, which is backed up daily to both local and off-site facilities.

h) Statistical analyses

Only categorical variables will be collected; these will be defined by frequency distributions. Descriptive analyses encompassing both outcomes and vaccination status will be performed. Mixed effects logistic regression models investigating presence of (1) antibiotic prescription and (2) mRDT use on the day of HC attendance in individual children as outcome variables will be implemented. HC will be modelled as a random effect, with findings being balanced against the opposing outcome variable; vaccination status; other medicinal prescriptions; HC setting, and month of HC visit. Individual antibiotic classes/agents may also be explored as sub-analyses. Data collected here will be anonymously compared against routine attendance registry data collected by selected HCs.

(2) Community carriage survey

The community carriage surveys will focus on healthy children (Figure 4). Nasopharyngeal and rectal swab samples will be collected in addition to demographic data, vaccine compliance, febrile illness, history of mRDT use and medicine prescription and use history. These data will be informed via direct parent/caregiver questioning, review of the child's HP, and an antibiotic provision recall exercise known as the 'drug bag method' [16].

a) Population and sampling

For peer review only - http://bmjopen.bmj.com/site/about/guidelines.xhtml

A stratified convenience sampling approach will be applied, making use of available local census data and utilising networks of Health Surveillance Assistants (HSAs) and health volunteers when needed. The sampling approach is intended to maximise efficiency in recruiting children who have received the appropriate intervention for the HC catchment area in which they reside. Hence, sampling will identify villages that are most proximal to HCs and will work to recruit all eligible children within those villages. Additional villages will be approached if needed until sampling targets are met.

i. PCV13 component

Children aged 4-9 months will be sampled in the first survey to establish the baseline difference between children provided with three (3+0 schedule) or two (2+1 schedule) primary PCV13 doses. Children residing in 2+1 catchment areas and who have received 3 primary PCV13 doses (referred to as '3+1 children') will be eligible for study inclusion. In the second and third surveys, children 15-24 months of age will be sampled to compare children who have received a booster dose (2+1) against those who have not (3+0).

There is limited census data available in these settings. Thus, we will work closely with local HSAs and health volunteers who will assist with development of community engagement strategies (including communication with community leaders), identify eligible children, locate targeted households, and facilitate communication with household members.

ii. RTS,S/AS01 component

Children aged 18-24 months of age will be sampled across all three surveys to compare children who have received the RTS,S/AS01 vaccine to unvaccinated children. Recent censuses have been completed to support the RTS,S/AS01 evaluation[32], providing greater confidence in local population estimates. Hence, parents/caregivers of potentially eligible children will be located and contacted directly by study teams.

b) Inclusion and exclusion criteria

For both the PCV13 and RTS,S/AS01 components, inclusion criteria includes (1) Age of child within the range determined by the particular survey (Figure 4), (2) permanent residence in the relevant study site, (3) parent/caregiver providing written informed consent, (4) evidence

For peer review only - http://bmjopen.bmj.com/site/about/guidelines.xhtml

of having received a full initial (primary dose/s) vaccine course particular to evaluation and study arm, and (5) that the child is healthy at time of sampling.

Exclusion criteria includes (1) child having received antibiotics within the previous 14 days, (2) child currently receiving tuberculosis treatment, (3) child having been hospitalised for pneumonia within the previous 14 days, (4) presence of gross respiratory pathology, (5) child having a terminal illness, (6) child previously recruited into the current survey, and (7) parent/caregiver not providing informed written consent.

For the second and third surveys of the PCV13 component, children residing in 2+1 clusters must have received the booster PCV13 dose. For the RTS,S/AS01 component, receipt of the booster dose is not a requirement in all three surveys, though provision will be recorded.

c) Sample collection & laboratory processing

For both the PCV13 and RTS,S/AS01 components, sample collection will include nasopharyngeal and rectal swabs from each participant. Following previously described WHO recommendations [25,37], nasopharyngeal swabs will be collected in skim milk-tryptone-glucose-glycerine (STGG) medium and rectal swabs will be collected in Cary-Blair medium, both being stored in -80°C freezers at the Malawi-Liverpool-Wellcome Research Programme (MLW) laboratory in Blantyre within 10 hours of collection for later batch-testing. Samples collected in Mangochi District will initially be taken to the Public Health and Nutrition Research Group (PHNG) laboratory, collocated to the Mangochi District Hospital, for storage prior to transport to MLW. Samples will be cultured to isolate and characterize *S. pneumoniae*, ESBL *E. coli* and *Klebsiella* species at MLW. Isolates will then be sent to the United Kingdom for Whole Genome Sequencing (WGS).

For *S. pneumoniae,* penicillin non-susceptibility will be defined genotypically, as a minimum inhibitory concentration (MIC) over 0.06 ml/L; 200 genotypically non-susceptible isolates will be phenotypically tested to confirm genotypic findings. Non-susceptibility to macrolides (azithromycin, MIC 0.25< mg/L), tetracyclines (doxycycline, MIC 1.00< mg/ML) and trimethoprim-sulfamethoxazole (MIC 1.00< mg/ML) will also be examined genotypically with phenotypic confirmation as secondary objectives. Sub-analyses will include determining

For peer review only

For peer review only - http://bmjopen.bmj.com/site/about/guidelines.xhtml

serotype of *S. pneumoniae isolates.* 800 carriage samples will be selected for broader resistome analyses.

E. coli and *Klebsiella* species ESBL status will be determined phenotypically via chromogenic ESBL media. Depending on resource availability, a proportion of isolates will also undergo WGS and broader resistome analyses.

d) Data collection

A range of demographic, mRDT use and medicine use history will be collected from the participants' health passport. These data will be supplemented with direct parent/caregiver questioning. The drug bag method will also be used; this has previously been deployed in Malawi [16] and we will re-purpose this approach to assist parent/caregiver recall in identifying the different antibiotics given to the participating child. In brief, prior to study initiation the study team will obtain antibiotics routinely used for systemic administration (including multiple formulations of the same agent where obtainable) and available for dispensing or sale in the community (including HCs, hospitals, private pharmacies and informal sources). Parents/caregivers will be asked if they recognise individual antibiotics (presented as pictures, example in Figure 5). For recognised antibiotics, parents/caregivers will be asked whether they have ever given the antibiotic to the participating child. If yes, they will be asked whether the antibiotic was given in the 12 months, 3 months or 14 days prior to recruitment (Figure 6). Findings from direct questioning, HP review and the drug bag exercise will be compared and combined to provide a more complete history of a participant's antibiotic exposure.

e) Primary and secondary outcomes

Carriage isolates: The primary outcome will be the difference in prevalence of penicillin non-susceptibility among *S. pneumoniae* carriage isolates, comparing i) the 2+1 vs. 3+0 intervention arms of the PCV13 component) and ii) the RTS,S/AS01 vs. no-RTS,S/AS01 intervention arms of the RTS,S/AS01 component. Secondary outcomes will also encompass macrolide, tetracycline and trimethoprim-sulfamethoxazole non-susceptibility.

For peer review only

Rectal swabs: The primary outcome will be the difference in prevalence of ESBL *E. coli* and *Klebsiella* species carriage comparing i) the 2+1 vs. 3+0 arms of the PCV13 component) and ii) the RTS,S/AS01 vs. no-RTS,S/AS01 arms of the RTS,S/AS01 component.

Antibiotic usage: The primary outcome will be the difference in incidence of antibiotic prescription intended for systemic administration from 14 days to three months prior to recruitment, comparing i) the 2+1 vs. 3+0 arms of the PCV13 component) and ii) the RTS,S/AS01 vs. no-RTS,S/AS01 arms of the RTS,S/AS01 component. Additional outcomes will include sub-analyses by specific antibiotic agent/class.

Febrile illness: A secondary outcome will be the difference in incidence of febrile illness from 14 days to three months prior to recruitment, comparing i) the 2+1 vs. 3+0 arms of the PCV13 component) and ii) the RTS,S/AS01 vs. no-RTS,S/AS01 arms of the RTS,S/AS01 component.

f) Study power and sample size calculation

Previous surveys have indicated a minimum pneumococcus carriage prevalence of 60% in children under the age of five [25], with 35% of such isolates expected to display penicillin non-susceptibility (unpublished data). Hence, the primary outcome is powered to detect a crude absolute decrease of 13 percentage points (13%) in penicillin non-susceptibility (*i.e.,* 35% *vs*. 22%). Two-tailed sample sizes were calculated setting confidence at 95% and power at 80%, indicating that 204 pneumococcus isolates would be required in each study arm per survey. Hence, allowing for a 60% carriage prevalence, 700 samples will be collected per component per survey (*n*=4,200 for total study). HC-level sample sizes will be weighted according to estimated or actual population sizes within their respective catchment areas [32].

A previous survey has indicated a minimum ESBL *E. coli* carriage of 25% in Blantyre (Nicholas Feasey, MLW, personal communication). Hence, the sample size per survey noted above will allow a crude absolute difference of 8 percentage points (8%) to be detected (*i.e.,* 25% *vs*. 17%) between study arms.

g) Informed consent process

For peer review only

For peer review only - http://bmjopen.bmj.com/site/about/guidelines.xhtml

Participant's parents/caregivers will receive written and verbal information about the study, and will have the opportunity to ask questions, express their doubts and concerns, and have time to reflect before deciding to take part or not. An informed consent form will be signed and dated by the participant's parent/caregiver and a member of the research team, a copy of which will be retained by the parent/caregiver. Participant's parents/caregivers may withdraw consent at any point without need to provide a reason, and without penalty.

h) Data collection, management and anonymisation procedures

Data will be collected using password-protected electronic data capturing. Each participant will be assigned a unique participant identification number (PID) at recruitment. This PID will be used in all datasheets and files, and will be linked to laboratory data, thus, only anonymised data will be used for analyses. A logbook containing identifiable information (including name) will be kept separately in a secured location and will only be accessed by authorised study team members. This will allow the study team to recover any missing epidemiological information, if necessary, later (*e.g.,* missing vaccination dates), and to facilitate any participant's parents/caregivers who wish to withdraw consent.

i) Statistical analyses

Continuous variables will be expressed as means and standard deviations, or medians and interquartile ranges. Categorical variables will be defined by frequency distributions. Descriptive analyses encompassing primary, secondary and additional outcomes, and vaccination status, will be performed. Mixed effects logistic regression models investigating prevalence of penicillin non-susceptible *S. pneumoniae* across all pneumococcus carriage isolates as an outcome variable will form the primary analysis. HC will be modelled as a random effect, with findings being balanced against key demographic variables (*i.e.,* age, sex etc.), vaccination status, antibiotic and febrile illness history, rurality, and the month in which sampling was completed. This approach will be repeated for other antibiotic classes, and for ESBL *E. coli* and *Klebsiella* species. We will also descriptively analyse antibiotic prescription and febrile illness history (using mRDT usage as a proxy), and may progress to inferential statistics if justified.

Ethics and dissemination

For peer review only - http://bmjopen.bmj.com/site/about/guidelines.xhtml

Ethical approval

This study has been approved by the Research Ethic Committees (REC) of Kamuzu University of Health Sciences (Ref: P01-21-3249), University College London (Ref: 18331/002) and the University of Liverpool (Ref: 9908) Research Ethics Committees.

Dissemination policy and plans

Study results will be shared with local communities and stakeholders, the Malawi Ministry of Health, other relevant policymakers and decision-making stakeholders, and published in peer-reviewed journals. Findings will be presented at international conferences and meetings. Copies of all published materials and reports will be shared with the research ethics committees and collaborators.

Data availability

Anonymised data, including bacterial genome sequencing data, used in analyses for reported findings of this study will be made publicly available. Human subject metadata will be shared primarily with scientific collaborators but, where appropriate and with the proper safeguards, made freely available to researchers with an established scientific record. To access human subject metadata, a formal project proposal would be made and reviewed by the investigators and the Kamuzu University of Health Sciences Research Ethics Committee. Following approval, data would be released subject to a signed data user agreement. Procedures for strain exchange, data sharing and ownership will follow Nagoya protocol standards [38].

Patient and public involvement statement

Prior to development of the protocol, key stakeholders were informed of the study, including the selected HCs and their surrounding communities, the District Health Office, Ministry of Health and the Ministry of Education. We actively sought and incorporated input from these stakeholders into the study objectives and overall design. Given that this study collaborates closely with two existing vaccine evaluations, pre-existing community engagement and sensitisation will strengthen community trust at the onset of this study. Especially in light of COVID-19, we envisage expanded community engagement prior to start of data collection, encompassing HCs, community leaders, HSAs, health volunteers, and members of the

For peer review only - http://bmjopen.bmj.com/site/about/guidelines.xhtml

communities themselves in information-providing activities. We will repeat community engagement activities prior to each survey, and provide feedback on prior surveys where possible.

Acknowledgements

The authors thank the MLW laboratory management team, led by Brigitte Denis and George Selemani. We also thank the MLW data management team, and would especially like to commemorate the memory of Clemens Masesa who led this team until his passing. We are further appreciative of the guidance and advice provided by Eleanor MacPherson, Priyanka Patel, Derek Cocker, and Nicholas Feasey. We would also like to thank a range of supporting team members at MLW and KUHeS, particularly Mernani Kaonga and our data collection teams, in addition to the many HC team members, HSAs, health volunteers, community leaders and community members who without their kind support and time this study would not be possible.

References

1 WHO. 10 global health issues to track in 2021. https://www.who.int/news-room/spotlight/10-global-health-issues-to-track-in-2021 (accessed 28 Feb 2022).

2 Murray CJL, Ikuta KS, Sharara F, *et al.* Global burden of bacterial antimicrobial resistance in 2019: a systematic analysis. *Lancet* 2022;**399**:629–55. doi:10.1016/S0140-6736(21)02724-0

3 Cantón R, Bryan J. Global antimicrobial resistance: from surveillance to stewardship. Part 1: surveillance and risk factors for resistance. *Expert Rev Anti Infect Ther* 2012;**10**:1269-1271 3p. doi:10.1586/eri.12.120

4 Wernli D, Jørgensen PS, Parmley EJ, *et al.* Evidence for action: a One Health learning platform on interventions to tackle antimicrobial resistance. *Lancet Infect Dis* 2020;**20**:e307–11. doi:https://doi.org/10.1016/S1473-3099(20)30392-3

5 O'Neill J. Tackling drug-resistant infections globally: final report and recommendations. 2016.http://amr-review.org/home

6 WHO. Resolution 72·5–antimicrobial resistance. 2019.https://apps.who.int/gb/ebwha/pdf_files/WHA72/A72_R5-en.pdf (accessed 11 May 2022).

For peer review only - http://bmjopen.bmj.com/site/about/guidelines.xhtml

7 Micoli F, Bagnoli F, Rappuoli R, *et al.* The role of vaccines in combatting antimicrobial resistance. *Nat Rev Microbiol* 2021;**19**:287–302. doi:10.1038/s41579-020-00506-3

8 Patel PD, Patel P, Liang Y, *et al.* Safety and Efficacy of a Typhoid Conjugate Vaccine in Malawian Children. *N Engl J Med* 2021;**385**:1104–15. doi:10.1056/NEJMoa2035916

9 Britto CD, Wong VK, Dougan G, *et al.* A systematic review of antimicrobial resistance in Salmonella enterica serovar Typhi, the etiological agent of typhoid. *PLoS Negl Trop Dis* 2018;**12**:e0006779.https://doi.org/10.1371/journal.pntd.0006779

10 Watkins ER, Kalizang'Oma A, Gori A, *et al.* Factors affecting antimicrobial resistance in Streptococcus pneumoniae following vaccination introduction. *Trends Microbiol* Published Online First: 6 September 2022. doi:10.1016/j.tim.2022.06.001

11 Marc L, R. SG, P. KK, *et al.* How Can Vaccines Contribute to Solving the Antimicrobial Resistance Problem? *MBio* 2022;**7**:e00428-16. doi:10.1128/mBio.00428-16

12 Maze MJ, Bassat Q, Feasey NA, *et al.* The epidemiology of febrile illness in sub-Saharan Africa: implications for diagnosis and management. *Clin Microbiol Infect* 2018;**24**:808–14. doi:https://doi.org/10.1016/j.cmi.2018.02.011

13 Christine T, W. OS, H. GY, *et al.* Estimating the proportion of bystander selection for antibiotic resistance among potentially pathogenic bacterial flora. *Proc Natl Acad Sci* 2018;**115**:E11988–95. doi:10.1073/pnas.1810840115

14 Cassiolato AP, Almeida SCG, Andrade AL, *et al.* Expansion of the multidrug-resistant clonal complex 320 among invasive Streptococcus pneumoniae serotype 19A after the introduction of a ten-valent pneumococcal conjugate vaccine in Brazil. *PLoS One* 2018;**13**:e0208211.https://doi.org/10.1371/journal.pone.0208211

15 Gori A, Obolski U, Swarthout TD, *et al.* The metabolic, virulence and antimicrobial resistance profiles of colonizing Streptococcus pneumoniae shift after pneumococcal vaccine introduction in urban Malawi. *medRxiv* 2021;:2021.07.21.21260914. doi:10.1101/2021.07.21.21260914

16 Dixon J, MacPherson E, Manyau S, *et al.* The 'Drug Bag' method: lessons from anthropological studies of antibiotic use in Africa and South-East Asia. *Glob Health Action* 2019;**12**:1639388. doi:10.1080/16549716.2019.1639388

17 MacPherson EE, Reynolds J, Sanudi E, *et al.* Understanding antimicrobial resistance through the lens of antibiotic vulnerabilities in primary health care in rural Malawi. *Glob Public Health* 2021;:1–17. doi:10.1080/17441692.2021.2015615

For peer review only - http://bmjopen.bmj.com/site/about/guidelines.xhtml

18 Sevilla JP, Bloom DE, Cadarette D, *et al.* Toward economic evaluation of the value of vaccines and other health technologies in addressing AMR. *Proc Natl Acad Sci* 2018;**115**:12911–9. doi:10.1073/pnas.1717161115

19 Kyaw MH, Lynfield R, Schaffner W, *et al.* Effect of Introduction of the Pneumococcal Conjugate Vaccine on Drug-Resistant Streptococcus pneumoniae. *N Engl J Med* 2006;**354**:1455–63. doi:10.1056/NEJMoa051642

20 Ladhani SN, Slack MPE, Andrews NJ, *et al.* Invasive pneumococcal disease after routine pneumococcal conjugate vaccination in children, England and Wales. *Emerg Infect Dis* 2013;**19**:61–8. doi:10.3201/eid1901.120741

21 Moore MR, Link-Gelles R, Schaffner W, *et al.* Effect of use of 13-valent pneumococcal conjugate vaccine in children on invasive pneumococcal disease in children and adults in the USA: analysis of multisite, population-based surveillance. *Lancet Infect Dis* 2015;**15**:301–9. doi:https://doi.org/10.1016/S1473-3099(14)71081-3

22 Tsega A, Hausi H, Chriwa G, *et al.* Vaccination coverage and timely vaccination with valid doses in Malawi. *Vaccine Reports* 2016;**6**:8–12. doi:https://doi.org/10.1016/j.vacrep.2016.06.001

23 Bar-Zeev N, Swarthout TD, Everett DB, *et al.* Impact and effectiveness of 13-valent pneumococcal conjugate vaccine on population incidence of vaccine and non-vaccine serotype invasive pneumococcal disease in Blantyre, Malawi, 2006–18: prospective observational time-series and case-control stud. *Lancet Glob Heal* 2021;**9**:e989–98. doi:10.1016/S2214-109X(21)00165-0

24 King C, Bar-Zeev N, Phiri T, *et al.* Population impact and effectiveness of sequential 13-valent pneumococcal conjugate and monovalent rotavirus vaccine introduction on infant mortality: prospective birth cohort studies from Malawi. *BMJ Glob Heal* Published Online First: 2020. doi:10.1136/bmjgh-2020-002669

25 Swarthout TD, Fronterre C, Lourenço J, *et al.* High residual carriage of vaccine-serotype Streptococcus pneumoniae after introduction of pneumococcal conjugate vaccine in Malawi. *Nat Commun* 2020;**11**:2222. doi:10.1038/s41467-020-15786-9

26 Lo SW, Gladstone RA, van Tonder AJ, *et al.* Pneumococcal lineages associated with serotype replacement and antibiotic resistance in childhood invasive pneumococcal disease in the post-PCV13 era: an international whole-genome sequencing study. *Lancet Infect Dis* 2019;**19**:759–69. doi:https://doi.org/10.1016/S1473-3099(19)30297-

For peer review only

For peer review only - http://bmjopen.bmj.com/site/about/guidelines.xhtml

X

27 Musicha P, Cornick JE, Bar-Zeev N, *et al.* Trends in antimicrobial resistance in bloodstream infection isolates at a large urban hospital in Malawi (1998–2016): a surveillance study. *Lancet Infect Dis* 2017;**17**:1042–52. doi:https://doi.org/10.1016/S1473-3099(17)30394-8

28 Lourenço J, Obolski U, Swarthout TD, *et al.* Determinants of high residual post-PCV13 pneumococcal vaccine-type carriage in Blantyre, Malawi: a modelling study. *BMC Med* 2019;**17**:219. doi:10.1186/s12916-019-1450-2

29 WHO. Pneumococcal vaccines WHO position paper – 2012 – Recommendations. *Vaccine* 2012;**30**:4717–8. doi:https://doi.org/10.1016/j.vaccine.2012.04.093

30 Swarthout TD, Ibarz-Pavon A, Kawalazira G, *et al.* A pragmatic health centre-based evaluation comparing the effectiveness of a PCV13 schedule change from 3+0 to 2+1 in a high pneumococcal carriage and disease burden setting in Malawi: a study protocol. *BMJ Open* 2021;**11**:e050312. doi:10.1136/bmjopen-2021-050312

31 WHO. WHO recommends groundbreaking malaria vaccine for children at risk. 2021.https://www.who.int/news/item/06-10-2021-who-recommends-groundbreaking-malaria-vaccine-for-children-at-risk (accessed 12 May 2022).

32 Praet N, Asante KP, Bozonnat M-C, *et al.* Assessing the safety, impact and effectiveness of RTS,S/AS01E malaria vaccine following its introduction in three sub-Saharan African countries: methodological approaches and study set-up. *Malar J* 2022;**21**:132. doi:10.1186/s12936-022-04144-3

33 RTS SCTP. Efficacy and safety of RTS,S/AS01 malaria vaccine with or without a booster dose in infants and children in Africa: final results of a phase 3, individually randomised, controlled trial. *Lancet* 2015;**386**:31–45. doi:10.1016/S0140-6736(15)60721-8

34 Guerra Mendoza Y, Garric E, Leach A, *et al.* Safety profile of the RTS,S/AS01 malaria vaccine in infants and children: additional data from a phase III randomized controlled trial in sub-Saharan Africa. *Hum Vaccin Immunother* 2019;**15**:2386–98. doi:10.1080/21645515.2019.1586040

35 National Statistical Office. 2018 Malawi Population and Housing Census. 2019.https://malawi.unfpa.org/sites/default/files/resource-pdf/2018 Malawi Population and Housing Census Main Report %281%29.pdf (accessed 4 Jul 2022).

For peer review only

For peer review only - http://bmjopen.bmj.com/site/about/guidelines.xhtml

36 Mamba KC, Muula AS, Stones W. Facility-imposed barriers to early utilization of focused antenatal care services in Mangochi District, Malawi – a mixed methods assessment. *BMC Pregnancy Childbirth* 2017;**17**:444. doi:10.1186/s12884-017-1631-y

37 Satzke C, Turner P, Virolainen-Julkunen A, *et al.* Standard method for detecting upper respiratory carriage of Streptococcus pneumoniae: Updated recommendations from the World Health Organization Pneumococcal Carriage Working Group. *Vaccine* 2013;**32**:165–79. doi:https://doi.org/10.1016/j.vaccine.2013.08.062

38 United Nations. Nagoya Protocol on Access to Genetic Resources and the Fair and Equitable Sharing of Benefits Arising from their Utilization (ABS) to the Convention on Biological Diversity. 2022.https://www.cbd.int/abs/about/ (accessed 7 Oct 2022).

Contributors

RSH, NF, KM, TDS, JEC and AG conceived the study. DAS, AI, RSH, NF, KM, TDS, JEC, AK and AG designed the study, with contributions from FB, NN, CG, RC, AK and WN. AI, TDS, JEC, CB and DAS oversaw development of laboratory methods. DAS, AI, FB and JEC designed study documents. JC conducted community mapping exercises. GK, HC and CM provided public health oversight and facilitated access to study sites. DAS and AI jointly wrote the first draft. All authors have read and approved the final manuscript.

Competing interests

The authors declare that they have no competing interests.

Funding

This work was funded by the Wellcome Trust (Ref: 219900/Z/19/Z) to RSH. The MLW Research Programme is supported by a Strategic Award from the Wellcome Trust, UK (206545/Z/17/Z). RSH is a National Institute of Health Research (NIHR) Senior Investigator. The views expressed in this publication are those of the authors and not necessarily those of the NIHR or the UK Department of Health and Social Care.

For peer review only

For peer review only - http://bmjopen.bmj.com/site/about/guidelines.xhtml

Figure 1: Malawi split by 28 district and city regions. Red shading indicates the Mangochi (bordering Lake Malawi, shaded in blue) and Blantyre districts (central Southern Malawi) where the IVAR study will be conducted.

Figure 2: Blantyre District with boundaries of 28 health centre catchment areas. Red cross = health centre. Blue and red shading indicate 2+1 and 3+0 PVC13 schedule areas randomly selected for this study, respectively. Green areas are non-inhabited (including mountains, industrial zones, and other regions administratively declared not for habitation). Adapted from Swarthout *et al.* (2021) [30].

Figure 3: Mangochi district with boundaries of 29 health centre catchment areas. Red cross = health centre. Red and yellow shading indicate RTS,S/AS01 malaria vaccine exposed and unexposed areas randomly selected for this study, respectively. Blue shading corresponds to Lake Malawi and Lake Malombe. Green areas are protected zones (e.g., wildlife reserves and national parks etc.).

Figure 4: IVAR study sampling frame. PCV = pneumococcal conjugate vaccine, RTS,S/AS01 = malaria vaccine, m=months, RDT = rapid diagnostic test.

Figure 5: Example of an antibiotic picture (azithromycin) which will be used in the drug bag exercise as part of the community carriage survey. Method adapted from Dixon *et al.* (2019)[16].

Figure 6: Flow diagram of questionnaire workflow of the 'drug bag' component of the community carriage survey. Each orange number corresponds to a round of questioning, starting with all locally identified, available antibiotics being presented to participant's parents/caregivers. The pool of antibiotics available for review is expected to diminish in each successive round, with the final round (round 5) being included as a final check that no child has been given antibiotics within the 14 days prior to sampling, which would render them ineligible for survey participation.

For peer review only

For peer review only - http://bmjopen.bmj.com/site/about/guidelines.xhtml

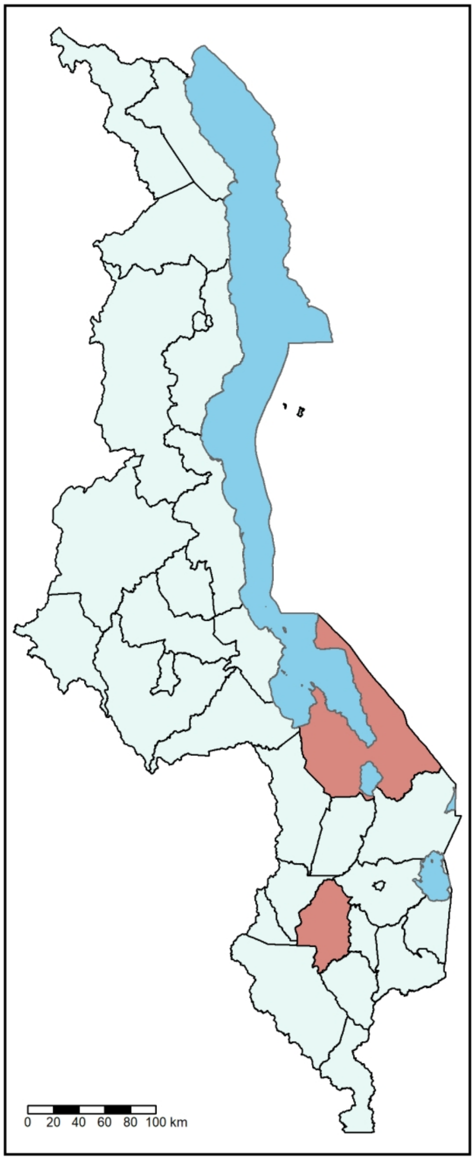

Malawi split by 28 district and city regions. Red shading indicates the Mangochi (bordering Lake Malawi, shaded in blue) and Blantyre districts (central Southern Malawi) where the IVAR study will be conducted.

254x508mm (118 x 118 DPI)

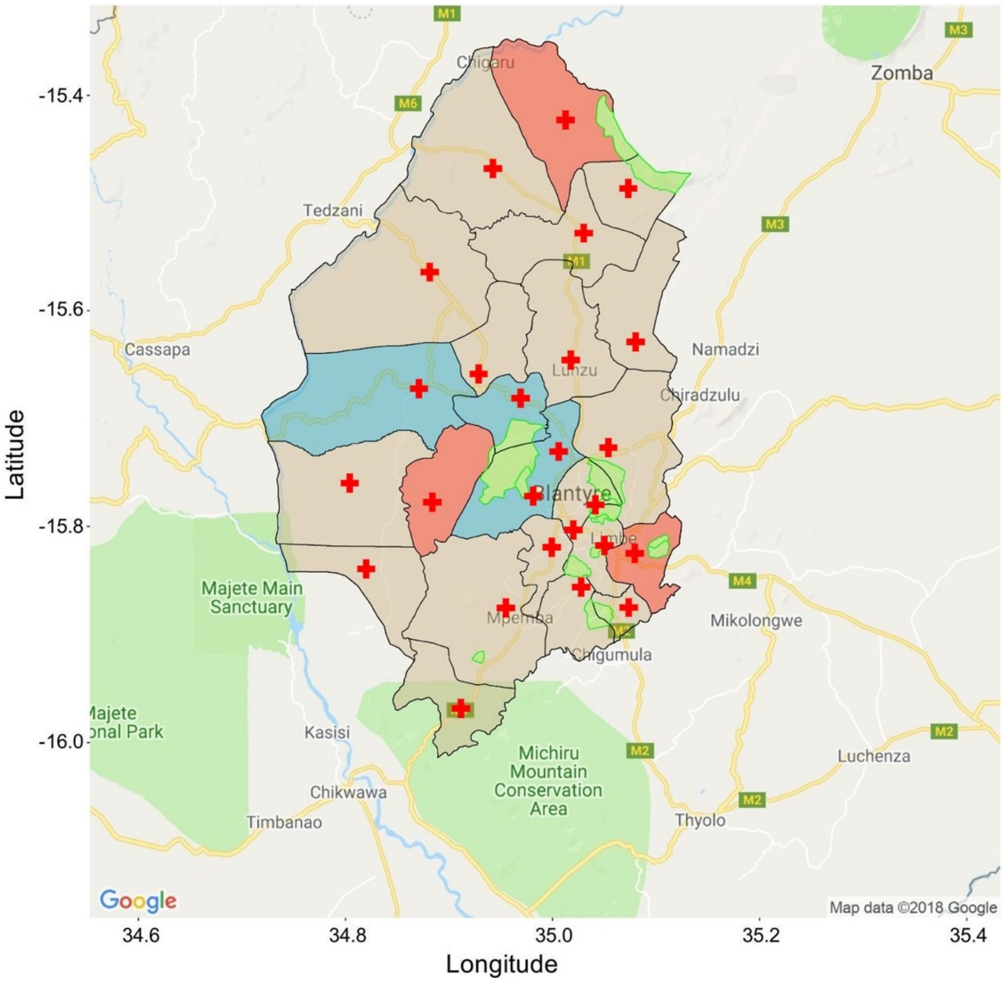

Blantyre District with boundaries of 28 health centre catchment areas. Red cross = health centre. Blue and red shading indicate 2+1 and 3+0 PVC13 schedule areas randomly selected for this study, respectively. Green areas are non-inhabited (including mountains, industrial zones, and other regions administratively declared not for habitation). Adapted from Swarthout et al. (2021) [30].

195x190mm (150 x 150 DPI)

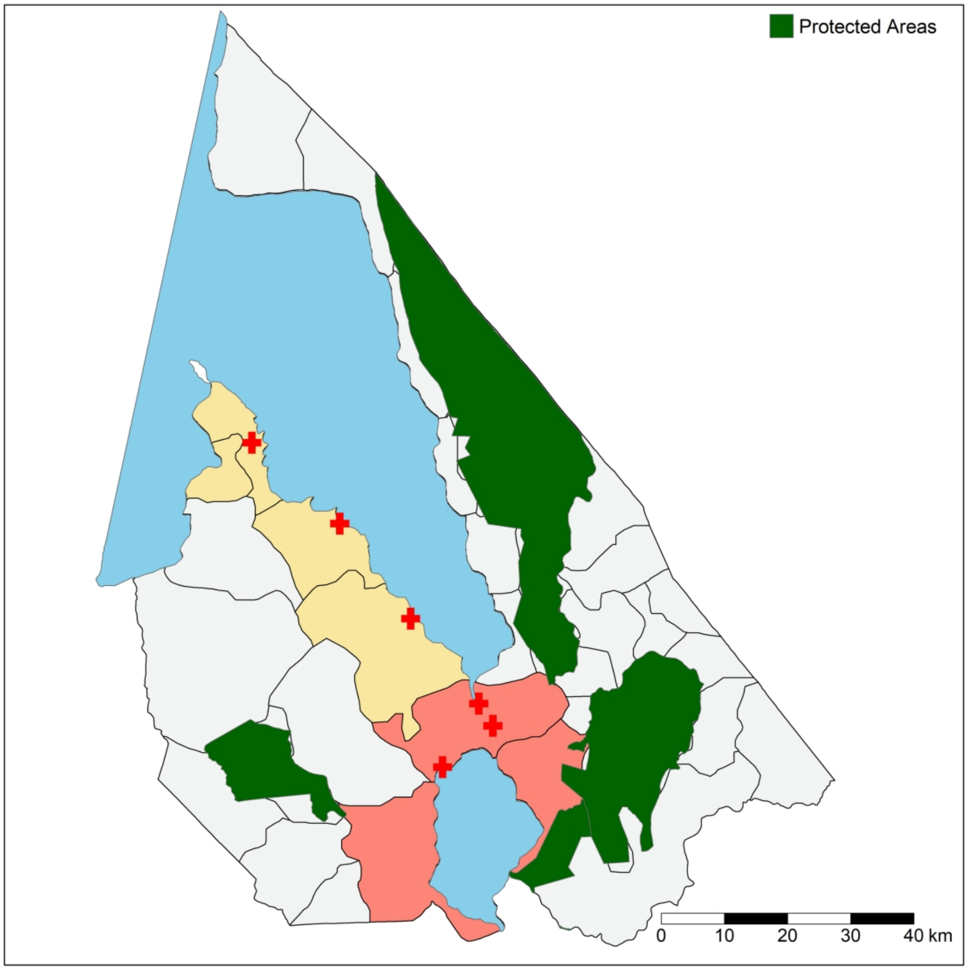

Mangochi district with boundaries of 29 health centre catchment areas. Red cross = health centre. Red and yellow shading indicate RTS,S/AS01 malaria vaccine exposed and unexposed areas randomly selected for this study, respectively. Blue shading corresponds to Lake Malawi and Lake Malombe. Green areas are protected zones (e.g., wildlife reserves and national parks etc.).

483x483mm (130 x 130 DPI)

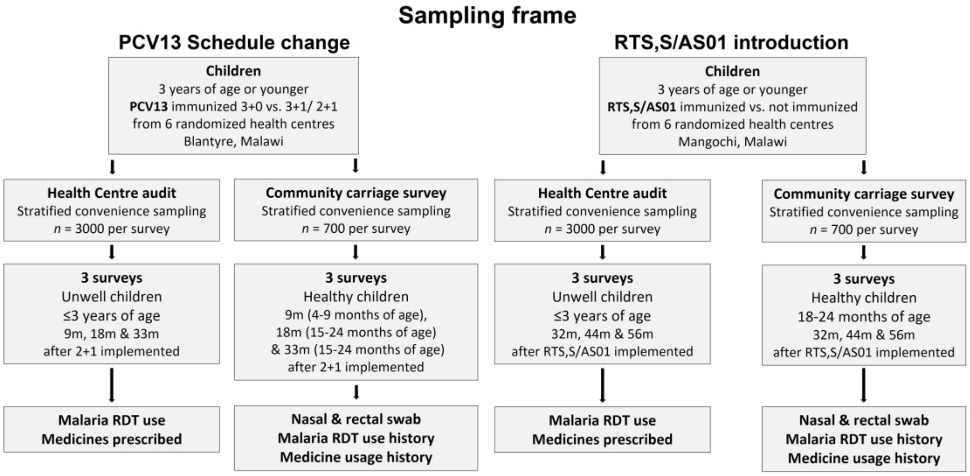

IVAR study sampling frame. PCV = pneumococcal conjugate vaccine, RTS,S/AS01 = malaria vaccine, m=months, RDT = rapid diagnostic test.

355x175mm (300 x 300 DPI)

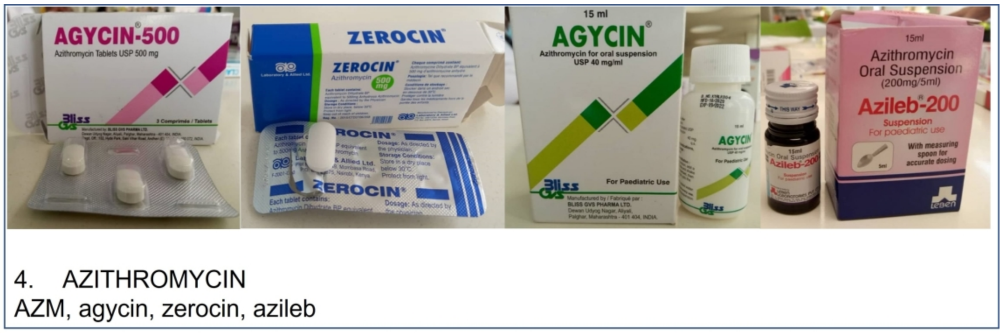

Example of an antibiotic picture (azithromycin) which will be used in the drug bag exercise as part of the community carriage survey. Method adapted from Dixon et al. (2019)[16].

262x87mm (300 x 300 DPI)

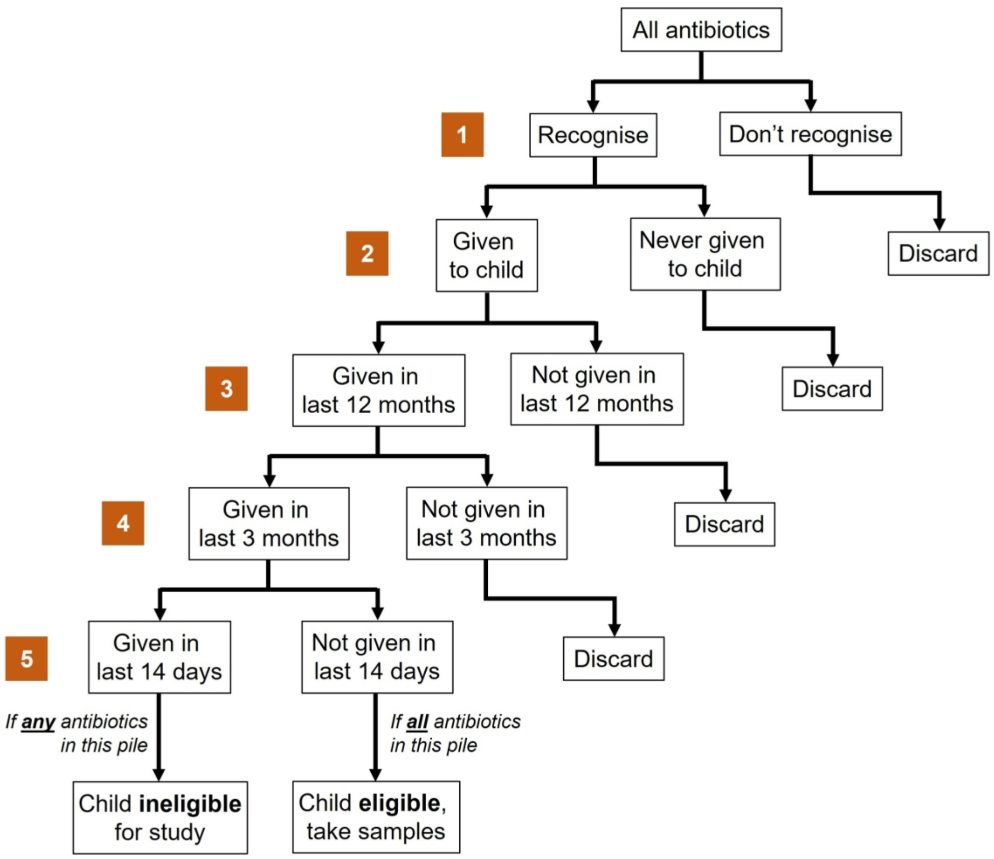

Flow diagram of questionnaire workflow of the 'drug bag' component of the community carriage survey. Each orange number corresponds to a round of questioning, starting with all locally identified, available antibiotics being presented to participant's parents/caregivers. The pool of antibiotics available for review is expected to diminish in each successive round, with the final round (round 5) being included as a final check that no child has been given antibiotics within the 14 days prior to sampling, which would render them ineligible for survey participation.

178x152mm (300 x 300 DPI)

BMJ Open

Cross-sectional health centre and community based evaluation of the impact of pneumococcal and malaria vaccination on antibiotic prescription and usage, febrile illness and antimicrobial resistance in young children in Malawi: the IVAR study protocol

Journal:	*BMJ Open*
Manuscript ID	bmjopen-2022-069560.R1
Article Type:	Protocol
Date Submitted by the Author:	03-Apr-2023
Complete List of Authors:	Singleton, David; University of Liverpool<br>Ibarz-Pavon, Ana; University of Liverpool; Kamuzu University of Health Sciences, Malawi-Liverpool-Wellcome Trust Clinical Research Programme<br>Swarthout, Todd; UCL, Division of Infection and Immunity; University Medical Centre Utrecht, Julius Center for Health Sciences and Primary Care<br>Bonomali, Farouck; Malawi-Liverpool-Wellcome Trust Clinical Research Programme, Kamuzu University of Health Sciences<br>Cornick, Jennifer; University of Liverpool; Malawi-Liverpool-Wellcome Trust Clinical Research Programme<br>Kalizang'oma, Akuzike; Kamuzu University of Health Sciences, Malawi-Liverpool-Wellcome Trust Clinical Research Programme; University College London, Research Department of Infection<br>Ntiza, Noah; Kamuzu University of Health Sciences, Malawi-Liverpool-Wellcome Trust Clinical Research Programme<br>Brown, Comfort; Kamuzu University of Health Sciences, Malawi-Liverpool-Wellcome Trust Clinical Research Programme<br>Chipatala, Raphael; Kamuzu University of Health Sciences, Department of Public Health<br>Nyangulu, Wongani; Kamuzu University of Health Sciences, Department of Public Health<br>Chirombo, James; Kamuzu University of Health Sciences, Malawi-Liverpool-Wellcome Trust Clinical Research Programme<br>Kawalazira, Gift; Ministry of Health<br>Chibowa, Henry; Mangochi District Council<br>Mwansambo, Charles; Ministry of Health<br>Maleta, Kenneth ; Kamuzu University of Health Sciences, Department of Public Health<br>French, Neil; University of Liverpool, Department of Clinical Infection, Microbiology & Immunology, Institute of Infection and Global Health<br>Heyderman, Robert; UCL, Division of Infection and Immunity
**Primary Subject Heading**:	Epidemiology
Secondary Subject Heading:	Global health, Infectious diseases, Paediatrics, Research methods

Keywords:	BACTERIOLOGY, EPIDEMIOLOGY, Clinical audit < HEALTH SERVICES ADMINISTRATION & MANAGEMENT, Protocols & guidelines < HEALTH SERVICES ADMINISTRATION & MANAGEMENT, IMMUNOLOGY, INFECTIOUS DISEASES

SCHOLARONE™ Manuscripts

Cross-sectional health centre and community based evaluation of the impact of pneumococcal and malaria vaccination on antibiotic prescription and usage, febrile illness and antimicrobial resistance in young children in Malawi: the IVAR study protocol

David A. Singleton[1*#], Ana Ibarz-Pavon[1,2*], Todd D. Swarthout[2,3,4], Farouck Bonomali[2], Jennifer E. Cornick[1,2], Akuzike Kalizang'oma[2,3], Noah Ntiza[2], Comfort Brown[2], Raphael Chipatala[5], Wongani Nyangulu[5], James Chirombo[2], Gift Kawalazira[6], Henry Chibowa[7], Charles Mwansambo[8], Kenneth Maleta[5], Neil French[1¥*], Robert S. Heyderman[3¥]

Affiliations

[1] Institute of Infection, Veterinary, and Ecological Science, University of Liverpool, Liverpool, United Kingdom

[2] Malawi-Liverpool-Wellcome Research Programme, Kamuzu University of Health Sciences, Blantyre, Malawi

[3] Research Department of Infection, Division of Infection and Immunity, University College London, London, United Kingdom

[4] Julius Center for Health Sciences and Primary Care, Dept Epidemiology, University Medical Centre Utrecht, Utrecht, Netherlands

[5] Department of Public Health, School of Public Health & Family Medicine, Kamuzu University of Health Sciences, Blantyre, Malawi

[6] Blantyre District Health Office, Blantyre District Council, Blantyre, Malawi

[7] Mangochi District Health Office, Mangochi District Council, Mangochi, Malawi

[8] Malawi Ministry of Health, Lilongwe, Malawi

[*] David A. Singleton and Ana Ibarz-Pavon contributed equally to this manuscript

[¥] Neil French and Robert Heyderman contributed equally to this manuscript

[#] Correspondence to:

David A. Singleton

University of Liverpool, 8 West Derby Street, Liverpool L69 7BE, UK

Email: D.A.Singleton@liverpool.ac.uk

For peer review only - http://bmjopen.bmj.com/site/about/guidelines.xhtml

Abstract

Introduction

Vaccination is a potentially critical component of efforts to arrest development and dissemination of antimicrobial resistance, though little is known about vaccination impact within low-and-middle-income countries. This study will evaluate the impact of vaccination on reducing carriage prevalence of resistant *Streptococcus pneumoniae* and extended spectrum beta-lactamase-producing *Escherichia coli* and *Klebsiella* species. We will leverage two large ongoing cluster-randomised vaccine evaluations in Malawi assessing; firstly, adding a booster dose to the 13-valent pneumococcal conjugate vaccine (PCV13) schedule, and secondly, introduction of the RTS,S/AS01 malaria vaccine.

Methods and analysis

Six cross-sectional surveys will be implemented within primary healthcare centres (*n*=3,000 users of outpatient facilities) and their local communities (*n*=700 healthy children per survey): three surveys in Blantyre District (PCV13 component) and three surveys in Mangochi District (RTS,S/AS01 component). We will evaluate antibiotic prescription practices and AMR carriage in children ≤3 years. For the PCV13 component, surveys will be conducted 9, 18 and 33 months following a 3+0 to 2+1 schedule change. For the RTS,S/AS01 component, surveys will be conducted 32, 44 and 56 months post-RTS,S/AS01 introduction. Six health centres in each study component will be randomly selected for study inclusion. Between intervention arms, the primary outcome will be the difference in penicillin non-susceptibility prevalence amongst *Streptococcus pneumoniae* nasopharyngeal carriage isolates in healthy children. The study is powered to detect an absolute change of 13 percentage points (*i.e.,* 35% vs 22% penicillin non-susceptibility).

Ethics and dissemination

This study has been approved by the Kamuzu University of Health Sciences (Ref: P01-21-3249), University College London (Ref: 18331/002) and University of Liverpool (Ref: 9908) Research Ethics Committees. Parental/caregiver verbal or written informed consent will be obtained prior to inclusion or recruitment in the health centre- and community-based activities, respectively. Results will be disseminated via the Malawi Ministry of Health, WHO, peer-reviewed publications and conference presentations.

For peer review only - http://bmjopen.bmj.com/site/about/guidelines.xhtml

For peer review only

Word count: 5116

Keywords: *Streptococcus pneumoniae*; malaria; 13-valent pneumococcal conjugate vaccine; PCV13; Malawi; Africa; children; pneumococcal conjugate vaccines; malaria vaccine; RST,S/AS01; antimicrobial resistance; antibiotic prescription; antibiotic exposure; antibiotic access

Strengths and limitations of this study

- The study builds upon two large-scale vaccine evaluations, leveraging the infrastructure, methodology, and community engagement developed by such evaluations.
- The study expands upon a range of studies in Malawi evaluating antibiotic exposure and development of antibiotic resistant pathogen carriage, while also developing new methods utilising established methods as comparators.
- The study will enable methodologies to be evaluated against two vaccine delivery scenarios: (1) adaptation of delivery schedule of a pre-existing vaccine (PCV13), and (2) introduction of a new vaccine (RTS,S/AS01).
- Though our study design may limit representativeness, we have opted for a largely pragmatic design due to operational challenges in this setting.
- Despite monitoring throughout the study, there is nevertheless a risk of contamination between intervention and non-intervention arms (*i.e.,* children receiving an RTS,S/AS01 vaccine who relocate to a zone where RTS,S/AS01 is not being introduced, or vice-versa).

Introduction

Antimicrobial resistance (AMR) is a leading global health threat [1], with 1.27 million deaths being attributable to AMR in 2019 alone [2]. AMR development is thought to be primarily driven by antimicrobial exposure [3], but with resistant genes and their host bacteria capable of passing between people, animals, and the environment [4] multi-faceted approaches are needed if we are to curb AMR development and dissemination [4]. In 2016 a global review on

For peer review only - http://bmjopen.bmj.com/site/about/guidelines.xhtml

AMR set out ten recommendations for tackling this global pandemic [5], one of which was vaccination [5,6].

Vaccines may directly and indirectly impact on AMR [7]. Directly, vaccines target bacterial species with emerging clinical resistance issues (*e.g., Streptococcus pneumoniae, Salmonella typhi*) [8–10]. However, vaccines that target viruses and parasites may also deliver an indirect effect on AMR [7,11]. This may be *via* (i) removal of a 'gateway' pathogen for bacterial infection, (ii) improving general health, or (iii) reducing frequency of symptoms commonly associated with antibiotic prescription (*e.g.,* fever) [12], thereby reducing selection pressure for AMR development [7,11]. Conversely, vaccines may also exert a resistance selection pressure on target and/or 'bystander' pathogens [13,14]. Thus, for us to understand these complex interactions more fully, it is crucial that the putative impact of vaccines on AMR is evaluated in a systematic manner.

Varying responses to vaccination have been observed between High Income (HIC) and Low-and-Middle-Income Countries (LMICs), with vaccines frequently under-performing expectations in LMICs despite high vaccine coverage rates [15]. However, many LMICs also have severely limited access to diagnostics and appropriate antibiotics [16], with empirical and potentially unnecessary antibiotic prescriptions being a common reality of clinical practice [17]. Hence, whilst there is an intrinsic need to conduct vaccine impact evaluations in both HICs and LMICs, there is also a need to evaluate whether vaccination can play a cost effective [18] role in assisting equitable provision of antibiotics to those at greatest need.

Pneumococcal conjugate vaccines (PCVs) have arguably received the most attention of any vaccine regarding their potential to reduce AMR [7]. In the USA and the UK, the introduction of 7-valent (PCV7) and, later, 13-valent (PCV13) vaccines were associated with considerable reductions in resistant pneumococcal infections [19–21]. Malawi introduced PCV13 in November 2011 using a 3+0 delivery schedule (1 dose at 6, 10 and 14 weeks of age), with vaccine coverage exceeding 90% [22]. Though introduction was associated with significant reductions in invasive pneumococcal disease [23] and all-cause mortality [24], vaccine serotype carriage has remained persistently high [25], and penicillin non-susceptibility in both carriage [15,26] and disease samples [27] has not decreased, particularly in non-vaccine

For peer review only - http://bmjopen.bmj.com/site/about/guidelines.xhtml

serotypes [15]. Hence, some have queried whether PCV13 delivery can be further optimised [28]. The two PCV13 delivery schedules recommended by WHO include a 3+0 and a 2+1 (1 dose at 6 and 14 weeks of age and a booster at 9 months of age) schedule [29]. Given that some countries reporting reductions in AMR following PCV13 introduction utilise a booster dose [19–21], it is important to evaluate the role of a booster dose in reducing pneumococcal carriage, disease, antibiotic prescriptions and, ultimately, AMR. In 2021 a pragmatic, cluster-randomised evaluation on the impact on pneumococcal carriage of changing the existing 3+0 PCV13 delivery schedule to a 2+1 schedule was implemented in the Blantyre district of Malawi (known as the 'PAVE' study) [30]; our study will leverage the PAVE study to also assess the impact of delivery schedule change on AMR.

In 2021, RTS,S/AS01 became the first malaria vaccine to be recommended by the WHO for widespread use in young children [31]. A subunit vaccine targeting *Plasmodium falciparum* (*P. falciparum*), it is delivered via three doses at five, six and seven months of age followed by a fourth dose at 18-21 months of age [32]. Phase III trials indicated that the RTS,S/AS01 vaccine was effective at reducing clinical malaria [33]. However, although rare, increases in febrile convulsions, meningitis, cerebral malaria, and mortality rates in RTS,S/AS01 vaccinated individuals [34] led to a recommendation for further safety profiling and impact assessment [32]. Following this, the WHO announced phase IV evaluations in selected areas in Ghana, Kenya and Malawi (known as the 'EPI-MAL' study) [32]. We hypothesised that reductions in malaria-associated febrile illness in young children may be associated with reductions in antibiotic exposure and therefore AMR [12]. RTS,S/AS01 vaccination may also be associated with generalised improvements in health, reducing antibiotic exposure [7,11,12], and this study will work in conjunction with the ongoing phase IV evaluations in the Mangochi district of Malawi [32] to assess these hypotheses.

Hence, the "Impact of Vaccines on Antimicrobial Resistance" (IVAR) study aims to leverage the PAVE and EPI-MAL studies to evaluate the impact of (i) changing from a 3+0 to a 2+1 PCV13 delivery schedule, and (ii) introducing a novel non-bacterial vaccine (RTS,S/AS01) on antibiotic prescription, febrile illness, and AMR carriage in children ≤3 years of age in Malawi. The IVAR study will also deploy methods for measuring antibiotic prescription and exposure

For peer review only - http://bmjopen.bmj.com/site/about/guidelines.xhtml

in primary healthcare and community settings. As such, this study addresses three key research questions:

1) Can vaccination reduce the prevalence of antibiotic-resistant pneumococcal and extended spectrum beta-lactamase-producing (ESBL) *Escherichia coli (E. coli)* and *Klebsiella* species carriage in healthy children?
2) Can vaccination reduce incidence of febrile illness?
3) Can vaccination reduce incidence of antibiotic prescription?

Methods and analysis

Study setting

The IVAR study, in conjunction with the PAVE study [30] (ClinicalTrials.gov NCT04078997, implemented 16 March 2021), will evaluate the impact on AMR of changing the 3+0 to a 2+1 delivery schedule in children ≤3 years of age in the Blantyre District, southern Malawi (Figure 1) [30]. Blantyre District is a mixed urban and rural setting composed of 1.3 million residents over 1,800km^2, with children under the age of five comprising 16% of the population [35]. Throughout the district there are 28 governmental primary care Health Centres (HCs) [30] which serve defined catchment areas (Figure 2). The Malawi Ministry of Health and Blantyre District Health Office (DHO) randomly selected ten HCs to implement a WHO-approved 2+1 PCV13 delivery schedule (intervention arm), with ten other HCs randomly selected to continue the 3+0 schedule and serve as the comparator arm [30] ; the IVAR study will work with a random selection of these health centres.

The IVAR study, in collaboration with an ongoing RTS,S/AS01 phase IV evaluation (ClinicalTrials.gov NCT03806465; RTS,S/AS01 introduced April 2019), will also work to evaluate the impact of RTS,S/AS01 on AMR in Mangochi District, southern Malawi (Figure 1). Mangochi District is predominantly rural and is composed of 1.1 million residents over 6,700km^2, with children under the age of five comprising 18% of the population [35]. Throughout the district there are 29 governmental primary care HCs [36] which serve defined catchment areas (Figure 3). Within this setting, RTS,S/AS01-exposed (*n*=5 HCs) and non-exposed (*n*=3 HCs) clusters have already been selected as part of a separate RTS,S/AS01 introduction evaluation [32]; the IVAR study will work with a random selection of these health centres. Vaccine-exposed HCs were geographically adjacent to each other (*i.e.,* exposed HCs

For peer review only

For peer review only - http://bmjopen.bmj.com/site/about/guidelines.xhtml

are in Mangochi township itself or within <13 miles of the town, whereas non-exposed HCs are 14-38 miles outside of Mangochi township) [32].

Study site selection

For the PCV13 component of this study, we will randomly select six HCs for IVAR study inclusion, including *n*=3 HCs switching to 2+1 and *n*=3 HCs continuing to provide 3+0, stratified by setting (urban, peri-urban and rural HCs) (Figure 2). These will be selected from the ten 2+1 and ten 3+0 HCs that have already been selected for inclusion in the aforementioned PCV13 schedule change evaluation. Of note, a 3+1 PCV13 schedule was implemented amongst a subset of children following the schedule change, targeting children living in the catchment area of a 2+1 HC and who had received their first or second PCV13 primary doses prior to the HC implementing the schedule change [30]. These children will be eligible for recruitment.

For the RTS,S/AS01 component of this study, we will randomly select six HCs for IVAR study inclusion (*n*=3 RTS,S/AS01-exposed and *n*=3 non-exposed HCs) from the five RTS,S/AS01 exposed and three non-exposed HCs [32], stratified by setting (urban, peri-urban and rural HCs) (Figure 3).

Study design

There will be six HC- and community-based cross-sectional surveys, split between the PCV13 (*n*=3 surveys) and the RTS,S/AS01 component (*n*=3 surveys) evaluations. For the PCV13 component, surveys will be conducted 9-, 18- and 33-months post-schedule change implementation. For the RTS,S/AS01 component, surveys will be conducted 32, 44 and 56 months post-introduction. The surveys will be composed of two concurrent data collection activities. Firstly, a community-based carriage survey of healthy children will be implemented, with collection of biological samples (nasopharyngeal and rectal swabs) and collection of information pertaining to the history of the child's febrile illness, malaria and antibiotic prescription history. Secondly, we will implement an anonymised audit of malaria rapid diagnostic test (mRDT) use and medicinal prescriptions (with a focus on antibiotics) in children presenting unwell to the outpatient department (OPD) of HCs, as recorded within each child's Health Passport (HP). Whilst for all surveys the HC audit will summarise children ≤3 years of

For peer review only - http://bmjopen.bmj.com/site/about/guidelines.xhtml

age, the age-based eligibility of the community carriage survey will vary according to survey and vaccine evaluation; a study sampling frame is included in Figure 4. Data collection for this study was initiated in December 2021 and is scheduled to complete in March 2024.

Primary objectives

The primary objective is to evaluate the reduction in carriage prevalence of penicillin resistant *S. pneumoniae* (see section 2c for further details), ESBL *E.coli* and *Klebsiella* species following a PCV13 schedule change (Blantyre District) and, separately, following RTS,S/AS01 vaccine introduction (Mangochi District). This will answer the question of whether vaccines can play a role in reducing the carriage prevalence of resistant pathogens in young children in LMICs.

Secondary objectives

The secondary objectives are to evaluate (a) incidence of febrile illness and antibiotic prescription and exposure (thus providing a mechanism for any reduction in AMR), (b) incidence of macrolide, tetracycline and trimethoprim-sulfamethoxazole non-susceptibility in *S. pneumoniae,* and (c) changes to the wider upper respiratory tract and gastrointestinal resistome variation following PCV13 schedule change and RTS,S/AS01 vaccine introduction.

Data collection activities

(1) Health centre audit

Data will be collected on vaccine cluster designation (*i.e.,* receiving intervention or not), vaccine schedule compliance, medicines prescribed, and febrile illness presentation (using mRDT use as a proxy) during that visit via review of the child's HP. This audit is intended to assess the impact of PCV13 schedule change or RTS,S/AS01 introduction on mRDT use and antibiotic prescription frequency.

a) Population and sampling

The audit will include children ≤3 years of age presenting to the OPD of selected HCs. Each HC will be assessed over at least two weeks during each survey; HCs in opposing study arms *(i.e.,* 2+1 *vs*. 3+0; RTS,S/AS01 *vs.* no RTS,S/AS01) will be paired according to setting and will be surveyed concurrently or on adjacent weeks depending on staff availability and local conditions (including weather patterns and related accessibility). Children's HPs will be

For peer review only - http://bmjopen.bmj.com/site/about/guidelines.xhtml

reviewed upon HC exit. Study teams will aim to review the HP of every eligible child; however, to avoid disrupting HC workflow, during busy periods study teams will not request that potentially eligible children wait until a member of the study team is available to review their HP.

b) Inclusion and exclusion criteria

Inclusion criteria will be: (1) ≤3 years of age, (2) HC OPD attendance for investigation and/or treatment of ill health, (3) verbal consent has been provided by parent/caregiver for HP review, and (4) HP available for review. Exclusion criteria will be: (1) HC attendance for vaccination, and (2) HC attendance for routine health examination (*e.g.,* weighing) and found to be well. As HPs are reviewed anonymously and HPs are reviewed on a per visit basis, children may be recorded more than once within each survey.

c) Intervention

Children's HPs will be reviewed upon children exiting selected HCs, having presented to the OPD.

d) Expected outcomes

The primary outcome will be the difference in antibiotic prescription incidence between intervention arms, measured as a proportion of total OPD visits of children ≤3 years of age within each surveyed period. A secondary outcome will be difference in mRDT use, again as a proportion of total OPD visits. In this study, we use mRDT as a proxy for febrile illness. Additional outcomes include sub-analyses by specific antibiotic agent/class. Depending on availability of current census data, analyses may also encompass overall OPD visit incidence relative to paediatric populations within each health centre's catchment area.

e) Study power and sample size calculation

A previous HC OPD review estimated that 70% of children under five years of age were prescribed antibiotics (Priyanka Patel, MLW, personal communication). Hence, the primary outcome is powered to detect a difference of 5% in antibiotic prescription incidence between intervention groups (65% *vs*. 70%). Two-tailed sample sizes were calculated setting confidence at 95% and power at 80%, indicating that 1,417 HPs would be required in each

For peer review only

For peer review only - http://bmjopen.bmj.com/site/about/guidelines.xhtml

study arm. Hence, we will review 3,000 HPs per survey (*n*=18,000 for total study). HC-level sample sizes will be weighted according to estimated or actual population sizes within their respective catchment areas [32].

f) Informed consent process

Due to an anticipated high participation rate, we have developed a method, approved by the relevant Research Ethic Committees, to maximise efficiency in attaining verbal consent and capturing the needed information. Parents/caregivers will receive verbal (and written if they wish) information about the study activity, and will have the opportunity to ask questions and express their doubts and concerns before accepting to take part. Verbal parent/caregiver consent will then be provided voluntarily if they choose to participate.

g) Data collection, management and anonymisation procedures

Data from a questionnaire will be collected by password-protected electronic data capture (Supplementary file 1). Participation will be completely anonymised, with no personal data collected. Data will be uploaded daily to a secured on-site server, which is backed up daily to both local and off-site facilities.

h) Statistical analyses

Only categorical variables will be collected; these will be defined by frequency distributions. Descriptive analyses encompassing both outcomes and vaccination status will be performed. Mixed effects logistic regression models investigating presence of (1) antibiotic prescription and (2) mRDT use on the day of HC attendance in individual children as outcome variables will be implemented. HC will be modelled as a random effect, with findings being balanced against the opposing outcome variable; vaccination status; other medicinal prescriptions; HC setting, and month of HC visit. Individual antibiotic classes/agents may also be explored as sub-analyses. Data collected here will be anonymously compared against routine attendance registry data collected by selected HCs.

(2) Community carriage survey

The community carriage surveys will focus on healthy children (Figure 4). Nasopharyngeal and rectal swab samples will be collected in addition to demographic data, vaccine compliance,

For peer review only - http://bmjopen.bmj.com/site/about/guidelines.xhtml

febrile illness, history of mRDT use and medicine prescription and exposure history. These data will be informed via direct parent/caregiver questioning, review of the child's HP, and an antibiotic provision recall exercise known as the 'drug bag method' [16].

a) Population and sampling

A stratified convenience sampling approach will be applied, making use of available local census data and utilising networks of Health Surveillance Assistants (HSAs) and health volunteers when needed. The sampling approach is intended to maximise efficiency in recruiting children who have received the appropriate intervention for the HC catchment area in which they reside. Hence, sampling will identify villages that are most proximal to HCs and will work to recruit all eligible children within those villages. Additional villages will be approached if needed until sampling targets are met.

i. PCV13 component

Children aged 4-9 months will be sampled in the first survey to establish the baseline difference between children provided with three (3+0 schedule) or two (2+1 schedule) primary PCV13 doses. Children residing in 2+1 catchment areas and who have received 3 primary PCV13 doses (referred to as '3+1 children') will be eligible for study inclusion. In the second and third surveys, children 15-24 months of age will be sampled to compare children who have received a booster dose (2+1) against those who have not (3+0).

There is limited census data available in these settings. Thus, we will work closely with local HSAs and health volunteers who will assist with development of community engagement strategies (including communication with community leaders), identify eligible children, locate targeted households, and facilitate communication with household members.

ii. RTS,S/AS01 component

Children aged 18-24 months of age will be sampled across all three surveys to compare children who have received the RTS,S/AS01 vaccine to unvaccinated children. Recent censuses have been completed to support the RTS,S/AS01 evaluation[32], providing greater confidence in local population estimates. Hence, parents/caregivers of potentially eligible children will be located and contacted directly by study teams.

For peer review only - http://bmjopen.bmj.com/site/about/guidelines.xhtml

b) Inclusion and exclusion criteria

For both the PCV13 and RTS,S/AS01 components, inclusion criteria includes (1) Age of child within the range determined by the particular survey (Figure 4), (2) permanent residence in the relevant study site, (3) parent/caregiver providing written informed consent, (4) evidence of having received a full initial (primary dose/s) vaccine course particular to evaluation and study arm, and (5) that the child is healthy at time of sampling.

Exclusion criteria includes (1) child having received antibiotics within the previous 14 days, (2) child currently receiving tuberculosis treatment, (3) child having been hospitalised for pneumonia within the previous 14 days, (4) presence of gross respiratory pathology, (5) child having a terminal illness, (6) child previously recruited into the current survey, and (7) parent/caregiver not providing informed written consent.

For the second and third surveys of the PCV13 component, children residing in 2+1 clusters must have received the booster PCV13 dose. For the RTS,S/AS01 component, receipt of the booster dose is not a requirement in all three surveys, though provision will be recorded. Children may only be included in each survey once; however, if they fulfil the inclusion criteria they may be enrolled in subsequent surveys.

c) Sample collection & laboratory processing

For both the PCV13 and RTS,S/AS01 components, sample collection will include nasopharyngeal and rectal swabs from each participant. Following previously described WHO recommendations [25,37], nasopharyngeal swabs will be collected in skim milk-tryptone-glucose-glycerine (STGG) medium and rectal swabs will be collected in Cary-Blair medium, both being stored in -80°C freezers at the Malawi-Liverpool-Wellcome Research Programme (MLW) laboratory in Blantyre within 10 hours of collection for later batch-testing. Samples collected in Mangochi District will initially be taken to the Public Health and Nutrition Research Group (PHNG) laboratory, collocated to the Mangochi District Hospital, for storage prior to transport to MLW. Samples will be cultured to isolate and characterize *S. pneumoniae*, ESBL *E. coli* and *Klebsiella* species at MLW. Isolates will then be sent to the United Kingdom for Whole Genome Sequencing (WGS).

For peer review only - http://bmjopen.bmj.com/site/about/guidelines.xhtml

For *S. pneumoniae,* penicillin non-susceptibility will be defined genotypically, as a minimum inhibitory concentration (MIC) over 0.06 ml/L; 200 genotypically non-susceptible isolates will be phenotypically tested to confirm genotypic findings. Non-susceptibility to macrolides (azithromycin, MIC 0.25< mg/L), tetracyclines (doxycycline, MIC 1.00< mg/ML) and trimethoprim-sulfamethoxazole (MIC 1.00< mg/ML) will also be examined genotypically with phenotypic confirmation as secondary objectives. Sub-analyses will include determining serotype of *S. pneumoniae isolates.* 800 carriage samples will be selected for broader resistome analyses.

E. coli and *Klebsiella* species ESBL status will be determined phenotypically via chromogenic ESBL media. Depending on resource availability, a proportion of isolates will also undergo WGS and broader resistome analyses.

d) Data collection

A range of demographic, mRDT use and medicine exposure history will be collected from the participants' health passport. These data will be supplemented with direct parent/caregiver questioning. The drug bag method will also be used; this has previously been deployed in Malawi [16] and we will re-purpose this approach to assist parent/caregiver recall in identifying the different antibiotics given to the participating child. In brief, prior to study initiation the study team will obtain antibiotics routinely used for systemic administration (including multiple formulations of the same agent where obtainable) and available for dispensing or sale in the community (including HCs, hospitals, private pharmacies and informal sources). Parents/caregivers will be asked if they recognise individual antibiotics (presented as pictures, example in Figure 5). For recognised antibiotics, parents/caregivers will be asked whether they have ever given the antibiotic to the participating child. If yes, they will be asked whether the antibiotic was given in the 12 months, 3 months or 14 days prior to recruitment (Figure 6). Findings from direct questioning, HP review and the drug bag exercise will be compared and combined to provide a more complete history of a participant's antibiotic exposure.

For peer review only

For peer review only - http://bmjopen.bmj.com/site/about/guidelines.xhtml

Study questionnaires are available in Supplementary file 2 (PCV13 component, survey 1), Supplementary file 3 (PCV13 component, surveys 2 and 3), and Supplementary file 4 (RTS,S/AS01 component, all surveys).

e) Primary and secondary outcomes

Carriage isolates: The primary outcome will be the difference in prevalence of penicillin non-susceptibility among *S. pneumoniae* carriage isolates, comparing i) the 2+1 vs. 3+0 intervention arms of the PCV13 component) and ii) the RTS,S/AS01 vs. no-RTS,S/AS01 intervention arms of the RTS,S/AS01 component. Secondary outcomes will also encompass macrolide, tetracycline and trimethoprim-sulfamethoxazole non-susceptibility.

Rectal swabs: The primary outcome will be the difference in prevalence of ESBL *E. coli* and *Klebsiella* species carriage comparing i) the 2+1 vs. 3+0 arms of the PCV13 component) and ii) the RTS,S/AS01 vs. no-RTS,S/AS01 arms of the RTS,S/AS01 component.

Antibiotic usage: The primary outcome will be the difference in incidence of antibiotic prescription intended for systemic administration from 14 days to three months prior to recruitment, comparing i) the 2+1 vs. 3+0 arms of the PCV13 component) and ii) the RTS,S/AS01 vs. no-RTS,S/AS01 arms of the RTS,S/AS01 component. Additional outcomes will include sub-analyses by specific antibiotic agent/ class and length of treatment.

Febrile illness: A secondary outcome will be the difference in incidence of febrile illness from 14 days to three months prior to recruitment, comparing i) the 2+1 vs. 3+0 arms of the PCV13 component) and ii) the RTS,S/AS01 vs. no-RTS,S/AS01 arms of the RTS,S/AS01 component. Additional secondary outcomes will include sub-analyses of mRDT use and results (including multiple positive tests per individual) and antimalarial treatment, comparing respective study arms.

f) Study power and sample size calculation

Previous surveys have indicated a minimum pneumococcus carriage prevalence of 60% in children under the age of five [25], with 35% of such isolates expected to display penicillin

For peer review only

non-susceptibility (unpublished data). Hence, the primary outcome is powered to detect a crude absolute decrease of 13 percentage points (13%) in penicillin non-susceptibility (*i.e.,* 35% *vs*. 22%). Two-tailed sample sizes were calculated setting confidence at 95% and power at 80%, indicating that 204 pneumococcus isolates would be required in each study arm per survey. Hence, allowing for a 60% carriage prevalence, 700 samples will be collected per component per survey (*n*=4,200 for total study). HC-level sample sizes will be weighted according to estimated or actual population sizes within their respective catchment areas [32].

A previous survey has indicated a minimum ESBL *E. coli* carriage of 25% in Blantyre (Nicholas Feasey, MLW, personal communication). Hence, the sample size per survey noted above will allow a crude absolute difference of 8 percentage points (8%) to be detected (*i.e.,* 25% *vs*. 17%) between study arms.

g) Informed consent process

Participant's parents/caregivers will receive written and verbal information about the study, and will have the opportunity to ask questions, express their doubts and concerns, and have time to reflect before deciding to take part or not. An informed consent form will be signed and dated by the participant's parent/caregiver and a member of the research team, a copy of which will be retained by the parent/caregiver. Participant's parents/caregivers may withdraw consent at any point without need to provide a reason, and without penalty.

h) Data collection, management and anonymisation procedures

Data will be collected using password-protected electronic data capturing. Each participant will be assigned a unique participant identification number (PID) at recruitment. This PID will be used in all datasheets and files, and will be linked to laboratory data, thus, only anonymised data will be used for analyses. A logbook containing identifiable information (including name) will be kept separately in a secured location and will only be accessed by authorised study team members. This will allow the study team to recover any missing epidemiological information, if necessary, later (*e.g.,* missing vaccination dates), and to facilitate any participant's parents/caregivers who wish to withdraw consent.

For peer review only

For peer review only - http://bmjopen.bmj.com/site/about/guidelines.xhtml

i) Statistical analyses

Continuous variables will be expressed as means and standard deviations, or medians and interquartile ranges. Categorical variables will be defined by frequency distributions. Descriptive analyses encompassing primary, secondary and additional outcomes, and vaccination status, will be performed. Mixed effects logistic regression models investigating prevalence of penicillin non-susceptible *S. pneumoniae* across all pneumococcus carriage isolates as an outcome variable will form the primary analysis. HC will be modelled as a random effect, with findings being balanced against key demographic variables (*i.e.,* age, sex etc.), vaccination status, antibiotic and febrile illness history, rurality, and the month in which sampling was completed. This approach will be repeated for other antibiotic classes, and for ESBL *E. coli* and *Klebsiella* species. We will also descriptively analyse antibiotic prescription and febrile illness history (using mRDT usage as a proxy), and may progress to inferential statistics if justified.

For peer review only

Patient and public involvement

Prior to development of the protocol, key stakeholders were informed of the study, including the selected HCs and their surrounding communities, the District Health Office, Ministry of Health and the Ministry of Education. We actively sought and incorporated input from these stakeholders into the study objectives and overall design. Given that this study collaborates closely with two existing vaccine evaluations, pre-existing community engagement and sensitisation will strengthen community trust at the onset of this study. Especially in light of COVID-19, we envisage expanded community engagement prior to start of data collection, encompassing HCs, community leaders, HSAs, health volunteers, and members of the communities themselves in information-providing activities. We will repeat community engagement activities prior to each survey, and provide feedback on prior surveys where possible.

Ethics and dissemination

Ethical approval

This study has been approved by the Research Ethic Committees (REC) of Kamuzu University of Health Sciences (Ref: P01-21-3249), University College London (Ref: 18331/002) and the University of Liverpool (Ref: 9908) Research Ethics Committees. Parental/caregiver verbal

For peer review only - http://bmjopen.bmj.com/site/about/guidelines.xhtml

(health centre audit) or written (household carriage survey) informed consent will be obtained prior to inclusion or participation, as described earlier.

Dissemination policy and plans

Study results will be shared with local communities and stakeholders, the Malawi Ministry of Health, other relevant policymakers and decision-making stakeholders, and published in peer-reviewed journals. Findings will be presented at international conferences and meetings. Copies of all published materials and reports will be shared with the research ethics committees and collaborators. Procedures for strain exchange, data sharing and ownership will follow Nagoya protocol standards [38].

Discussion

To date, attempts to define an impact of vaccination on antimicrobial exposure and resistance patterns have largely been restricted to randomised controlled trials (RCTs) and post-authorisation retrospective analyses [7,39,40], generally within high income countries [39]. However, of note, Lewnard *et al.* (2020) utilised LMIC household survey data collected 2006-2018 to demonstrate significant reductions in antibiotic exposure associated with introduction of childhood pneumococcal and rotavirus vaccines [41]. Whilst promising, such impacts may be reduced in the longer term, particularly in high carriage prevalence settings [42] or via serotype replacement [43]. Indeed, following PCV13 introduction in Malawi, high residual pneumococcal carriage has been observed eight years post-PCV13 introduction [25], along with emergence of resistant serotypes [15]. Thus, there is a need to conduct a thorough evaluation in a high carriage, low-income setting.

Here we have leveraged two existing evaluations [30,32], seeking to define impact following vaccine intervention in Malawi. Though this has enabled more efficient study preparation, it does mean that we are dependent on existing evaluation methodologies. For example, both evaluations utilise a cluster randomised approach. This is a reasonable approach to take in a country where population censuses are infrequent and where individual, blinded randomisation would prove impractical to deliver within health centres. However, cluster randomisation does carry a risk of contamination between clusters [44]. To minimise this risk, we have opted to stratify our clusters into zones proximate to and more distant from health

For peer review only - http://bmjopen.bmj.com/site/about/guidelines.xhtml

centres, and only sample from the health centre proximate zones. Nonetheless, we do acknowledge this more limited sampling frame may limit representativeness.

It must be remembered that primary care in Malawi is a system under stress [17]. Although health centre-level records are utilised, these might be in electronic or paper-based forms, the latter being vulnerable to illegibility, damage and loss [45]. Thus, to further understand primary care antibiotic prescribing, a robust study method is needed which minimises disruption for already over-stretched health centre staff, whilst also enabling rapid informed consenting and data collection. For this reason, we sought to establish and optimise an ethical approach of verbally consented rapid health passport review upon exiting health centres.

Though not yet formally quantified, antibiotics are frequently informally (e.g. private pharmacies and the local market) acquired in Malawi [46]. Thus, patient-held health records likely only represent a partial picture of a patient's disease history and antibiotic exposure [16,45]. For the community-based component of this study, we will implement visual recall methods previously utilised in this setting [16]. However, it should be remembered that we will remain reliant on participant recall. Similarly, while incidence of malaria would be the optimal endpoint, due to uncertainties surrounding access to diagnostic services, we are using febrile illness as a proxy. However, where recorded, we will also consider mRDT use and findings. To assist with this aim for both antibiotic exposure and febrile illness, we hope to also gain access to patient-held health records. This will enable comparisons to be made which will at least partially negate these limitations.

Considering further limitations, due to resource constraints the surveys will be cross-sectional, meaning that we will not be able to gain detailed understanding of seasonal variation in prescribing practices nor AMR. We will be able to manually summarise longitudinal prescription and mRDT use from largely paper-based health centre-level health records; however, this will be resource intensive and does not represent a sustainable long-term approach. Finally, in the likely absence of reliable population data, we are reliant on using proportional outcome measures, though census data will be used where possible. It is possible that such proportional measures may mask wider variation between intervention arms e.g., highly positive vaccine effects leading to absolute reductions in disease incidence.

For peer review only - http://bmjopen.bmj.com/site/about/guidelines.xhtml

To conclude, we present a protocol for a robust, pragmatic evaluation of pneumococcal and malaria vaccine impact on antimicrobial exposure, febrile illness and AMR carriage in young children, which considers the structural challenges of conducting such studies in a low-income country. Limitations considered, we are confident that this will provide a blueprint for wider evaluations to be conducted in other age groups and countries.

*** ***

Data availability statement

Anonymised data, including bacterial genome sequencing data, used in analyses for reported findings of this study will be made publicly available. Human subject metadata will be shared primarily with scientific collaborators but, where appropriate and with the proper safeguards, made freely available to researchers with an established scientific record. To access human subject metadata, a formal project proposal would be made and reviewed by the investigators and the Kamuzu University of Health Sciences Research Ethics Committee. Following approval, data would be released subject to a signed data user agreement. Procedures for strain exchange, data sharing and ownership will follow Nagoya protocol standards.

Acknowledgements

The authors thank the MLW laboratory management team, led by Brigitte Denis and George Selemani. We also thank the MLW data management team, and would especially like to commemorate the memory of Clemens Masesa who led this team until his passing. We are further appreciative of the guidance and advice provided by Andrea Gori, Eleanor MacPherson, Priyanka Patel, Derek Cocker, and Nicholas Feasey. We would also like to thank a range of supporting team members at MLW and KUHeS, particularly Mernani Kaonga and our data collection teams, in addition to the many HC team members, HSAs, health volunteers, community leaders and community members who without their kind support and time this study would not be possible.

Contributors

For peer review only

For peer review only - http://bmjopen.bmj.com/site/about/guidelines.xhtml

RSH, NF, KM, TDS and JEC conceived the study. DAS, AI, RSH, NF, KM, TDS, JEC and AK designed the study, with contributions from FB, NN, CG, RC, AK and WN. AI, TDS, JEC, CB and DAS oversaw development of laboratory methods. DAS, AI, FB and JEC designed study documents. JC conducted community mapping exercises. GK, HC and CM provided public health oversight and facilitated access to study sites. DAS and AI jointly wrote the first draft. All authors have read and approved the final manuscript.

Competing interests

The authors declare that they have no competing interests.

Funding

This work was funded by the Wellcome Trust (Ref: 219900/Z/19/Z) to RSH. The MLW Research Programme is supported by a Strategic Award from the Wellcome Trust, UK (206545/Z/17/Z). RSH is a National Institute of Health Research (NIHR) Senior Investigator. The views expressed in this publication are those of the authors and not necessarily those of the NIHR or the UK Department of Health and Social Care.

References

1 WHO. 10 global health issues to track in 2021. https://www.who.int/news-room/spotlight/10-global-health-issues-to-track-in-2021 (accessed 28 Feb 2022).

2 Murray CJL, Ikuta KS, Sharara F, *et al.* Global burden of bacterial antimicrobial resistance in 2019: a systematic analysis. *Lancet* 2022;**399**:629–55. doi:10.1016/S0140-6736(21)02724-0

3 Cantón R, Bryan J. Global antimicrobial resistance: from surveillance to stewardship. Part 1: surveillance and risk factors for resistance. *Expert Rev Anti Infect Ther* 2012;**10**:1269-1271 3p. doi:10.1586/eri.12.120

4 Wernli D, Jørgensen PS, Parmley EJ, *et al.* Evidence for action: a One Health learning platform on interventions to tackle antimicrobial resistance. *Lancet Infect Dis* 2020;**20**:e307–11. doi:https://doi.org/10.1016/S1473-3099(20)30392-3

5 O'Neill J. Tackling drug-resistant infections globally: final report and recommendations. 2016.http://amr-review.org/home

6 WHO. Resolution 72·5–antimicrobial resistance.

For peer review only

For peer review only - http://bmjopen.bmj.com/site/about/guidelines.xhtml

2019.https://apps.who.int/gb/ebwha/pdf_files/WHA72/A72_R5-en.pdf (accessed 11 May 2022).

7 Micoli F, Bagnoli F, Rappuoli R, *et al.* The role of vaccines in combatting antimicrobial resistance. *Nat Rev Microbiol* 2021;**19**:287–302. doi:10.1038/s41579-020-00506-3

8 Patel PD, Patel P, Liang Y, *et al.* Safety and Efficacy of a Typhoid Conjugate Vaccine in Malawian Children. *N Engl J Med* 2021;**385**:1104–15. doi:10.1056/NEJMoa2035916

9 Britto CD, Wong VK, Dougan G, *et al.* A systematic review of antimicrobial resistance in Salmonella enterica serovar Typhi, the etiological agent of typhoid. *PLoS Negl Trop Dis* 2018;**12**:e0006779.https://doi.org/10.1371/journal.pntd.0006779

10 Watkins ER, Kalizang'Oma A, Gori A, *et al.* Factors affecting antimicrobial resistance in Streptococcus pneumoniae following vaccination introduction. *Trends Microbiol* Published Online First: 6 September 2022. doi:10.1016/j.tim.2022.06.001

11 Marc L, R. SG, P. KK, *et al.* How Can Vaccines Contribute to Solving the Antimicrobial Resistance Problem? *MBio* 2022;**7**:e00428-16. doi:10.1128/mBio.00428-16

12 Maze MJ, Bassat Q, Feasey NA, *et al.* The epidemiology of febrile illness in sub-Saharan Africa: implications for diagnosis and management. *Clin Microbiol Infect* 2018;**24**:808–14. doi:https://doi.org/10.1016/j.cmi.2018.02.011

13 Christine T, W. OS, H. GY, *et al.* Estimating the proportion of bystander selection for antibiotic resistance among potentially pathogenic bacterial flora. *Proc Natl Acad Sci* 2018;**115**:E11988–95. doi:10.1073/pnas.1810840115

14 Cassiolato AP, Almeida SCG, Andrade AL, *et al.* Expansion of the multidrug-resistant clonal complex 320 among invasive Streptococcus pneumoniae serotype 19A after the introduction of a ten-valent pneumococcal conjugate vaccine in Brazil. *PLoS One* 2018;**13**:e0208211.https://doi.org/10.1371/journal.pone.0208211

15 Gori A, Obolski U, Swarthout TD, *et al.* The metabolic, virulence and antimicrobial resistance profiles of colonizing Streptococcus pneumoniae shift after pneumococcal vaccine introduction in urban Malawi. *medRxiv* 2021;:2021.07.21.21260914. doi:10.1101/2021.07.21.21260914

16 Dixon J, MacPherson E, Manyau S, *et al.* The 'Drug Bag' method: lessons from anthropological studies of antibiotic use in Africa and South-East Asia. *Glob Health Action* 2019;**12**:1639388. doi:10.1080/16549716.2019.1639388

17 MacPherson EE, Reynolds J, Sanudi E, *et al.* Understanding antimicrobial resistance

For peer review only

through the lens of antibiotic vulnerabilities in primary health care in rural Malawi. *Glob Public Health* 2021;:1–17. doi:10.1080/17441692.2021.2015615

18 Sevilla JP, Bloom DE, Cadarette D, *et al.* Toward economic evaluation of the value of vaccines and other health technologies in addressing AMR. *Proc Natl Acad Sci* 2018;**115**:12911–9. doi:10.1073/pnas.1717161115

19 Kyaw MH, Lynfield R, Schaffner W, *et al.* Effect of Introduction of the Pneumococcal Conjugate Vaccine on Drug-Resistant Streptococcus pneumoniae. *N Engl J Med* 2006;**354**:1455–63. doi:10.1056/NEJMoa051642

20 Ladhani SN, Slack MPE, Andrews NJ, *et al.* Invasive pneumococcal disease after routine pneumococcal conjugate vaccination in children, England and Wales. *Emerg Infect Dis* 2013;**19**:61–8. doi:10.3201/eid1901.120741

21 Moore MR, Link-Gelles R, Schaffner W, *et al.* Effect of use of 13-valent pneumococcal conjugate vaccine in children on invasive pneumococcal disease in children and adults in the USA: analysis of multisite, population-based surveillance. *Lancet Infect Dis* 2015;**15**:301–9. doi:https://doi.org/10.1016/S1473-3099(14)71081-3

22 Tsega A, Hausi H, Chriwa G, *et al.* Vaccination coverage and timely vaccination with valid doses in Malawi. *Vaccine Reports* 2016;**6**:8–12. doi:https://doi.org/10.1016/j.vacrep.2016.06.001

23 Bar-Zeev N, Swarthout TD, Everett DB, *et al.* Impact and effectiveness of 13-valent pneumococcal conjugate vaccine on population incidence of vaccine and non-vaccine serotype invasive pneumococcal disease in Blantyre, Malawi, 2006–18: prospective observational time-series and case-control stud. *Lancet Glob Heal* 2021;**9**:e989–98. doi:10.1016/S2214-109X(21)00165-0

24 King C, Bar-Zeev N, Phiri T, *et al.* Population impact and effectiveness of sequential 13-valent pneumococcal conjugate and monovalent rotavirus vaccine introduction on infant mortality: prospective birth cohort studies from Malawi. *BMJ Glob Heal* Published Online First: 2020. doi:10.1136/bmjgh-2020-002669

25 Swarthout TD, Fronterre C, Lourenço J, *et al.* High residual carriage of vaccine-serotype Streptococcus pneumoniae after introduction of pneumococcal conjugate vaccine in Malawi. *Nat Commun* 2020;**11**:2222. doi:10.1038/s41467-020-15786-9

26 Lo SW, Gladstone RA, van Tonder AJ, *et al.* Pneumococcal lineages associated with serotype replacement and antibiotic resistance in childhood invasive pneumococcal

For peer review only

For peer review only - http://bmjopen.bmj.com/site/about/guidelines.xhtml

disease in the post-PCV13 era: an international whole-genome sequencing study. *Lancet Infect Dis* 2019;**19**:759–69. doi:https://doi.org/10.1016/S1473-3099(19)30297-X

27 Musicha P, Cornick JE, Bar-Zeev N, *et al.* Trends in antimicrobial resistance in bloodstream infection isolates at a large urban hospital in Malawi (1998–2016): a surveillance study. *Lancet Infect Dis* 2017;**17**:1042–52. doi:https://doi.org/10.1016/S1473-3099(17)30394-8

28 Lourenço J, Obolski U, Swarthout TD, *et al.* Determinants of high residual post-PCV13 pneumococcal vaccine-type carriage in Blantyre, Malawi: a modelling study. *BMC Med* 2019;**17**:219. doi:10.1186/s12916-019-1450-2

29 WHO. Pneumococcal vaccines WHO position paper – 2012 – Recommendations. *Vaccine* 2012;**30**:4717–8. doi:https://doi.org/10.1016/j.vaccine.2012.04.093

30 Swarthout TD, Ibarz-Pavon A, Kawalazira G, *et al.* A pragmatic health centre-based evaluation comparing the effectiveness of a PCV13 schedule change from 3+0 to 2+1 in a high pneumococcal carriage and disease burden setting in Malawi: a study protocol. *BMJ Open* 2021;**11**:e050312. doi:10.1136/bmjopen-2021-050312

31 WHO. WHO recommends groundbreaking malaria vaccine for children at risk. 2021.https://www.who.int/news/item/06-10-2021-who-recommends-groundbreaking-malaria-vaccine-for-children-at-risk (accessed 12 May 2022).

32 Praet N, Asante KP, Bozonnat M-C, *et al.* Assessing the safety, impact and effectiveness of RTS,S/AS01E malaria vaccine following its introduction in three sub-Saharan African countries: methodological approaches and study set-up. *Malar J* 2022;**21**:132. doi:10.1186/s12936-022-04144-3

33 RTS SCTP. Efficacy and safety of RTS,S/AS01 malaria vaccine with or without a booster dose in infants and children in Africa: final results of a phase 3, individually randomised, controlled trial. *Lancet* 2015;**386**:31–45. doi:10.1016/S0140-6736(15)60721-8

34 Guerra Mendoza Y, Garric E, Leach A, *et al.* Safety profile of the RTS,S/AS01 malaria vaccine in infants and children: additional data from a phase III randomized controlled trial in sub-Saharan Africa. *Hum Vaccin Immunother* 2019;**15**:2386–98. doi:10.1080/21645515.2019.1586040

35 National Statistical Office. 2018 Malawi Population and Housing Census.

For peer review only - http://bmjopen.bmj.com/site/about/guidelines.xhtml

2019.https://malawi.unfpa.org/sites/default/files/resource-pdf/2018 Malawi Population and Housing Census Main Report %281%29.pdf (accessed 4 Jul 2022).

36 Mamba KC, Muula AS, Stones W. Facility-imposed barriers to early utilization of focused antenatal care services in Mangochi District, Malawi – a mixed methods assessment. *BMC Pregnancy Childbirth* 2017;**17**:444. doi:10.1186/s12884-017-1631-y

37 Satzke C, Turner P, Virolainen-Julkunen A, *et al.* Standard method for detecting upper respiratory carriage of Streptococcus pneumoniae: Updated recommendations from the World Health Organization Pneumococcal Carriage Working Group. *Vaccine* 2013;**32**:165–79. doi:https://doi.org/10.1016/j.vaccine.2013.08.062

38 United Nations. Nagoya Protocol on Access to Genetic Resources and the Fair and Equitable Sharing of Benefits Arising from their Utilization (ABS) to the Convention on Biological Diversity. 2022.https://www.cbd.int/abs/about/ (accessed 7 Oct 2022).

39 Buckley BS, Henschke N, Bergman H, *et al.* Impact of vaccination on antibiotic usage: a systematic review and meta-analysis. *Clin Microbiol Infect* 2019;**25**:1213–25. doi:10.1016/j.cmi.2019.06.030

40 Jansen KU, Gruber WC, Simon R, *et al.* The impact of human vaccines on bacterial antimicrobial resistance. A review. *Environ Chem Lett* 2021;**19**:4031–62. doi:10.1007/s10311-021-01274-z

41 Lewnard JA, Lo NC, Arinaminpathy N, *et al.* Childhood vaccines and antibiotic use in low- and middle-income countries. *Nature* 2020;**581**:94–9. doi:10.1038/s41586-020-2238-4

42 Davies NG, Flasche S, Jit M, *et al.* Modeling the effect of vaccination on selection for antibiotic resistance in Streptococcus pneumoniae. *Sci Transl Med* Published Online First: 2021. doi:10.1126/scitranslmed.aaz8690

43 Weinberger DM, Malley R, Lipsitch M. Serotype replacement in disease after pneumococcal vaccination. *Lancet* 2011;**378**:1962–73. doi:10.1016/S0140-6736(10)62225-8

44 Torgerson DJ. Contamination in trials: is cluster randomisation the answer? *BMJ* 2001;**322**:355–7. doi:10.1136/bmj.322.7282.355

45 Neville R, Neville J. What can health care professionals in the United Kingdom learn from Malawi? Hum. Resour. Health. 2009;**7**:26. doi:10.1186/1478-4491-7-26

46 Khuluza F, Haefele-Abah C. The availability, prices and affordability of essential

For peer review only

For peer review only - http://bmjopen.bmj.com/site/about/guidelines.xhtml

medicines in Malawi: A cross-sectional study. *PLoS One* 2019;**14**:e0212125. doi:10.1371/journal.pone.0212125

For peer review only

For peer review only - http://bmjopen.bmj.com/site/about/guidelines.xhtml

Figure 1. Malawi split by 28 district and city regions

Red shading indicates the Mangochi (bordering Lake Malawi, shaded in blue) and Blantyre districts (central Southern Malawi) where the IVAR study will be conducted.

Figure 2. Blantyre District with boundaries of 28 health centre catchment areas

Red cross = health centre. Blue and red shading indicate 2+1 and 3+0 PVC13 schedule areas randomly selected for this study, respectively. Green areas are non-inhabited (including mountains, industrial zones, and other regions administratively declared not for habitation). Adapted from Swarthout *et al.* (2021) [30].

Figure 3. Mangochi district with boundaries of 29 health centre catchment areas

Red cross = health centre. Red and yellow shading indicate RTS,S/AS01 malaria vaccine exposed and unexposed areas randomly selected for this study, respectively. Blue shading corresponds to Lake Malawi and Lake Malombe. Green areas are protected zones (e.g., wildlife reserves and national parks etc.).

Figure 4. IVAR study sampling frame

PCV = pneumococcal conjugate vaccine, RTS,S/AS01 = malaria vaccine, m=months, RDT = rapid diagnostic test.

Figure 5. Example of an antibiotic picture (azithromycin) which will be used in the drug bag exercise as part of the community carriage survey

Method adapted from Dixon *et al.* (2019)[16].

Figure 6. Flow diagram of questionnaire workflow of the 'drug bag' component of the community carriage survey

Each orange number corresponds to a round of questioning, starting with all locally identified, available antibiotics being presented to participant's parents/caregivers. The pool of antibiotics available for review is expected to diminish in each successive round, with the final round (round 5) being included as a final check that no child has been given antibiotics within the 14 days prior to sampling, which would render them ineligible for survey participation.

For peer review only

For peer review only - http://bmjopen.bmj.com/site/about/guidelines.xhtml

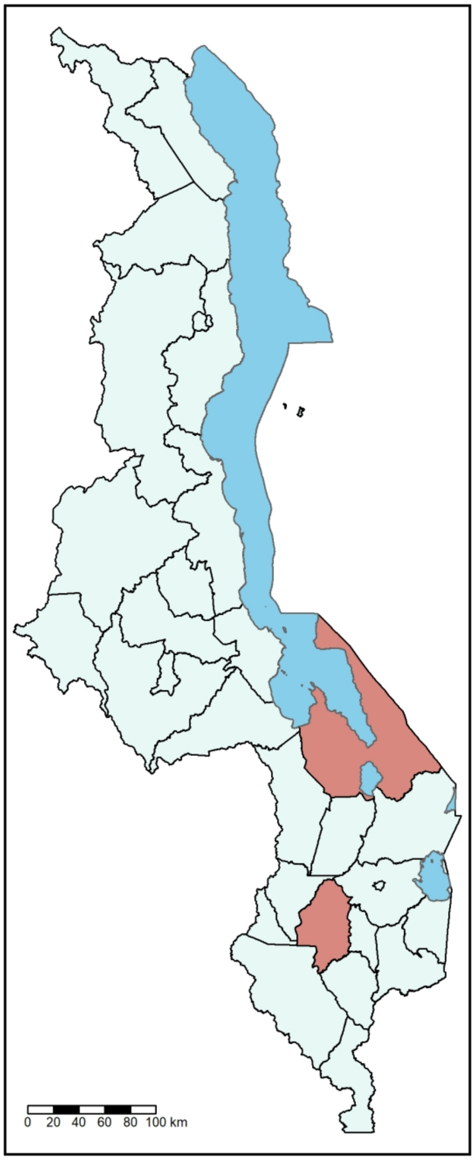

Malawi split by 28 district and city regions. Red shading indicates the Mangochi (bordering Lake Malawi, shaded in blue) and Blantyre districts (central Southern Malawi) where the IVAR study will be conducted.

254x508mm (118 x 118 DPI)

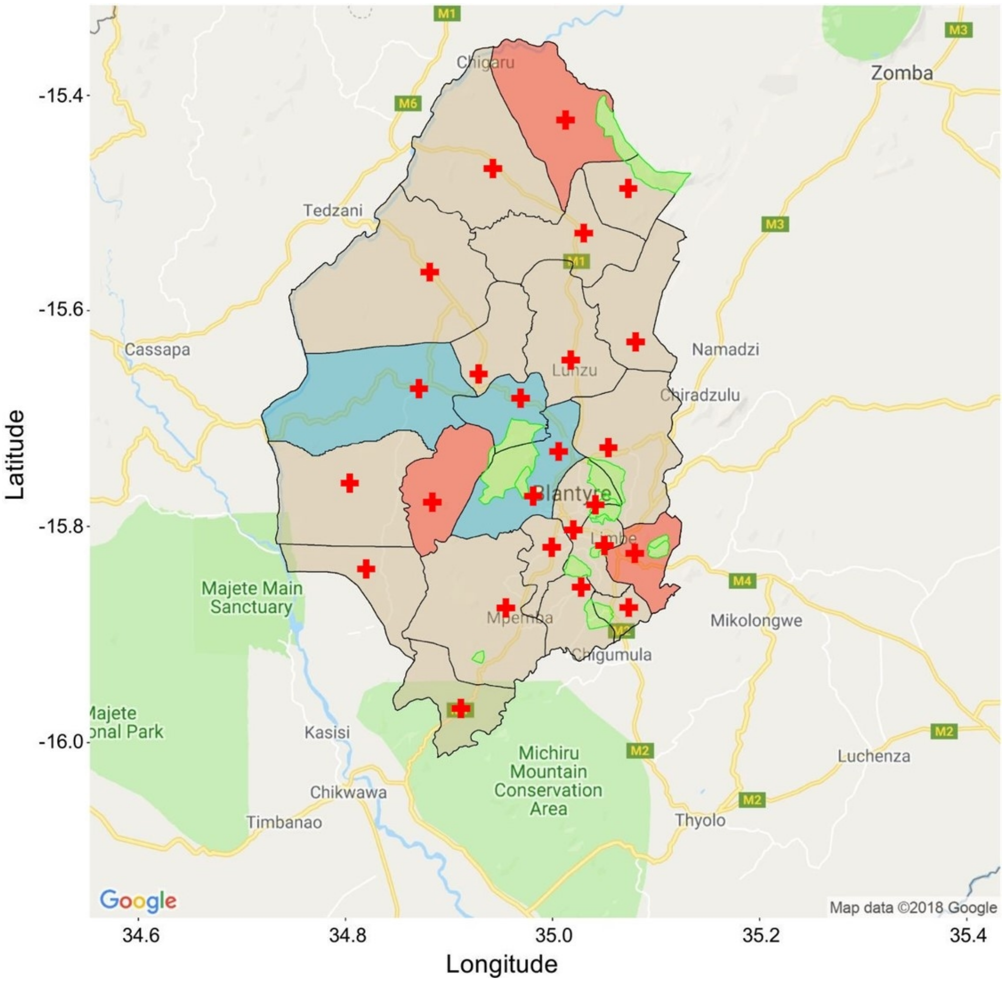

Blantyre District with boundaries of 28 health centre catchment areas. Red cross = health centre. Blue and red shading indicate 2+1 and 3+0 PVC13 schedule areas randomly selected for this study, respectively. Green areas are non-inhabited (including mountains, industrial zones, and other regions administratively declared not for habitation). Adapted from Swarthout et al. (2021) [30].

195x190mm (150 x 150 DPI)

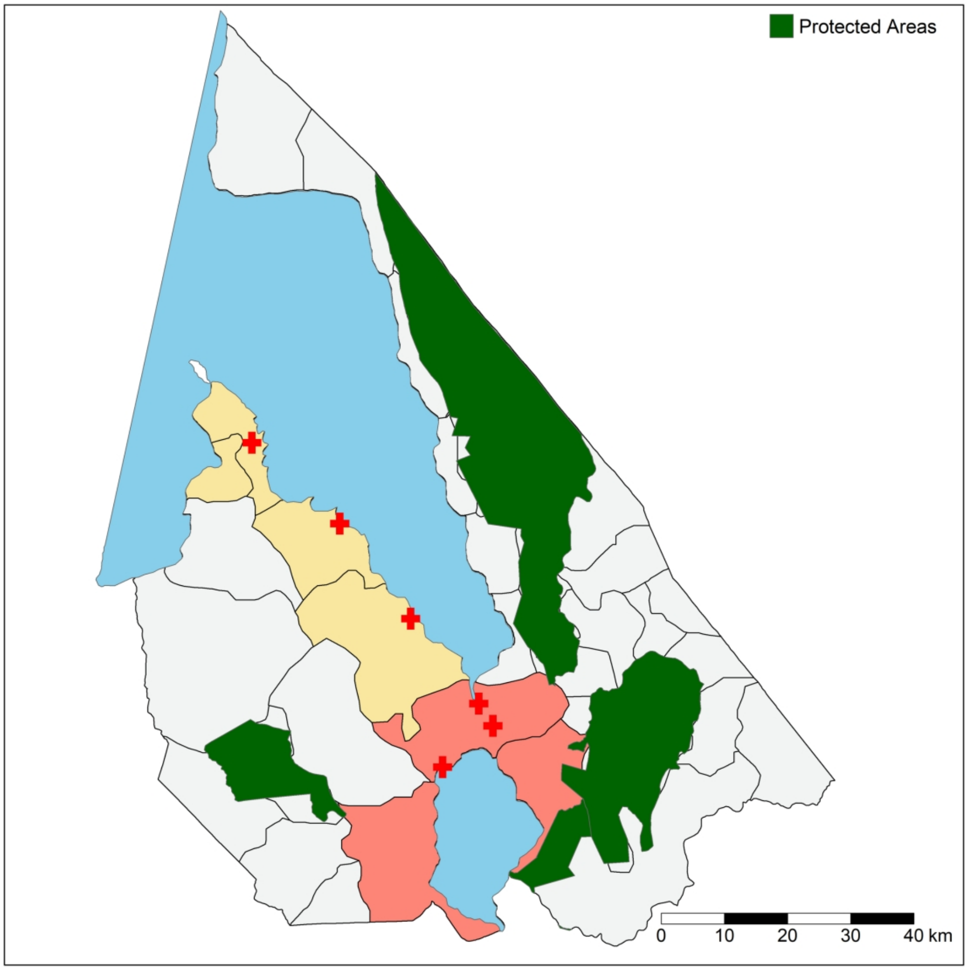

Mangochi district with boundaries of 29 health centre catchment areas. Red cross = health centre. Red and yellow shading indicate RTS,S/AS01 malaria vaccine exposed and unexposed areas randomly selected for this study, respectively. Blue shading corresponds to Lake Malawi and Lake Malombe. Green areas are protected zones (e.g., wildlife reserves and national parks etc.).

483x483mm (130 x 130 DPI)

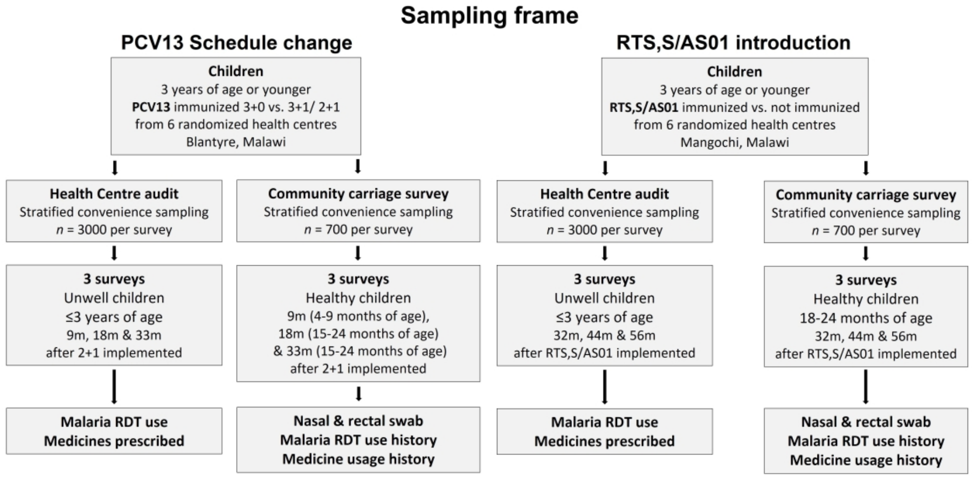

IVAR study sampling frame. PCV = pneumococcal conjugate vaccine, RTS,S/AS01 = malaria vaccine, m=months, RDT = rapid diagnostic test.

355x175mm (330 x 330 DPI)

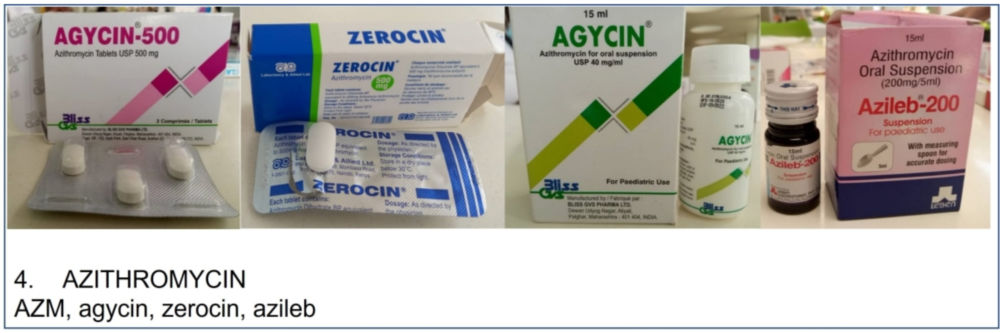

Example of an antibiotic picture (azithromycin) which will be used in the drug bag exercise as part of the community carriage survey. Method adapted from Dixon et al. (2019)[16].

262x87mm (330 x 330 DPI)

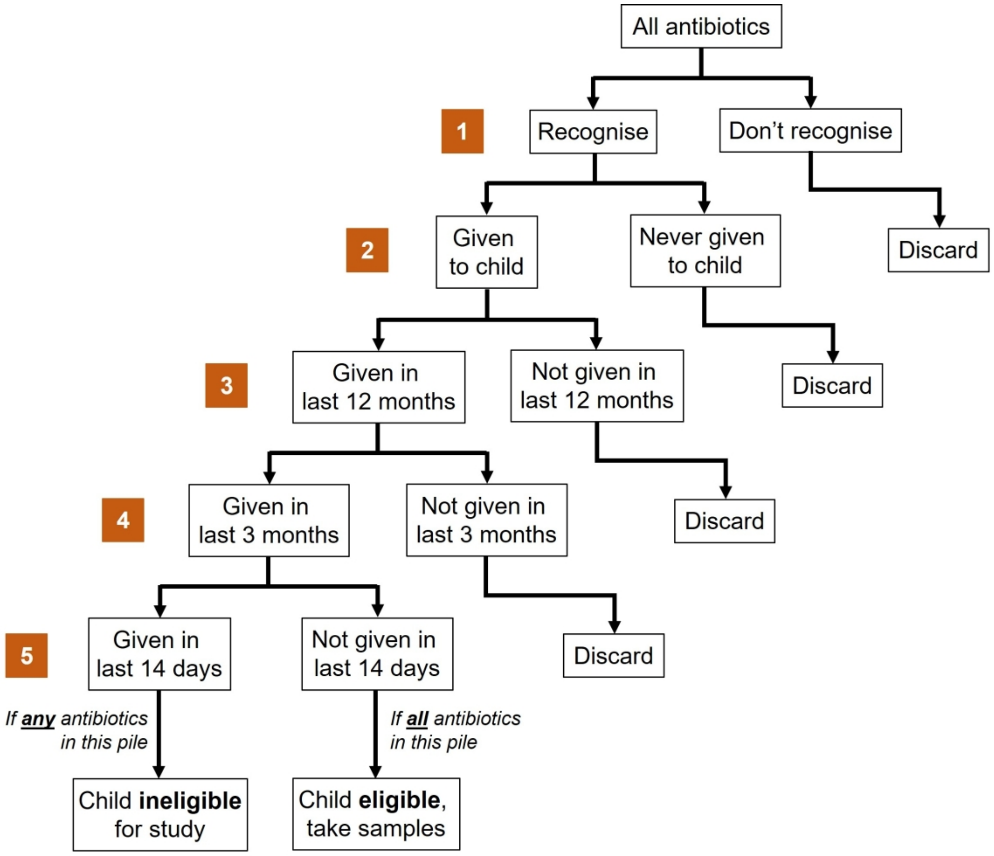

Flow diagram of questionnaire workflow of the 'drug bag' component of the community carriage survey. Each orange number corresponds to a round of questioning, starting with all locally identified, available antibiotics being presented to participant's parents/caregivers. The pool of antibiotics available for review is expected to diminish in each successive round, with the final round (round 5) being included as a final check that no child has been given antibiotics within the 14 days prior to sampling, which would render them ineligible for survey participation.

178x152mm (330 x 330 DPI)

Supplementary file 1:

Case Report Form – Health Center Audit

**Questionnaire ID.**
Label

SCREENING

1	Today's date (dd-mmm-yyyy)	|___|___| - |___|___|___| - | 2 | 0 |___|___|
2	Health Center name	

*Enumerator: The following questions should be answered using the child's Health Passport. If not available, the child is **ineligible** for study participation.*

Inclusion Criteria

3	Is the child's health passport available for review?	**No Yes UNK**
4	Is the child aged 3 years of age or younger?	**No Yes UNK**
5	Has the child presented today for investigation and/or treatment of ill health?	**No Yes UNK**

Exclusion Criteria

6	Has the child presented today for a routine health check?	**No Yes UNK**
7	Has the child presented today for a vaccination?	**No Yes UNK**

Health Passport

8	***Enumerator***<br>*Is the child eligible, including:*<br>• *Health passport **available** for review*<br>• *Aged **3 years of age or under***<br>• *Presenting at the health center for investigation and/or treatment of ill health*<br>***Note:*** *If no, stop review and explain why not eligible.*	**No Yes**

CONSENT

9	*Has the carer had the opportunity to read (or had read to them) the study information sheet?*	**No Yes**
10	*Has the carer had the opportunity to ask questions about this study?*	**No Yes**
11	*Has verbal consent been obtained from carer?*	**No Yes**

HEALTH PASSPORT

Vaccine status

12	Has the child received at least the **initial course (3 doses)** of the **RTS,S/AS01 malaria** vaccination?	**No Yes Not yet eligible UNK**
13	Has the child received the **RTS,S/AS01 malaria booster** vaccination?	**No Yes Not yet eligible UNK**
14	Which **PCV13** vaccination schedule is the child in?	**3+0 2+1 UNK**
15	Has the child received all **PCV13** vaccinations that they are currently eligible for (according to their age)?	**No Yes UNK**

TODAY'S VISIT

The following questions ask about the child's visit to the health center today.

Malaria rapid diagnostic test use:

20	Was a **malaria rapid diagnostic test (RDT)** performed **today**?	**No Yes UNK**

Medicinal prescription:

21	Is there any recorded **medicinal** prescription in **today's** visit?	**No Yes UNK**
	If yes, please record medicine(s) prescribed:	

For peer review only - http://bmjopen.bmj.com/site/about/guidelines.xhtml

Supplementary file 1: **Case Report Form – Health Center Audit**

Medicine prescribed	Route of administration	Course length (days)

22	*Form completed by (Enumerator Code):*	*Code*	/___/___/___/
23	*Form completed by:*	*Signature*	

For peer review only

For peer review only - http://bmjopen.bmj.com/site/about/guidelines.xhtml

Supplementary file 2:

Case Report Form – Blantyre Survey 1

Study ID No. Label	Lab ID No. Label

SCREENING

	Today's date (dd-mmm-yyyy)	|___|___| - |___|___|___| - | 2 | 0 |___|___|
1	What is your child's date of birth? (dd-mmm-yyyy)	|___|___| - |___|___|___| - | 2 | 0 |___|___|
1a	What is your child's age? *(Note to enumerator: If date of birth unknown)*	
2	Enumerator: Which PCV vaccine schedule is offered in this cluster?	**3+0 2+1 UNK**

Inclusion Criteria

3	Has your child received a full initial course of the PCV vaccine?	**No Yes UNK**
4	Is your child healthy?	**No Yes UNK**

Exclusion Criteria

5	Has your child received (any) antibiotics within the previous 14 days?	**No Yes UNK**
6	Is your child currently on TB treatment?	**No Yes UNK**
7	Has your child been hospitalized for pneumonia within the previous 14 days?	**No Yes UNK**
8	Does your child have a (gross) respiratory tract pathology?	**No Yes UNK**
9	Does your child have a terminal illness?	**No Yes UNK**
10	Has your child been previously recruited into this study during this survey?	**No Yes UNK**
	Enumerator: For children in the 2+1 cluster alone:	
11	Has your child been given the booster PCV vaccine?	**No Yes UNK**

Health Passport

12	***Enumerator***<br>*Is the child eligible, including:*<br>• *Aged **4-9 months***<br>• *Permanent resident in Blantyre District*<br>• *Evidence of having received a full initial course (2 or 3 doses) of PCV vaccination*<br>• *No antibiotic use/pneumonia in last 14 days*<br>• *Not currently on TB treatment*<br>• *For children in the 2+1 vaccine cluster, they must **NOT** yet have received the booster vaccination*<br>***Note:** If no, stop interview and explain why not eligible.*	**No Yes**

RECRUITMENT - PRELIMINARY DATA

13	*Was consent obtained from carer?*	**No Yes**
14	*If yes, scan the barcode for Participant ID*	
15	*If scanner not available, write the Participant ID*	**EVAL - 1 - __ __**

For peer review only

For peer review only - http://bmjopen.bmj.com/site/about/guidelines.xhtml

16	Has this child been previously recruited into this study at any time?	No Yes UNK
	(Enumerator: A participant cannot be recruited into study during same survey)	

RECRUITMENT - METADATA

Child Characteristics

17	What is the sex of this child?	Male Female
18	Has the child ever tested positive for HIV?	No Yes UNK

Mother's HIV status (if mother is the carer consenting)

19	Have you ever tested positive for HIV? *Enumerator: Only ask this question if interviewing the mother.*	No Yes UNK N/A
20	Was your HIV-infection confirmed before the recruited child was born? *Enumerator: Only ask this question if date not known.*	No Yes UNK

The following questions are about vaccines your child may have received as part of the routine EPI.

21	Do you have the child's Health Passport with you?	*No Yes*
22	*Enumerator: Are you able to confirm PCV vaccination dates by Health Passport?*	*No Yes*
23	*Enumerator: If yes, take a photo of the vaccination page of Health passport*	*Not Done Done*

Vaccine status

	Vaccine	*Vaccines received (Circle answer)*		*Date of Vaccination (dd-mmm-yyyy)*
	Birth / first contact			
24a	*BCG*	***No Yes UNK***	24b	|__|__| - |__|__|__| - |__|__|__|__|
25a	*OPV0 0*	***No Yes UNK***	25b	|__|__| - |__|__|__| - |__|__|__|__|
	6 weeks of age			
26a	*OPV1*	***No Yes UNK***	26b	|__|__| - |__|__|__| - |__|__|__|__|
27a	*Rota1*	***No Yes UNK***	27b	|__|__| - |__|__|__| - |__|__|__|__|
28a	*DPT-HepB-Hib1*	***No Yes UNK***	28b	|__|__| - |__|__|__| - |__|__|__|__|
29a	*PCV1*	***No Yes UNK***	29b	|__|__| - |__|__|__| - |__|__|__|__|
	10 weeks of age			
30a	*OPV2*	***No Yes UNK***	30b	|__|__| - |__|__|__| - |__|__|__|__|
31a	*Rota2*	***No Yes UNK***	31b	|__|__| - |__|__|__| - |__|__|__|__|
32a	*DPT-HepB-Hib*	***No Yes UNK***	32b	|__|__| - |__|__|__| - |__|__|__|__|
33a	*PCV2 (not in 2+1)*	***No Yes UNK***	33b	|__|__| - |__|__|__| - |__|__|__|__|
	14 weeks of age			
34a	*OPV3*	***No Yes UNK***	34b	|__|__| - |__|__|__| - |__|__|__|__|
35a	*DPT-HepB-Hib 3*	***No Yes UNK***	35b	|__|__| - |__|__|__| - |__|__|__|__|
36a	*PCV 3 (2 in 2+1)*	***No Yes UNK***	36b	|__|__| - |__|__|__| - |__|__|__|__|

For peer review only - http://bmjopen.bmj.com/site/about/guidelines.xhtml

Supplementary file 2: **Case Report Form – Blantyre Survey 1**

37a	*IPV*	***No Yes UNK***	37b	|___|___| - |___|___|___| - |___|___|___|___|
	5-7 months of age			
38a	*Malaria 1*	***No Yes UNK***	38b	|___|___| - |___|___|___| - |___|___|___|___|
39a	*Malaria 2*	***No Yes UNK***	39b	|___|___| - |___|___|___| - |___|___|___|___|
40a	*Malaria 3*	***No Yes UNK***	40b	|___|___| - |___|___|___| - |___|___|___|___|
	9-11 months of age			
41a	*Measle+Rubella 1*	***No Yes UNK***	41b	|___|___| - |___|___|___| - |___|___|___|___|
43a	*PCV 3 (2+1)*	***No Yes UNK***	43b	|___|___| - |___|___|___| - |___|___|___|___|
	15-23 months of age			
44a	*Measles+Rubella 2*	***No Yes UNK***	44b	|___|___| - |___|___|___| - |___|___|___|___|
46a	*Malaria 4*	***No Yes UNK***	46b	|___|___| - |___|___|___| - |___|___|___|___|

Household information

The following questions will be about the house your child lives in, including who lives in the home and its location.

47	*GPS coordinates*	***lat*** |__|__|__|.|__|__|__|__| / ***long*** |__|__|__|.|__|__|__|__|
47a	*Enumerator: If no GPS coordinates available, record why not available.*	

48	How many bedrooms does the child's main house have?	
49	How many adults (16+ years of age) live in the main house?	
50	How many children 5-15 years of age live in the main house, including child recruited today?	
51	How many children 0-4 years of age live in the main house?	

Smoking

52	Does anybody in the child's household smoke tobacco (cigarettes, pipes, or cigars)?	**No Yes**

The following questions ask about the type of house the child lives in.

53 **What type of exterior wall does the house have?**

1 Burnt brick — 4 Plastered thin mud — 7 Iron sheets
2 Unburnt brick — 5 Bamboo — 8 Concrete blocks
3 Pounded thick mud — 6 Grass or no walls — 99 Other, specify: ____________________

54 **What type of roof does the house have?**

1 Grass or leaves — 3 Grass+plastic sheet
2 Grass+Iron sheets — 4 Iron sheets or tiles

55 **What is the condition of the roof?**

1 Good — 2 Poor (leaks water)

56 **What type of floor does the house have inside?**

1 Mud — 3 Tiles
2 Concrete/ cement — 99 other (specify): ____________________

57 **What type of toilet does the house have?**

1 Simple pit latrine — 3 Water toilet

For peer review only - http://bmjopen.bmj.com/site/about/guidelines.xhtml

	2 VIP	3 None (including use the neighbour's toilet)	
58	**What source of electricity does the house have?**		
	1 Escom	3 None	
	2 Solar		
59	**What source of drinking water does the house have?**		
	1 Tap to house	3 Bore hole	5 Open well
	2 Shared communal tap	4 Covered well	6 River
60	**Does the house have glass windows?**		
	0 No	1 Yes	

Possessions

The following questions ask about some possessions you may have. We are not able to give you any of these items, even if you report not having them.

61	Are you comfortable answering questions about items owned by people in your household?	**No Yes**

Does anyone in the household possess any of the following **working** items?

62	Watch or clock	**No Yes**	73	Bed	**No Yes**
63	Radio	**No Yes**	74	Upholstered chair/sofa	**No Yes**
64	Bank account (or bank book)	**No Yes**	75	Table	**No Yes**
65	Charcoal iron	**No Yes**	76	Bicycle	**No Yes**
66	Sewing machine	**No Yes**	77	Motorbike	**No Yes**
67	Mobile phone	**No Yes**	78	Car	**No Yes**
68	Tape/CD player	**No Yes**	79	Television	**No Yes**
69	Fan, electric	**No Yes**	80	Refrigerator	**No Yes**
70	Mosquito net	**No Yes**	81	Other electric items	**No Yes**
71	Number of mosquito nets		82	If other **working** electrical items, specify:	
72	Mattress	**No Yes**			

Education

The following questions ask about the head of your household's education. It maybe you, or it may be someone else

83	Are you comfortable answering questions about the head of your household's education?	**No Yes**

84	**What is the highest educational qualification the household head has acquired?**			
	1 None	3 JCE	5 Non-university diploma	7 Postgraduate degree
	2 PSLCE	4 MSCE	6 University diploma/degree	
85	**Is the household head able to read and write in English?**			
	1 No	2 Yes		

MALARIA, FEBRILE ILLNESS & MEDICINE USE

The following questions ask about your child's history of malaria and/or febrile illness, and their use of medicines.

Body temperature history and malaria rapid diagnostic test use:

86	*Enumerator: If the child's Health Passport is available, are there any occasions where their **body temperature** has been recorded?*	No Yes

For peer review only - http://bmjopen.bmj.com/site/about/guidelines.xhtml

87 *If yes, please record date(s) of recording(s) and temperature:*

Date of recording (dd-mmm-yyyy)	*Temperature Recorded (°C)*
|___|___| - |___|___|___| - |___|___|___|___|	
|___|___| - |___|___|___| - |___|___|___|___|	
|___|___| - |___|___|___| - |___|___|___|___|	
|___|___| - |___|___|___| - |___|___|___|___|	
|___|___| - |___|___|___| - |___|___|___|___|	
|___|___| - |___|___|___| - |___|___|___|___|	
|___|___| - |___|___|___| - |___|___|___|___|	
|___|___| - |___|___|___| - |___|___|___|___|	

88	*Enumerator: If the child's Health Passport is available, are there any recorded usages of **malaria rapid diagnostic tests (RDT)**?*	No Yes

89 *If yes, please record date(s) of malaria rapid diagnostic test(s):*

Date of malaria RDT (dd-mmm-yyyy)	*Result*		
|___|___| - |___|___|___| - |___|___|___|___|	*Negative*	*Positive*	*UNK*
|___|___| - |___|___|___| - |___|___|___|___|	*Negative*	*Positive*	*UNK*
|___|___| - |___|___|___| - |___|___|___|___|	*Negative*	*Positive*	*UNK*
|___|___| - |___|___|___| - |___|___|___|___|	*Negative*	*Positive*	*UNK*
|___|___| - |___|___|___| - |___|___|___|___|	*Negative*	*Positive*	*UNK*
|___|___| - |___|___|___| - |___|___|___|___|	*Negative*	*Positive*	*UNK*
|___|___| - |___|___|___| - |___|___|___|___|	*Negative*	*Positive*	*UNK*
|___|___| - |___|___|___| - |___|___|___|___|	*Negative*	*Positive*	*UNK*

Enumerator: The following questions are to be directly asked to the questionnaire respondent.

90	*When did your child last suffer from a fever?*	
91	*Has your child suffered from fever in the last **14 days?***	**No Yes UNK**
91a	*If yes, how many times?*	
91b	*If yes, how many times did they need to see a doctor for a fever in the last **14 days?***	
91c	*If yes, how many times did they have to stay in hospital for fever in the last **14 days?***	
92	*Has your child suffered from fever in the last **14 days to 3 months?***	**No Yes UNK**
92a	*If yes, how many times?*	
92b	*If yes, how many times did they need to see a doctor for a fever in the last **14 days to 3 months?***	
92c	*If yes, how many times did they have to stay in hospital for fever in the last **14 days to 3 months?***	
93	*Has your child suffered from fever in the last **3 to 12 months?***	**No Yes UNK**

For peer review only - http://bmjopen.bmj.com/site/about/guidelines.xhtml

Supplementary file 2: **Case Report Form – Blantyre Survey 1**

93a	*If yes, how many times?*	
93b	*If yes, how many times did they need to see a doctor for a fever in the last **3 to 12 months?***	
93c	*If yes, how many times did they have to stay in hospital for fever in the last **3 to 12 months?***	

Medicine use:

94	*Enumerator: If the child's Health Passport is available, are there any recorded prescription of **medicines**?*	*No Yes*

95 *If yes, please record date(s) of medicine prescription:*

Date of medicine prescription (dd-mmm-yyyy)	*Medicine prescribed*	*Diagnosis (if stated)*	*Route of administration*	*Course length (days)*
|__|__| - |__|__|__| - |__|__|__|__|				
|__|__| - |__|__|__| - |__|__|__|__|				
|__|__| - |__|__|__| - |__|__|__|__|				
|__|__| - |__|__|__| - |__|__|__|__|				
|__|__| - |__|__|__| - |__|__|__|__|				
|__|__| - |__|__|__| - |__|__|__|__|				
|__|__| - |__|__|__| - |__|__|__|__|				
|__|__| - |__|__|__| - |__|__|__|__|				

Enumerator: The following questions are to be directly asked to the questionnaire respondent

96	*Other than those listed within your child's health passport, have you ever given your child any other **medicines**?*	No Yes UNK
96a	*If yes, what **medicines** have you given?*	
	*Enumerator: If the health passport is **NOT** available, the following questions are to be directly asked to the questionnaire respondent:*	
97	*Has your child been given **antibiotics** in the last **14 days to 3 months?***	***No Yes***
97a	*If yes, what antibiotics (active substance)?*	
97b	*If yes, how many courses (prescriptions) of antibiotics have they received in the last **14 days to 3 months?***	
97c	*Why was your child given antibiotics?*	
98	*Has your child been given **antibiotics** in the last **3 to 12 months?***	***No Yes***
98a	*If yes, what antibiotics (active substance)?*	
98b	*If yes, how many courses (prescriptions) of antibiotics have they received in the last **3 to 12 months?***	
98c	*Why was your child given antibiotics?*	

Antibiotic drug bag capture method:

Enumerator: These questions are to be asked to ALL study participants.

99 *We would now like to ask you further questions about **antibiotics**, and would like to show you some **antibiotics** that we have brought with us (Enumerator: Present antibiotic library to responder). We will*

review only

be asking you to sort these antibiotics into different piles. This is not a test of your knowledge, but to find out whether you recognise these drugs, and whether you have given them to your child. We are carrying out this exercise to help you remember which ones you might have given to your child.

100 *Which of the antibiotics in front of you do you **recognise**? Please pick the ones you **recognise** and put them into one pile.*

Enumerator: The list below is representative of potentially available antibiotics; the actual list will vary according to local availability.

Antibiotic (Formulation)	Recognise		Antibiotic (Formulation)	Recognise	
Amoxicillin (Tablets)	No	Yes	Cloxacillin (Tablets)	No	Yes
Amoxicillin (Suspension)	No	Yes	Cloxacillin (Suspension)	No	Yes
Ampicillin (Tablets)	No	Yes	Cotrimoxazole (Tablets)	No	Yes
Azithromycin (Tablets)	No	Yes	Cotrimoxazole (Suspension)	No	Yes
Benzathene Penicillin (Injectable)	No	Yes	Doxycycline (Tablets)	No	Yes
Benzylpenicillin (Injectable)	No	Yes	Erythromycin (Tablets)	No	Yes
Cefalexin (Tablets)	No	Yes	Erythromycin (Suspension)	No	Yes
Cefixime (Tablets)	No	Yes	Flucloxacillin (Tablets)	No	Yes
Ceftriaxone (Injectable)	No	Yes	Flucloxacillin / amoxicillin (Tablets)	No	Yes
Cefuroxime (Tablets)	No	Yes	Gentamicin (Injectable)	No	Yes
Chloramphenicol (Tablets)	No	Yes	Levofloxacin (Tablets)	No	Yes
Chloramphenicol (Injectable)	No	Yes	Metronidazole (Tablets)	No	Yes
Ciprofloxacin (Tablets)	No	Yes	Metronidazole (Suspension)	No	Yes
Clarithromycin (Tablets)	No	Yes	Norfloxacin / metronidazole (Tablets)	No	Yes
Amoxicillin / clavulanic acid (Tablets)	No	Yes	Ofloxacin / ornidazole (Tablets)	No	Yes
Clindamycin (Tablets)	No	Yes	Phenoxymethylpenicillin (Tablets)	No	Yes
Clindamycin (Injectable)	No	Yes	Tetracycline (Tablets)	No	Yes

Enumerator: Remove the unrecognised pile and put the recognised pile in front of the respondent.

101 *Which of the antibiotics in front of you have you **ever given** to your child? Please pick the ones you have **ever given** to your child and put them into one pile.*

Antibiotic (Formulation)	Recognise		Antibiotic (Formulation)	Recognise	
Amoxicillin (Tablets)	No	Yes	Cloxacillin (Tablets)	No	Yes
Amoxicillin (Suspension)	No	Yes	Cloxacillin (Suspension)	No	Yes
Ampicillin (Tablets)	No	Yes	Cotrimoxazole (Tablets)	No	Yes
Azithromycin (Tablets)	No	Yes	Cotrimoxazole (Suspension)	No	Yes
Benzathene Penicillin (Injectable)	No	Yes	Doxycycline (Tablets)	No	Yes
Benzylpenicillin (Injectable)	No	Yes	Erythromycin (Tablets)	No	Yes
Cefalexin (Tablets)	No	Yes	Erythromycin (Suspension)	No	Yes
Cefixime (Tablets)	No	Yes	Flucloxacillin (Tablets)	No	Yes
Ceftriaxone (Injectable)	No	Yes	Flucloxacillin / amoxicillin (Tablets)	No	Yes
Cefuroxime (Tablets)	No	Yes	Gentamicin (Injectable)	No	Yes
Chloramphenicol (Tablets)	No	Yes	Levofloxacin (Tablets)	No	Yes
Chloramphenicol (Injectable)	No	Yes	Metronidazole (Tablets)	No	Yes
Ciprofloxacin (Tablets)	No	Yes	Metronidazole (Suspension)	No	Yes
Clarithromycin (Tablets)	No	Yes	Norfloxacin / metronidazole (Tablets)	No	Yes
Amoxicillin / clavulanic acid (Tablets)	No	Yes	Ofloxacin / ornidazole (Tablets)	No	Yes
Clindamycin (Tablets)	No	Yes	Phenoxymethylpenicillin (Tablets)	No	Yes

For peer review only - http://bmjopen.bmj.com/site/about/guidelines.xhtml

Clindamycin (Injectable)	*No*	*Yes*	*Tetracycline (Tablets)*	*No*	*Yes*

Enumerator: Remove the unused antibiotics and put the used antibiotics in front of the respondent.

102 *Which of the antibiotics in front of you have you to your child in the **last 12 months**? Please pick the ones you have given to your child in the **last 12 months** and put them into one pile.*

Antibiotic (Formulation)	*Recognise*		*Antibiotic (Formulation)*	*Recognise*	
Amoxicillin (Tablets)	*No*	*Yes*	*Cloxacillin (Tablets)*	*No*	*Yes*
Amoxicillin (Suspension)	*No*	*Yes*	*Cloxacillin (Suspension)*	*No*	*Yes*
Ampicillin (Tablets)	*No*	*Yes*	*Cotrimoxazole (Tablets)*	*No*	*Yes*
Azithromycin (Tablets)	*No*	*Yes*	*Cotrimoxazole (Suspension)*	*No*	*Yes*
Benzathene Penicillin (Injectable)	*No*	*Yes*	*Doxycycline (Tablets)*	*No*	*Yes*
Benzylpenicillin (Injectable)	*No*	*Yes*	*Erythromycin (Tablets)*	*No*	*Yes*
Cefalexin (Tablets)	*No*	*Yes*	*Erythromycin (Suspension)*	*No*	*Yes*
Cefixime (Tablets)	*No*	*Yes*	*Flucloxacillin (Tablets)*	*No*	*Yes*
Ceftriaxone (Injectable)	*No*	*Yes*	*Flucloxacillin / amoxicillin (Tablets)*	*No*	*Yes*
Cefuroxime (Tablets)	*No*	*Yes*	*Gentamicin (Injectable)*	*No*	*Yes*
Chloramphenicol (Tablets)	*No*	*Yes*	*Levofloxacin (Tablets)*	*No*	*Yes*
Chloramphenicol (Injectable)	*No*	*Yes*	*Metronidazole (Tablets)*	*No*	*Yes*
Ciprofloxacin (Tablets)	*No*	*Yes*	*Metronidazole (Suspension)*	*No*	*Yes*
Clarithromycin (Tablets)	*No*	*Yes*	*Norfloxacin / metronidazole (Tablets)*	*No*	*Yes*
Amoxicillin / clavulanic acid (Tablets)	*No*	*Yes*	*Ofloxacin / ornidazole (Tablets)*	*No*	*Yes*
Clindamycin (Tablets)	*No*	*Yes*	*Phenoxymethylpenicillin (Tablets)*	*No*	*Yes*
Clindamycin (Injectable)	*No*	*Yes*	*Tetracycline (Tablets)*	*No*	*Yes*

Enumerator: Remove the unused antibiotics and put the used antibiotics in front of the respondent.

103 *Which of the antibiotics in front of you have you to your child in the **last 3 months**? Please pick the ones you have given to your child in the **last 3 months** and put them into one pile.*

Antibiotic (Formulation)	*Recognise*		*Antibiotic (Formulation)*	*Recognise*	
Amoxicillin (Tablets)	*No*	*Yes*	*Cloxacillin (Tablets)*	*No*	*Yes*
Amoxicillin (Suspension)	*No*	*Yes*	*Cloxacillin (Suspension)*	*No*	*Yes*
Ampicillin (Tablets)	*No*	*Yes*	*Cotrimoxazole (Tablets)*	*No*	*Yes*
Azithromycin (Tablets)	*No*	*Yes*	*Cotrimoxazole (Suspension)*	*No*	*Yes*
Benzathene Penicillin (Injectable)	*No*	*Yes*	*Doxycycline (Tablets)*	*No*	*Yes*
Benzylpenicillin (Injectable)	*No*	*Yes*	*Erythromycin (Tablets)*	*No*	*Yes*
Cefalexin (Tablets)	*No*	*Yes*	*Erythromycin (Suspension)*	*No*	*Yes*
Cefixime (Tablets)	*No*	*Yes*	*Flucloxacillin (Tablets)*	*No*	*Yes*
Ceftriaxone (Injectable)	*No*	*Yes*	*Flucloxacillin / amoxicillin (Tablets)*	*No*	*Yes*
Cefuroxime (Tablets)	*No*	*Yes*	*Gentamicin (Injectable)*	*No*	*Yes*
Chloramphenicol (Tablets)	*No*	*Yes*	*Levofloxacin (Tablets)*	*No*	*Yes*
Chloramphenicol (Injectable)	*No*	*Yes*	*Metronidazole (Tablets)*	*No*	*Yes*
Ciprofloxacin (Tablets)	*No*	*Yes*	*Metronidazole (Suspension)*	*No*	*Yes*
Clarithromycin (Tablets)	*No*	*Yes*	*Norfloxacin / metronidazole (Tablets)*	*No*	*Yes*
Amoxicillin / clavulanic acid (Tablets)	*No*	*Yes*	*Ofloxacin / ornidazole (Tablets)*	*No*	*Yes*
Clindamycin (Tablets)	*No*	*Yes*	*Phenoxymethylpenicillin (Tablets)*	*No*	*Yes*
Clindamycin (Injectable)	*No*	*Yes*	*Tetracycline (Tablets)*	*No*	*Yes*

Enumerator: Remove the unused antibiotics and put the used antibiotics in front of the respondent.

For peer review only - http://bmjopen.bmj.com/site/about/guidelines.xhtml

Supplementary file 2: **Case Report Form – Blantyre Survey 1**

104 *Which of the antibiotics in front of you have you to your child in the **last 14 days**? Please pick the ones you have given to your child in the **last 14 days** and put them into one pile.*

Antibiotic (Formulation)	Recognise	Antibiotic (Formulation)	Recognise
Amoxicillin (Tablets)	No Yes	Cloxacillin (Tablets)	No Yes
Amoxicillin (Suspension)	No Yes	Cloxacillin (Suspension)	No Yes
Ampicillin (Tablets)	No Yes	Cotrimoxazole (Tablets)	No Yes
Azithromycin (Tablets)	No Yes	Cotrimoxazole (Suspension)	No Yes
Benzathene Penicillin (Injectable)	No Yes	Doxycycline (Tablets)	No Yes
Benzylpenicillin (Injectable)	No Yes	Erythromycin (Tablets)	No Yes
Cefalexin (Tablets)	No Yes	Erythromycin (Suspension)	No Yes
Cefixime (Tablets)	No Yes	Flucloxacillin (Tablets)	No Yes
Ceftriaxone (Injectable)	No Yes	Flucloxacillin / amoxicillin (Tablets)	No Yes
Cefuroxime (Tablets)	No Yes	Gentamicin (Injectable)	No Yes
Chloramphenicol (Tablets)	No Yes	Levofloxacin (Tablets)	No Yes
Chloramphenicol (Injectable)	No Yes	Metronidazole (Tablets)	No Yes
Ciprofloxacin (Tablets)	No Yes	Metronidazole (Suspension)	No Yes
Clarithromycin (Tablets)	No Yes	Norfloxacin / metronidazole (Tablets)	No Yes
Amoxicillin / clavulanic acid (Tablets)	No Yes	Ofloxacin / ornidazole (Tablets)	No Yes
Clindamycin (Tablets)	No Yes	Phenoxymethylpenicillin (Tablets)	No Yes
Clindamycin (Injectable)	No Yes	Tetracycline (Tablets)	No Yes

*If any antibiotics are in the given in the last 14 days pile, the child is **ineligible** for study participation.*

Nasal Sample Collection:

105	*NP swab collected?*	***No Yes***
106	*If no swab was collected, specify why not.*	
107	*Was the sample you collected 'adequate'?* *(Adequate: swab passed to the back of nasopharynx for at least 3 seconds and twisted 360°)*	***No Yes***
108	*Is there nasal mucus on swab?*	***No Yes***

109	*Scan/enter the Lab barcode*	

Rectal Sample Collection:

110	*Rectal swab collected?*	***No Yes***
111	*If no swab was collected, specify why not.*	
112	*Was the sample you collected 'adequate'?* *(Adequate: swab passed to the rectum for at least 3 seconds and twisted 360°)*	***No Yes***
113	*Is there faeces on swab?*	***No Yes***

114	*Scan/enter the Lab barcode*	

115	*Form completed by (Enumerator Code):*	*Code*	|___|___|___|
116	*Form completed by:*	*Signature*	

For peer review only - http://bmjopen.bmj.com/site/about/guidelines.xhtml

Supplementary file 3: Case Report Form – Blantyre Surveys 2 & 3

Study ID No.	Lab ID No.
Label	Label

SCREENING

	Today's date (dd-mmm-yyyy)	|___|___| - |___|___|___| - | 2 | 0 |___|___|
1	What is your child's date of birth? (dd-mmm-yyyy)	|___|___| - |___|___|___| - | 2 | 0 |___|___|
1a	What is your child's age?<br>*(Note to enumerator: If date of birth unknown)*	
2	*Enumerator: Which PCV vaccine schedule is offered in this cluster?*	3+0 2+1 UNK

Inclusion Criteria

3	Has your child received a full course of the PCV vaccine?	No Yes UNK
	Enumerator: For children in the 2+1 cluster alone:	
4	Has your child been given the booster PCV vaccine?	No Yes UNK
5	Is your child healthy?	No Yes UNK

Exclusion Criteria

6	Has your child received (any) antibiotics within the previous 14 days?	No Yes UNK
7	Is your child currently on TB treatment?	No Yes UNK
8	Has your child been hospitalized for pneumonia within the previous 14 days?	No Yes UNK
9	Does your child have a (gross) respiratory tract pathology?	No Yes UNK
10	Does your child have a terminal illness?	No Yes UNK
11	Has your child been previously recruited into this study during this survey?	No Yes UNK

Health Passport

12	***Enumerator***<br>*Is the child eligible, including:*<br>• *Aged **15-24 months***<br>• *Permanent resident in Blantyre District*<br>• *Evidence of having received a full schedule of PCV vaccination*<br>• *No antibiotic use/pneumonia in last 14 days*<br>• *Not currently on TB treatment*<br>• *For children in the 2+1 vaccine cluster, they **MUST** have received the booster vaccination*<br>***Note:*** *If no, stop interview and explain why not eligible.*	No Yes

RECRUITMENT - PRELIMINARY DATA

13	*Was consent obtained from carer?*	No Yes
14	*If yes, scan the barcode for Participant ID*	
15	*If scanner not available, write the Participant ID*	EVAL - 1 - __ __
16	Has this child been previously recruited into this study at any time?	No Yes UNK

For peer review only

Supplementary file 3: **Case Report Form – Blantyre Surveys 2 & 3**

	(Enumerator: A participant cannot be recruited into study during same survey)	

RECRUITMENT - METADATA

Child Characteristics

17	What is the sex of this child?	**Male Female**
18	Has the child ever tested positive for HIV?	**No Yes UNK**

Mother's HIV status (if mother is the carer consenting)

19	Have you ever tested positive for HIV?<br>*Enumerator: Only ask this question if interviewing the mother.*	**No Yes UNK N/A**
20	Was your HIV-infection confirmed before the recruited child was born?<br>*Enumerator: Only ask this question if date not known.*	**No Yes UNK**

The following questions are about vaccines your child may have received as part of the routine EPI.

21	Do you have the child's Health Passport with you?	*No Yes*
22	*Enumerator: Are you able to confirm PCV vaccination dates by Health Passport?*	*No Yes*
23	*Enumerator: If yes, take a photo of the vaccination page of Health passport*	*Not Done Done*

Vaccine status

	Vaccine	*Vaccines received (Circle answer)*		*Date of Vaccination (dd-mmm-yyyy)*
	Birth / first contact			
24a	*BCG*	***No Yes UNK***	24b	|___|___| - |___|___|___| - |___|___|___|___|
25a	*OPV0*	***No Yes UNK***	25b	|___|___| - |___|___|___| - |___|___|___|___|
	6 weeks of age			
26a	*OPV1*	***No Yes UNK***	26b	|___|___| - |___|___|___| - |___|___|___|___|
27a	*Rota1*	***No Yes UNK***	27b	|___|___| - |___|___|___| - |___|___|___|___|
28a	*DPT-HepB-Hib 1*	***No Yes UNK***	28b	|___|___| - |___|___|___| - |___|___|___|___|
29a	*PCV1*	***No Yes UNK***	29b	|___|___| - |___|___|___| - |___|___|___|___|
	10 weeks of age			
30a	*OPV2*	***No Yes UNK***	30b	|___|___| - |___|___|___| - |___|___|___|___|
31a	*Rota2*	***No Yes UNK***	31b	|___|___| - |___|___|___| - |___|___|___|___|
32a	*DPT-HepB-Hib 2*	***No Yes UNK***	32b	|___|___| - |___|___|___| - |___|___|___|___|
33a	*PCV2 (Not in2+1)*	***No Yes UNK***	33b	|___|___| - |___|___|___| - |___|___|___|___|
	14 weeks of age			
34a	*OPV3*	***No Yes UNK***	34b	|___|___| - |___|___|___| - |___|___|___|___|
35a	*DPT-HepB-Hib 3*	***No Yes UNK***	35b	|___|___| - |___|___|___| - |___|___|___|___|
36a	*PCV 3 (2 in 2+1)*	***No Yes UNK***	36b	|___|___| - |___|___|___| - |___|___|___|___|
37a	*IPV*	***No Yes UNK***	37b	|___|___| - |___|___|___| - |___|___|___|___|
	5-7 months of age			
38a	*Malaria 1*	***No Yes UNK***	38b	|___|___| - |___|___|___| - |___|___|___|___|

For peer review only - http://bmjopen.bmj.com/site/about/guidelines.xhtml

Supplementary file 3: **Case Report Form – Blantyre Surveys 2 & 3**

39a	*Malaria 2*	*No Yes UNK*	39b	|___|___| - |___|___|___| - |___|___|___|___|
40a	*Malaria 3*	*No Yes UNK*	40b	|___|___| - |___|___|___| - |___|___|___|___|
	9-11 months of age			
41a	*Measles-Rubella1*	*No Yes UNK*	41b	|___|___| - |___|___|___| - |___|___|___|___|
43a	*PCV 3 (2+1)*	*No Yes UNK*	43b	|___|___| - |___|___|___| - |___|___|___|___|
	15-23 months of age			
44a	*Measles-Rubella2*	*No Yes UNK*	44b	|___|___| - |___|___|___| - |___|___|___|___|
46a	*Malaria 4*	*No Yes UNK*	46b	|___|___| - |___|___|___| - |___|___|___|___|

Household information

The following questions will be about the house your child lives in, including who lives in the home and its location.

47	*GPS coordinates*	***lat*** *|__|__|__|.|__|__|__|__|* / ***long*** *|__|__|__|.|__|__|__|__|*
47a	*Enumerator: If no GPS coordinates available, record why not available.*	

48	How many bedrooms does the child's main house have?	
49	How many adults (16+ years of age) live in the main house?	
50	How many children 5-15 years of age live in the main house, including child recruited today?	
51	How many children 0-4 years of age live in the main house?	

Smoking

52	Does anybody in the child's household smoke tobacco (cigarettes, pipes, or cigars)?	**No Yes**

The following questions ask about the type of house the child lives in.

53 **What type of exterior wall does the house have?**

1 Burnt brick — 4 Plastered thin mud — 7 Iron sheets
2 Unburnt brick — 5 Bamboo — 8 Concrete blocks
3 Pounded thick mud — 6 Grass or no walls — 99 Other, specify: ____________________

54 **What type of roof does the house have?**

1 Grass or leaves — 3 Grass+plastic sheet
2 Grass+Iron sheets — 4 Iron sheets or tiles

55 **What is the condition of the roof?**

1 Good — 2 Poor (leaks water)

56 **What type of floor does the house have inside?**

1 Mud — 3 Tiles
2 Concrete/ cement — 99 other (specify): ____________________

57 **What type of toilet does the house have?**

1 Simple pit latrine — 3 Water toilet
2 VIP — 3 None (including use the neighbour's toilet)

58 **What source of electricity does the house have?**

1 Escom — 3 None
2 Solar

59 **What source of drinking water does the house have?**

For peer review only - http://bmjopen.bmj.com/site/about/guidelines.xhtml

	1 Tap to house	3 Bore hole	5 Open well
	2 Shared communal tap	4 Covered well	6 River
60	**Does the house have glass windows?**		
	0 No	1 Yes	

Possessions

The following questions ask about some possessions you may have. We are not able to give you any of these items, even if you report not having them.

61	Are you comfortable answering questions about items owned by people in your household?	**No Yes**

Does anyone in the household possess any of the following **working** items?

62	Watch or clock	**No Yes**	73	Bed	**No Yes**
63	Radio	**No Yes**	74	Upholstered chair/sofa	**No Yes**
64	Bank account (or bank book)	**No Yes**	75	Table	**No Yes**
65	Charcoal iron	**No Yes**	76	Bicycle	**No Yes**
66	Sewing machine	**No Yes**	77	Motorbike	**No Yes**
67	Mobile phone	**No Yes**	78	Car	**No Yes**
68	Tape/CD player	**No Yes**	79	Television	**No Yes**
69	Fan, electric	**No Yes**	80	Refrigerator	**No Yes**
70	Mosquito net	**No Yes**	81	Other electric items	**No Yes**
71	Number of mosquito nets		82	If other **working** electrical items, specify:	
72	Mattress	**No Yes**			

Education

The following questions ask about the head of your household's education. It maybe you, or it may be someone else

83	Are you comfortable answering questions about the head of your household's education?	**No Yes**

84	**What is the highest educational qualification the household head has acquired?**				
	1 None	3 JCE	5 Non-university diploma	7 Postgraduate degree
	2 PSLCE	4 MSCE	6 University diploma/degree	
85	**Is the household head able to read and write in English?**			
	1 No	2 Yes		

MALARIA, FEBRILE ILLNESS & MEDICINE USE

The following questions ask about your child's history of malaria and/or febrile illness, and their use of medicines.

Body temperature history and malaria rapid diagnostic test use:

86	*Enumerator: If the child's Health Passport is available, are there any occasions where their* ***body temperature*** *has been recorded?*	No Yes
87	*If yes, please record date(s) of recording(s) and temperature:*	

Date of recording (dd-mmm-yyyy)	*Temperature Recorded (°C)*
/___/___/ - /___/___/___/ - /___/___/___/___/	
/___/___/ - /___/___/___/ - /___/___/___/___/	

For peer review only - http://bmjopen.bmj.com/site/about/guidelines.xhtml

Supplementary file 3: **Case Report Form – Blantyre Surveys 2 & 3**

/___/___/ - /___/___/___/ - /___/___/___/___/	
/___/___/ - /___/___/___/ - /___/___/___/___/	
/___/___/ - /___/___/___/ - /___/___/___/___/	
/___/___/ - /___/___/___/ - /___/___/___/___/	
/___/___/ - /___/___/___/ - /___/___/___/___/	
/___/___/ - /___/___/___/ - /___/___/___/___/	

88	*Enumerator: If the child's Health Passport is available, are there any recorded usages of **malaria rapid diagnostic tests (RDT)**?*	No Yes
89	*If yes, please record date(s) of malaria rapid diagnostic test(s):*	

Date of malaria RDT (dd-mmm-yyyy)	*Result*		
/___/___/ - /___/___/___/ - /___/___/___/___/	*Negative*	*Positive*	*UNK*
/___/___/ - /___/___/___/ - /___/___/___/___/	*Negative*	*Positive*	*UNK*
/___/___/ - /___/___/___/ - /___/___/___/___/	*Negative*	*Positive*	*UNK*
/___/___/ - /___/___/___/ - /___/___/___/___/	*Negative*	*Positive*	*UNK*
/___/___/ - /___/___/___/ - /___/___/___/___/	*Negative*	*Positive*	*UNK*
/___/___/ - /___/___/___/ - /___/___/___/___/	*Negative*	*Positive*	*UNK*
/___/___/ - /___/___/___/ - /___/___/___/___/	*Negative*	*Positive*	*UNK*
/___/___/ - /___/___/___/ - /___/___/___/___/	*Negative*	*Positive*	*UNK*

Enumerator: The following questions are to be directly asked to the questionnaire respondent.

90	*When did your child last suffer from a fever?*	
91	*Has your child suffered from fever in the last **14 days?***	**No Yes UNK**
91a	*If yes, how many times?*	
91b	*If yes, how many times did they need to see a doctor for a fever in the last **14 days?***	
91c	*If yes, how many times did they have to stay in hospital for fever in the last **14 days?***	
92	*Has your child suffered from fever in the last **14 days to 3 months?***	**No Yes UNK**
92a	*If yes, how many times?*	
92b	*If yes, how many times did they need to see a doctor for a fever in the last **14 days to 3 months?***	
92c	*If yes, how many times did they have to stay in hospital for fever in the last **14 days to 3 months?***	
93	*Has your child suffered from fever in the last **3 to 12 months?***	**No Yes UNK**
93a	*If yes, how many times?*	
93b	*If yes, how many times did they need to see a doctor for a fever in the last **3 to 12 months?***	
93c	*If yes, how many times did they have to stay in hospital for fever in the last **3 to 12 months?***	

Medicine use:

For peer review only - http://bmjopen.bmj.com/site/about/guidelines.xhtml

94	*Enumerator: If the child's Health Passport is available, are there any recorded prescription of **medicines**?*	*No Yes*
95	*If yes, please record date(s) of medicine prescription:*	

Date of medicine prescription (dd-mmm-yyyy)	Medicine prescribed	Diagnosis (if stated)	Route of administration	Course length (days)
|__|__| - |__|__|__| - |__|__|__|__|				
|__|__| - |__|__|__| - |__|__|__|__|				
|__|__| - |__|__|__| - |__|__|__|__|				
|__|__| - |__|__|__| - |__|__|__|__|				
|__|__| - |__|__|__| - |__|__|__|__|				
|__|__| - |__|__|__| - |__|__|__|__|				
|__|__| - |__|__|__| - |__|__|__|__|				
|__|__| - |__|__|__| - |__|__|__|__|				

Enumerator: The following questions are to be directly asked to the questionnaire respondent

96	*Other than those listed within your child's health passport, have you ever given your child any other **medicines**?*	**No Yes UNK**
96a	*If yes, what **medicines** have you given?*	
	*Enumerator: If the health passport is **NOT** available, the following questions are to be directly asked to the questionnaire respondent:*	
97	*Has your child been given **antibiotics** in the last **14 days to 3 months?***	***No Yes***
97a	*If yes, what antibiotics (active substance)?*	
97b	*If yes, how many courses (prescriptions) of antibiotics have they received in the last **14 days to 3 months?***	
97c	*Why was your child given antibiotics?*	
98	*Has your child been given **antibiotics** in the last **3 to 12 months?***	***No Yes***
98a	*If yes, what antibiotics (active substance)?*	
98b	*If yes, how many courses (prescriptions) of antibiotics have they received in the last **3 to 12 months?***	
98c	*Why was your child given antibiotics?*	

Antibiotic drug bag capture method:

Enumerator: These questions are to be asked to ALL study participants.

99 *We would now like to ask you further questions about **antibiotics**, and would like to show you some **antibiotics** that we have brought with us (Enumerator: Present antibiotic library to responder). We will be asking you to sort these antibiotics into different piles. This is not a test of your knowledge, but to find out whether you recognise these drugs, and whether you have given them to your child. We are carrying out this exercise to help you remember which ones you might have given to your child.*

100 *Which of the antibiotics in front of you do you **recognise**? Please pick the ones you **recognise** and put them into one pile.*
Enumerator: The list below is representative of potentially available antibiotics; the actual list will vary according to local availability.

Antibiotic (Formulation)	*Recognise*	*Antibiotic (Formulation)*	*Recognise*

For peer review only - http://bmjopen.bmj.com/site/about/guidelines.xhtml

Amoxicillin (Tablets)	*No*	*Yes*	*Cloxacillin (Tablets)*	*No*	*Yes*
Amoxicillin (Suspension)	*No*	*Yes*	*Cloxacillin (Suspension)*	*No*	*Yes*
Ampicillin (Tablets)	*No*	*Yes*	*Cotrimoxazole (Tablets)*	*No*	*Yes*
Azithromycin (Tablets)	*No*	*Yes*	*Cotrimoxazole (Suspension)*	*No*	*Yes*
Benzathene Penicillin (Injectable)	*No*	*Yes*	*Doxycycline (Tablets)*	*No*	*Yes*
Benzylpenicillin (Injectable)	*No*	*Yes*	*Erythromycin (Tablets)*	*No*	*Yes*
Cefalexin (Tablets)	*No*	*Yes*	*Erythromycin (Suspension)*	*No*	*Yes*
Cefixime (Tablets)	*No*	*Yes*	*Flucloxacillin (Tablets)*	*No*	*Yes*
Ceftriaxone (Injectable)	*No*	*Yes*	*Flucloxacillin / amoxicillin (Tablets)*	*No*	*Yes*
Cefuroxime (Tablets)	*No*	*Yes*	*Gentamicin (Injectable)*	*No*	*Yes*
Chloramphenicol (Tablets)	*No*	*Yes*	*Levofloxacin (Tablets)*	*No*	*Yes*
Chloramphenicol (Injectable)	*No*	*Yes*	*Metronidazole (Tablets)*	*No*	*Yes*
Ciprofloxacin (Tablets)	*No*	*Yes*	*Metronidazole (Suspension)*	*No*	*Yes*
Clarithromycin (Tablets)	*No*	*Yes*	*Norfloxacin / metronidazole (Tablets)*	*No*	*Yes*
Amoxicillin / clavulanic acid (Tablets)	*No*	*Yes*	*Ofloxacin / ornidazole (Tablets)*	*No*	*Yes*
Clindamycin (Tablets)	*No*	*Yes*	*Phenoxymethylpenicillin (Tablets)*	*No*	*Yes*
Clindamycin (Injectable)	*No*	*Yes*	*Tetracycline (Tablets)*	*No*	*Yes*

Enumerator: Remove the unrecognised pile and put the recognised pile in front of the respondent.

101 *Which of the antibiotics in front of you have you **ever given** to your child? Please pick the ones you have **ever given** to your child and put them into one pile.*

Antibiotic (Formulation)	*Recognise*		*Antibiotic (Formulation)*	*Recognise*	
Amoxicillin (Tablets)	*No*	*Yes*	*Cloxacillin (Tablets)*	*No*	*Yes*
Amoxicillin (Suspension)	*No*	*Yes*	*Cloxacillin (Suspension)*	*No*	*Yes*
Ampicillin (Tablets)	*No*	*Yes*	*Cotrimoxazole (Tablets)*	*No*	*Yes*
Azithromycin (Tablets)	*No*	*Yes*	*Cotrimoxazole (Suspension)*	*No*	*Yes*
Benzathene Penicillin (Injectable)	*No*	*Yes*	*Doxycycline (Tablets)*	*No*	*Yes*
Benzylpenicillin (Injectable)	*No*	*Yes*	*Erythromycin (Tablets)*	*No*	*Yes*
Cefalexin (Tablets)	*No*	*Yes*	*Erythromycin (Suspension)*	*No*	*Yes*
Cefixime (Tablets)	*No*	*Yes*	*Flucloxacillin (Tablets)*	*No*	*Yes*
Ceftriaxone (Injectable)	*No*	*Yes*	*Flucloxacillin / amoxicillin (Tablets)*	*No*	*Yes*
Cefuroxime (Tablets)	*No*	*Yes*	*Gentamicin (Injectable)*	*No*	*Yes*
Chloramphenicol (Tablets)	*No*	*Yes*	*Levofloxacin (Tablets)*	*No*	*Yes*
Chloramphenicol (Injectable)	*No*	*Yes*	*Metronidazole (Tablets)*	*No*	*Yes*
Ciprofloxacin (Tablets)	*No*	*Yes*	*Metronidazole (Suspension)*	*No*	*Yes*
Clarithromycin (Tablets)	*No*	*Yes*	*Norfloxacin / metronidazole (Tablets)*	*No*	*Yes*
Amoxicillin / clavulanic acid (Tablets)	*No*	*Yes*	*Ofloxacin / ornidazole (Tablets)*	*No*	*Yes*
Clindamycin (Tablets)	*No*	*Yes*	*Phenoxymethylpenicillin (Tablets)*	*No*	*Yes*
Clindamycin (Injectable)	*No*	*Yes*	*Tetracycline (Tablets)*	*No*	*Yes*

Enumerator: Remove the unused antibiotics and put the used antibiotics in front of the respondent.

102 *Which of the antibiotics in front of you have you to your child in the **last 12 months**? Please pick the ones you have given to your child in the **last 12 months** and put them into one pile.*

Antibiotic (Formulation)	*Recognise*		*Antibiotic (Formulation)*	*Recognise*	
Amoxicillin (Tablets)	*No*	*Yes*	*Cloxacillin (Tablets)*	*No*	*Yes*
Amoxicillin (Suspension)	*No*	*Yes*	*Cloxacillin (Suspension)*	*No*	*Yes*
Ampicillin (Tablets)	*No*	*Yes*	*Cotrimoxazole (Tablets)*	*No*	*Yes*
Azithromycin (Tablets)	*No*	*Yes*	*Cotrimoxazole (Suspension)*	*No*	*Yes*

For peer review only - http://bmjopen.bmj.com/site/about/guidelines.xhtml

Benzathene Penicillin (Injectable)	*No*	*Yes*	*Doxycycline (Tablets)*	*No*	*Yes*
Benzylpenicillin (Injectable)	*No*	*Yes*	*Erythromycin (Tablets)*	*No*	*Yes*
Cefalexin (Tablets)	*No*	*Yes*	*Erythromycin (Suspension)*	*No*	*Yes*
Cefixime (Tablets)	*No*	*Yes*	*Flucloxacillin (Tablets)*	*No*	*Yes*
Ceftriaxone (Injectable)	*No*	*Yes*	*Flucloxacillin / amoxicillin (Tablets)*	*No*	*Yes*
Cefuroxime (Tablets)	*No*	*Yes*	*Gentamicin (Injectable)*	*No*	*Yes*
Chloramphenicol (Tablets)	*No*	*Yes*	*Levofloxacin (Tablets)*	*No*	*Yes*
Chloramphenicol (Injectable)	*No*	*Yes*	*Metronidazole (Tablets)*	*No*	*Yes*
Ciprofloxacin (Tablets)	*No*	*Yes*	*Metronidazole (Suspension)*	*No*	*Yes*
Clarithromycin (Tablets)	*No*	*Yes*	*Norfloxacin / metronidazole (Tablets)*	*No*	*Yes*
Amoxicillin / clavulanic acid (Tablets)	*No*	*Yes*	*Ofloxacin / ornidazole (Tablets)*	*No*	*Yes*
Clindamycin (Tablets)	*No*	*Yes*	*Phenoxymethylpenicillin (Tablets)*	*No*	*Yes*
Clindamycin (Injectable)	*No*	*Yes*	*Tetracycline (Tablets)*	*No*	*Yes*

Enumerator: Remove the unused antibiotics and put the used antibiotics in front of the respondent.

103 *Which of the antibiotics in front of you have you to your child in the **last 3 months**? Please pick the ones you have given to your child in the **last 3 months** and put them into one pile.*

Antibiotic (Formulation)	*Recognise*		*Antibiotic (Formulation)*	*Recognise*	
Amoxicillin (Tablets)	*No*	*Yes*	*Cloxacillin (Tablets)*	*No*	*Yes*
Amoxicillin (Suspension)	*No*	*Yes*	*Cloxacillin (Suspension)*	*No*	*Yes*
Ampicillin (Tablets)	*No*	*Yes*	*Cotrimoxazole (Tablets)*	*No*	*Yes*
Azithromycin (Tablets)	*No*	*Yes*	*Cotrimoxazole (Suspension)*	*No*	*Yes*
Benzathene Penicillin (Injectable)	*No*	*Yes*	*Doxycycline (Tablets)*	*No*	*Yes*
Benzylpenicillin (Injectable)	*No*	*Yes*	*Erythromycin (Tablets)*	*No*	*Yes*
Cefalexin (Tablets)	*No*	*Yes*	*Erythromycin (Suspension)*	*No*	*Yes*
Cefixime (Tablets)	*No*	*Yes*	*Flucloxacillin (Tablets)*	*No*	*Yes*
Ceftriaxone (Injectable)	*No*	*Yes*	*Flucloxacillin / amoxicillin (Tablets)*	*No*	*Yes*
Cefuroxime (Tablets)	*No*	*Yes*	*Gentamicin (Injectable)*	*No*	*Yes*
Chloramphenicol (Tablets)	*No*	*Yes*	*Levofloxacin (Tablets)*	*No*	*Yes*
Chloramphenicol (Injectable)	*No*	*Yes*	*Metronidazole (Tablets)*	*No*	*Yes*
Ciprofloxacin (Tablets)	*No*	*Yes*	*Metronidazole (Suspension)*	*No*	*Yes*
Clarithromycin (Tablets)	*No*	*Yes*	*Norfloxacin / metronidazole (Tablets)*	*No*	*Yes*
Amoxicillin / clavulanic acid (Tablets)	*No*	*Yes*	*Ofloxacin / ornidazole (Tablets)*	*No*	*Yes*
Clindamycin (Tablets)	*No*	*Yes*	*Phenoxymethylpenicillin (Tablets)*	*No*	*Yes*
Clindamycin (Injectable)	*No*	*Yes*	*Tetracycline (Tablets)*	*No*	*Yes*

Enumerator: Remove the unused antibiotics and put the used antibiotics in front of the respondent.

104 *Which of the antibiotics in front of you have you to your child in the **last 14 days**? Please pick the ones you have given to your child in the **last 14 days** and put them into one pile.*

Antibiotic (Formulation)	*Recognise*		*Antibiotic (Formulation)*	*Recognise*	
Amoxicillin (Tablets)	*No*	*Yes*	*Cloxacillin (Tablets)*	*No*	*Yes*
Amoxicillin (Suspension)	*No*	*Yes*	*Cloxacillin (Suspension)*	*No*	*Yes*
Ampicillin (Tablets)	*No*	*Yes*	*Cotrimoxazole (Tablets)*	*No*	*Yes*
Azithromycin (Tablets)	*No*	*Yes*	*Cotrimoxazole (Suspension)*	*No*	*Yes*
Benzathene Penicillin (Injectable)	*No*	*Yes*	*Doxycycline (Tablets)*	*No*	*Yes*
Benzylpenicillin (Injectable)	*No*	*Yes*	*Erythromycin (Tablets)*	*No*	*Yes*
Cefalexin (Tablets)	*No*	*Yes*	*Erythromycin (Suspension)*	*No*	*Yes*
Cefixime (Tablets)	*No*	*Yes*	*Flucloxacillin (Tablets)*	*No*	*Yes*

For peer review only - http://bmjopen.bmj.com/site/about/guidelines.xhtml

Supplementary file 3: Case Report Form – Blantyre Surveys 2 & 3

Ceftriaxone (Injectable)	*No*	*Yes*	*Flucloxacillin / amoxicillin (Tablets)*	*No*	*Yes*
Cefuroxime (Tablets)	*No*	*Yes*	*Gentamicin (Injectable)*	*No*	*Yes*
Chloramphenicol (Tablets)	*No*	*Yes*	*Levofloxacin (Tablets)*	*No*	*Yes*
Chloramphenicol (Injectable)	*No*	*Yes*	*Metronidazole (Tablets)*	*No*	*Yes*
Ciprofloxacin (Tablets)	*No*	*Yes*	*Metronidazole (Suspension)*	*No*	*Yes*
Clarithromycin (Tablets)	*No*	*Yes*	*Norfloxacin / metronidazole (Tablets)*	*No*	*Yes*
Amoxicillin / clavulanic acid (Tablets)	*No*	*Yes*	*Ofloxacin / ornidazole (Tablets)*	*No*	*Yes*
Clindamycin (Tablets)	*No*	*Yes*	*Phenoxymethylpenicillin (Tablets)*	*No*	*Yes*
Clindamycin (Injectable)	*No*	*Yes*	*Tetracycline (Tablets)*	*No*	*Yes*
*If any antibiotics are in the given in the last 14 days pile, the child is **ineligible** for study participation.*					

Nasal Sample Collection:

105	*NP swab collected?*	***No Yes***
106	*If no swab was collected, specify why not.*	
107	*Was the sample you collected 'adequate'?*<br>*(Adequate: swab passed to the back of nasopharynx for at least 3 seconds and twisted 360°)*	***No Yes***
108	*Is there nasal mucus on swab?*	***No Yes***

109	*Scan/enter the Lab barcode*	

Rectal Sample Collection:

110	*Rectal swab collected?*	***No Yes***
111	*If no swab was collected, specify why not.*	
112	*Was the sample you collected 'adequate'?*<br>*(Adequate: swab passed to the rectum for at least 3 seconds and twisted 360°)*	***No Yes***
113	*Is there faeces on swab?*	***No Yes***

114	*Scan/enter the Lab barcode*	

115	*Form completed by (Enumerator Code):*	*Code*	|___|___|___|
116	*Form completed by:*	*Signature*	

For peer review only

Supplementary file 4: **Case Report Form – Mangochi Survey**

Study ID No. Label	Lab ID No. Label

SCREENING

	Today's date (dd-mmm-yyyy)	|___|___| - |___|___|___| - | 2 | 0 |___|___|
1	What is your child's date of birth? (dd-mmm-yyyy)	|___|___| - |___|___|___| - | 2 | 0 |___|___|
1a	What is your child's age?<br>*(Note to enumerator: If date of birth unknown)*	
2	*Enumerator: Is this a cluster where the RST,S/AS01 vaccine is implemented?*	**No Yes UNK**

Inclusion Criteria

3	Has your child received a full initial course of the RST,S/AS01 vaccine?	**No Yes UNK**
4	Is your child healthy?	**No Yes UNK**

Exclusion Criteria

5	Has your child received (any) antibiotics within the previous 14 days?	**No Yes UNK**
6	Is your child currently on TB treatment?	**No Yes UNK**
7	Has your child been hospitalized for pneumonia within the previous 14 days?	**No Yes UNK**
8	Does your child have a (gross) respiratory tract pathology?	**No Yes UNK**
9	Does your child have a terminal illness?	**No Yes UNK**
10	Has your child been previously recruited into this study during this survey?	**No Yes UNK**

Health Passport

11	***Enumerator***<br>*Is the child eligible, including:*<br>• *Aged **18-36 months***<br>• *Permanent resident in Mangochi District*<br>• *No antibiotic use/pneumonia in last 14 days*<br>• *Not currently on TB treatment*<br>• *For children in the RTS,S/AS01 vaccine cluster, they **MUST** have received the full initial vaccination course, but are **NOT** required to have received the booster vaccination*<br>***Note:** If no, stop interview and explain why not eligible.*	**No Yes**

RECRUITMENT - PRELIMINARY DATA

12	*Was consent obtained from carer?*	**No Yes**
13	*If yes, scan the barcode for Participant ID*	
14	*If scanner not available, write the Participant ID*	**EVAL - 1 - __ __**
15	Has this child been recruited during a previous survey?<br>*(Enumerator: A participant cannot be recruited into study during same survey)*	**No Yes UNK**

For peer review only

For peer review only - http://bmjopen.bmj.com/site/about/guidelines.xhtml

Supplementary file 4: **Case Report Form – Mangochi Survey**

RECRUITMENT - METADATA

Child Characteristics

16	What is the sex of this child?	**Male Female**
17	Has the child ever tested positive for HIV?	**No Yes UNK**

Mother's HIV status (if mother is the carer consenting)

18	Have you ever tested positive for HIV?<br>*Enumerator: Only ask this question if interviewing the mother.*	**No Yes UNK N/A**
19	Was your HIV-infection confirmed before the recruited child was born?<br>*Enumerator: Only ask this question if date not known.*	**No Yes UNK**

The following questions are about vaccines your child may have received as part of the routine EPI.

20	Do you have the child's Health Passport with you?	**No Yes**
21	*Enumerator: Are you able to confirm RTS,S/AS01 vaccination dates by Health Passport?*	*No Yes*
22	*Enumerator: If yes, take a photo of the vaccination page of Health passport*	*Not Done Done*

Vaccine status

	Vaccine	*Vaccines received* (*Circle answer*)		*Date of Vaccination* (*dd-mmm-yyyy*)
	Birth / first contact			
23a	*BCG*	***No Yes UNK***	23b	|___|___| - |___|___|___| - |___|___|___|___|
24a	*OPV 0*	***No Yes UNK***	24b	|___|___| - |___|___|___| - |___|___|___|___|
	6 weeks of age			
25a	*OPV 1*	***No Yes UNK***	25b	|___|___| - |___|___|___| - |___|___|___|___|
26a	*Rota1*	***No Yes UNK***	26b	|___|___| - |___|___|___| - |___|___|___|___|
27a	*DPT-HepB-Hib1*	***No Yes UNK***	27b	|___|___| - |___|___|___| - |___|___|___|___|
28a	*PCV1*	***No Yes UNK***	28b	|___|___| - |___|___|___| - |___|___|___|___|
	10 weeks of age			
29a	*OPV22*	***No Yes UNK***	29b	|___|___| - |___|___|___| - |___|___|___|___|
30a	*Rota2*	***No Yes UNK***	30b	|___|___| - |___|___|___| - |___|___|___|___|
31a	*DPT-HepB-Hib2*	***No Yes UNK***	31b	|___|___| - |___|___|___| - |___|___|___|___|
32a	*PCV2*	***No Yes UNK***	32b	|___|___| - |___|___|___| - |___|___|___|___|
	14 weeks of age			
33a	*OPV33*	***No Yes UNK***	33b	|___|___| - |___|___|___| - |___|___|___|___|
34a	*DPT-HepB-Hib3*	***No Yes UNK***	34b	|___|___| - |___|___|___| - |___|___|___|___|
35a	*PCV3*	***No Yes UNK***	35b	|___|___| - |___|___|___| - |___|___|___|___|
36a	*IPV*	***No Yes UNK***	36b	|___|___| - |___|___|___| - |___|___|___|___|
	5-7 months of age			

For peer review only - http://bmjopen.bmj.com/site/about/guidelines.xhtml

Supplementary file 4: **Case Report Form – Mangochi Survey**

37a	*Malaria RTS,S/AS01 1*	***No Yes UNK***	37b	/___/___/ - /___/___/___/ - /___/___/___/___/
38a	*Malaria RTS,S/AS01 2*	***No Yes UNK***	38b	/___/___/ - /___/___/___/ - /___/___/___/___/
39a	*Malaria RTS,S/AS01 3*	***No Yes UNK***	39b	/___/___/ - /___/___/___/ - /___/___/___/___/
	9-11 months of age			
40a	*Measles-Rubella1*	***No Yes UNK***	40b	/___/___/ - /___/___/___/ - /___/___/___/___/
	15-23 months of age			
42a	*Measles-Rubella2*	***No Yes UNK***	42b	/___/___/ - /___/___/___/ - /___/___/___/___/
44a	*Malaria RTS,S/AS01 4*	***No Yes UNK***	44b	/___/___/ - /___/___/___/ - /___/___/___/___/

Household information

The following questions will be about the house the child lives in, including who lives in the home and its location

45	*GPS coordinates*	***lat*** */__/__/__/./__/__/__/__/ / **long** /__/__/__/./__/__/__/__/*
45a	*Enumerator: If no GPS coordinates available, record why not available.*	

46	How many bedrooms does the child's main house have?	
47	How many adults (16+ years of age) live in the main house?	
48	How many children 5-15 years of age live in the main house, including child recruited today?	
49	How many children 0-4 years of age live in the main house?	

Smoking

50	Does anybody in the child's household smoke tobacco (cigarettes, pipes, or cigars)?	**No Yes**

The following questions ask about the type of house the child lives in.

51 **What type of exterior wall does the house have?**

1 Burnt brick; 4 Plastered thin mud; 7 Iron sheets
2 Unburnt brick; 5 Bamboo; 8 Concrete blocks
3 Pounded thick mud; 6 Grass or no walls; 99 Other, specify: ______________________

52 **What type of roof does the house have?**

1 Grass or leaves; 3 Grass+plastic sheet
2 Grass+Iron sheets; 4 Iron sheets or tiles

53 **What is the condition of the roof?**

1 Good; 2 Poor (leaks water)

54 **What type of floor does the house have inside?**

1 Mud; 3 Tiles
2 Concrete/ cement; 99 other (specify): ______________________

55 **What type of toilet does the house have?**

1 Simple pit latrine; 3 Water toilet
2 VIP; 3 None (including use the neighbour's toilet)

56 **What source of electricity does the house have?**

1 Escom; 3 None
2 Solar

57 **What source of drinking water does the house have?**

For peer review only - http://bmjopen.bmj.com/site/about/guidelines.xhtml

Supplementary file 4: Case Report Form – Mangochi Survey

	1 Tap to house	3 Bore hole	5 Open well
	2 Shared communal tap	4 Covered well	6 River
58	**Does the house have glass windows?**		
	0 No	1 Yes	

Possessions

The following questions ask about some possessions you may have. We are not able to give you any of these items, even if you report not having them.

59	Are you comfortable answering questions about items owned by people in your household?	**No Yes**

Does anyone in the household possess any of the following **working** items?

60	Watch or clock	**No**	**Yes**	71	Bed	**No**	**Yes**
61	Radio	**No**	**Yes**	72	Upholstered chair/sofa	**No**	**Yes**
62	Bank account (or bank book)	**No**	**Yes**	73	Table	**No**	**Yes**
63	Charcoal iron	**No**	**Yes**	74	Bicycle	**No**	**Yes**
64	Sewing machine	**No**	**Yes**	75	Motorbike	**No**	**Yes**
65	Mobile phone	**No**	**Yes**	76	Car	**No**	**Yes**
66	Tape/CD player	**No**	**Yes**	77	Television	**No**	**Yes**
67	Fan, electric	**No**	**Yes**	78	Refrigerator	**No**	**Yes**
68	Mosquito net	**No**	**Yes**	79	Other electric items	**No**	**Yes**
69	Number of mosquito nets			80	If other **working** electrical items, specify:		
70	Mattress	**No**	**Yes**				

Education

The following questions ask about the head of your household's education. It maybe you, or it may be someone else

81	Are you comfortable answering questions about the head of your household's education?	**No Yes**

82	**What is the highest educational qualification the household head has acquired?**			
	1 None	3 JCE	5 Non-university diploma	7 Postgraduate degree
	2 PSLCE	4 MSCE	6 University diploma/degree	
83	**Is the household head able to read and write in English?**			
	1 No	2 Yes		

MALARIA, FEBRILE ILLNESS & MEDICINE USE

The following questions ask about your child's history of malaria and/or febrile illness, and their use of medicines.

Body temperature history and malaria rapid diagnostic test use:

84	*Enumerator: If the child's Health Passport is available, are there any occasions where their **body temperature** has been recorded?*	No Yes
85	*If yes, please record date(s) of recording(s) and temperature:*	

Date of recording (dd-mmm-yyyy)	*Temperature Recorded (°C)*
/___/___/ - /___/___/___/ - /___/___/___/___/	
/___/___/ - /___/___/___/ - /___/___/___/___/	

For peer review only - http://bmjopen.bmj.com/site/about/guidelines.xhtml

Supplementary file 4: **Case Report Form – Mangochi Survey**

/___/___/ - /___/___/___/ - /___/___/___/___/	
/___/___/ - /___/___/___/ - /___/___/___/___/	
/___/___/ - /___/___/___/ - /___/___/___/___/	
/___/___/ - /___/___/___/ - /___/___/___/___/	
/___/___/ - /___/___/___/ - /___/___/___/___/	
/___/___/ - /___/___/___/ - /___/___/___/___/	

86	*Enumerator: If the child's Health Passport is available, are there any recorded usages of **malaria rapid diagnostic tests (RDT)**?*	No Yes
87	*If yes, please record date(s) of malaria rapid diagnostic test(s):*	

Date of malaria RDT (dd-mmm-yyyy)	*Result*		
/___/___/ - /___/___/___/ - /___/___/___/___/	*Negative*	*Positive*	*UNK*
/___/___/ - /___/___/___/ - /___/___/___/___/	*Negative*	*Positive*	*UNK*
/___/___/ - /___/___/___/ - /___/___/___/___/	*Negative*	*Positive*	*UNK*
/___/___/ - /___/___/___/ - /___/___/___/___/	*Negative*	*Positive*	*UNK*
/___/___/ - /___/___/___/ - /___/___/___/___/	*Negative*	*Positive*	*UNK*
/___/___/ - /___/___/___/ - /___/___/___/___/	*Negative*	*Positive*	*UNK*
/___/___/ - /___/___/___/ - /___/___/___/___/	*Negative*	*Positive*	*UNK*
/___/___/ - /___/___/___/ - /___/___/___/___/	*Negative*	*Positive*	*UNK*

Enumerator: The following questions are to be directly asked to the questionnaire respondent.

88	*When did your child last suffer from a fever?*	
89	*Has your child suffered from fever in the last **14 days?***	**No Yes UNK**
89a	*If yes, how many times?*	
89b	*If yes, how many times did they need to see a doctor for a fever in the last **14 days?***	
89c	*If yes, how many times did they have to stay in hospital for fever in the last **14 days?***	
90	*Has your child suffered from fever in the last **14 days to 3 months?***	**No Yes UNK**
90a	*If yes, how many times?*	
90b	*If yes, how many times did they need to see a doctor for a fever in the last **14 days to 3 months?***	
90c	*If yes, how many times did they have to stay in hospital for fever in the last **14 days to 3 months?***	
91	*Has your child suffered from fever in the last **3 to 12 months?***	**No Yes UNK**
91a	*If yes, how many times?*	
91b	*If yes, how many times did they need to see a doctor for a fever in the last **3 to 12 months?***	
91c	*If yes, how many times did they have to stay in hospital for fever in the last **3 to 12 months?***	

Medicine use:

For peer review only - http://bmjopen.bmj.com/site/about/guidelines.xhtml

Supplementary file 4: **Case Report Form – Mangochi Survey**

92	*Enumerator: If the child's Health Passport is available, are there any recorded prescription of **medicines**?*	*No Yes*
93	*If yes, please record date(s) of medicine prescription:*	

Date of medicine prescription (dd-mmm-yyyy)	*Medicine prescribed*	*Diagnosis (if stated)*	*Route of administration*	*Course length (days)*
|__|__| - |__|__|__| - |__|__|__|__|				
|__|__| - |__|__|__| - |__|__|__|__|				
|__|__| - |__|__|__| - |__|__|__|__|				
|__|__| - |__|__|__| - |__|__|__|__|				
|__|__| - |__|__|__| - |__|__|__|__|				
|__|__| - |__|__|__| - |__|__|__|__|				
|__|__| - |__|__|__| - |__|__|__|__|				
|__|__| - |__|__|__| - |__|__|__|__|				

94	*Other than those listed within your child's health passport, have you ever given your child any other **medicines**?*	**No Yes UNK**
94a	*If yes, what **medicines** have you given?*	
	*Enumerator: If the health passport is **NOT** available, the following questions are to be directly asked to the questionnaire respondent:*	
95	*Has your child been given **antibiotics** in the last **14 days to 3 months?***	***No Yes***
95a	*If yes, what antibiotics (active substance)?*	
95b	*If yes, how many courses (prescriptions) of antibiotics have they received in the last **14 days to 3 months?***	
95c	*Why was your child given antibiotics?*	
96	*Has your child been given **antibiotics** in the last **3 to 12 months?***	***No Yes***
96a	*If yes, what antibiotics (active substance)?*	
96b	*If yes, how many courses (prescriptions) of antibiotics have they received in the last **3 to 12 months?***	
96c	*Why was your child given antibiotics?*	

Antibiotic drug bag capture method:

Enumerator: These questions are to be asked to ALL study participants.

97 *We would now like to ask you further questions about **antibiotics**, and would like to show you some **antibiotics** that we have brought with us (Enumerator: Present antibiotic library to responder). We will be asking you to sort these antibiotics into different piles. This is not a test of your knowledge, but to find out whether you recognise these drugs, and whether you have given them to your child. We are carrying out this exercise to help you remember which ones you might have given to your child.*

98 *Which of the antibiotics in front of you do you **recognise**? Please pick the ones you **recognise** and put them into one pile.*
Enumerator: The list below is representative of potentially available antibiotics; the actual list will vary according to local availability.

Antibiotic (Formulation)	*Recognise*	*Antibiotic (Formulation)*	*Recognise*
Amoxicillin (Tablets)	*No Yes*	*Cloxacillin (Tablets)*	*No Yes*

For peer review only - http://bmjopen.bmj.com/site/about/guidelines.xhtml

Amoxicillin (Suspension)	*No*	*Yes*	*Cloxacillin (Suspension)*	*No*	*Yes*
Ampicillin (Tablets)	*No*	*Yes*	*Cotrimoxazole (Tablets)*	*No*	*Yes*
Azithromycin (Tablets)	*No*	*Yes*	*Cotrimoxazole (Suspension)*	*No*	*Yes*
Benzathene Penicillin (Injectable)	*No*	*Yes*	*Doxycycline (Tablets)*	*No*	*Yes*
Benzylpenicillin (Injectable)	*No*	*Yes*	*Erythromycin (Tablets)*	*No*	*Yes*
Cefalexin (Tablets)	*No*	*Yes*	*Erythromycin (Suspension)*	*No*	*Yes*
Cefixime (Tablets)	*No*	*Yes*	*Flucloxacillin (Tablets)*	*No*	*Yes*
Ceftriaxone (Injectable)	*No*	*Yes*	*Flucloxacillin / amoxicillin (Tablets)*	*No*	*Yes*
Cefuroxime (Tablets)	*No*	*Yes*	*Gentamicin (Injectable)*	*No*	*Yes*
Chloramphenicol (Tablets)	*No*	*Yes*	*Levofloxacin (Tablets)*	*No*	*Yes*
Chloramphenicol (Injectable)	*No*	*Yes*	*Metronidazole (Tablets)*	*No*	*Yes*
Ciprofloxacin (Tablets)	*No*	*Yes*	*Metronidazole (Suspension)*	*No*	*Yes*
Clarithromycin (Tablets)	*No*	*Yes*	*Norfloxacin / metronidazole (Tablets)*	*No*	*Yes*
Amoxicillin / clavulanic acid (Tablets)	*No*	*Yes*	*Ofloxacin / ornidazole (Tablets)*	*No*	*Yes*
Clindamycin (Tablets)	*No*	*Yes*	*Phenoxymethylpenicillin (Tablets)*	*No*	*Yes*
Clindamycin (Injectable)	*No*	*Yes*	*Tetracycline (Tablets)*	*No*	*Yes*

Enumerator: Remove the unrecognised pile and put the recognised pile in front of the respondent.

99 *Which of the antibiotics in front of you have you **ever given** to your child? Please pick the ones you have **ever given** to your child and put them into one pile.*

Antibiotic (Formulation)	*Recognise*		*Antibiotic (Formulation)*	*Recognise*	
Amoxicillin (Tablets)	*No*	*Yes*	*Cloxacillin (Tablets)*	*No*	*Yes*
Amoxicillin (Suspension)	*No*	*Yes*	*Cloxacillin (Suspension)*	*No*	*Yes*
Ampicillin (Tablets)	*No*	*Yes*	*Cotrimoxazole (Tablets)*	*No*	*Yes*
Azithromycin (Tablets)	*No*	*Yes*	*Cotrimoxazole (Suspension)*	*No*	*Yes*
Benzathene Penicillin (Injectable)	*No*	*Yes*	*Doxycycline (Tablets)*	*No*	*Yes*
Benzylpenicillin (Injectable)	*No*	*Yes*	*Erythromycin (Tablets)*	*No*	*Yes*
Cefalexin (Tablets)	*No*	*Yes*	*Erythromycin (Suspension)*	*No*	*Yes*
Cefixime (Tablets)	*No*	*Yes*	*Flucloxacillin (Tablets)*	*No*	*Yes*
Ceftriaxone (Injectable)	*No*	*Yes*	*Flucloxacillin / amoxicillin (Tablets)*	*No*	*Yes*
Cefuroxime (Tablets)	*No*	*Yes*	*Gentamicin (Injectable)*	*No*	*Yes*
Chloramphenicol (Tablets)	*No*	*Yes*	*Levofloxacin (Tablets)*	*No*	*Yes*
Chloramphenicol (Injectable)	*No*	*Yes*	*Metronidazole (Tablets)*	*No*	*Yes*
Ciprofloxacin (Tablets)	*No*	*Yes*	*Metronidazole (Suspension)*	*No*	*Yes*
Clarithromycin (Tablets)	*No*	*Yes*	*Norfloxacin / metronidazole (Tablets)*	*No*	*Yes*
Amoxicillin / clavulanic acid (Tablets)	*No*	*Yes*	*Ofloxacin / ornidazole (Tablets)*	*No*	*Yes*
Clindamycin (Tablets)	*No*	*Yes*	*Phenoxymethylpenicillin (Tablets)*	*No*	*Yes*
Clindamycin (Injectable)	*No*	*Yes*	*Tetracycline (Tablets)*	*No*	*Yes*

Enumerator: Remove the unused antibiotics and put the used antibiotics in front of the respondent.

100 *Which of the antibiotics in front of you have you to your child in the **last 12 months**? Please pick the ones you have given to your child in the **last 12 months** and put them into one pile.*

Antibiotic (Formulation)	*Recognise*		*Antibiotic (Formulation)*	*Recognise*	
Amoxicillin (Tablets)	*No*	*Yes*	*Cloxacillin (Tablets)*	*No*	*Yes*
Amoxicillin (Suspension)	*No*	*Yes*	*Cloxacillin (Suspension)*	*No*	*Yes*
Ampicillin (Tablets)	*No*	*Yes*	*Cotrimoxazole (Tablets)*	*No*	*Yes*
Azithromycin (Tablets)	*No*	*Yes*	*Cotrimoxazole (Suspension)*	*No*	*Yes*
Benzathene Penicillin (Injectable)	*No*	*Yes*	*Doxycycline (Tablets)*	*No*	*Yes*

For peer review only - http://bmjopen.bmj.com/site/about/guidelines.xhtml

Supplementary file 4: Case Report Form – Mangochi Survey

Benzylpenicillin (Injectable)	*No*	*Yes*	*Erythromycin (Tablets)*	*No*	*Yes*
Cefalexin (Tablets)	*No*	*Yes*	*Erythromycin (Suspension)*	*No*	*Yes*
Cefixime (Tablets)	*No*	*Yes*	*Flucloxacillin (Tablets)*	*No*	*Yes*
Ceftriaxone (Injectable)	*No*	*Yes*	*Flucloxacillin / amoxicillin (Tablets)*	*No*	*Yes*
Cefuroxime (Tablets)	*No*	*Yes*	*Gentamicin (Injectable)*	*No*	*Yes*
Chloramphenicol (Tablets)	*No*	*Yes*	*Levofloxacin (Tablets)*	*No*	*Yes*
Chloramphenicol (Injectable)	*No*	*Yes*	*Metronidazole (Tablets)*	*No*	*Yes*
Ciprofloxacin (Tablets)	*No*	*Yes*	*Metronidazole (Suspension)*	*No*	*Yes*
Clarithromycin (Tablets)	*No*	*Yes*	*Norfloxacin / metronidazole (Tablets)*	*No*	*Yes*
Amoxicillin / clavulanic acid (Tablets)	*No*	*Yes*	*Ofloxacin / ornidazole (Tablets)*	*No*	*Yes*
Clindamycin (Tablets)	*No*	*Yes*	*Phenoxymethylpenicillin (Tablets)*	*No*	*Yes*
Clindamycin (Injectable)	*No*	*Yes*	*Tetracycline (Tablets)*	*No*	*Yes*

Enumerator: Remove the unused antibiotics and put the used antibiotics in front of the respondent.

101 *Which of the antibiotics in front of you have you to your child in the **last 3 months**? Please pick the ones you have given to your child in the **last 3 months** and put them into one pile.*

Antibiotic (Formulation)	*Recognise*		*Antibiotic (Formulation)*	*Recognise*	
Amoxicillin (Tablets)	*No*	*Yes*	*Cloxacillin (Tablets)*	*No*	*Yes*
Amoxicillin (Suspension)	*No*	*Yes*	*Cloxacillin (Suspension)*	*No*	*Yes*
Ampicillin (Tablets)	*No*	*Yes*	*Cotrimoxazole (Tablets)*	*No*	*Yes*
Azithromycin (Tablets)	*No*	*Yes*	*Cotrimoxazole (Suspension)*	*No*	*Yes*
Benzathene Penicillin (Injectable)	*No*	*Yes*	*Doxycycline (Tablets)*	*No*	*Yes*
Benzylpenicillin (Injectable)	*No*	*Yes*	*Erythromycin (Tablets)*	*No*	*Yes*
Cefalexin (Tablets)	*No*	*Yes*	*Erythromycin (Suspension)*	*No*	*Yes*
Cefixime (Tablets)	*No*	*Yes*	*Flucloxacillin (Tablets)*	*No*	*Yes*
Ceftriaxone (Injectable)	*No*	*Yes*	*Flucloxacillin / amoxicillin (Tablets)*	*No*	*Yes*
Cefuroxime (Tablets)	*No*	*Yes*	*Gentamicin (Injectable)*	*No*	*Yes*
Chloramphenicol (Tablets)	*No*	*Yes*	*Levofloxacin (Tablets)*	*No*	*Yes*
Chloramphenicol (Injectable)	*No*	*Yes*	*Metronidazole (Tablets)*	*No*	*Yes*
Ciprofloxacin (Tablets)	*No*	*Yes*	*Metronidazole (Suspension)*	*No*	*Yes*
Clarithromycin (Tablets)	*No*	*Yes*	*Norfloxacin / metronidazole (Tablets)*	*No*	*Yes*
Amoxicillin / clavulanic acid (Tablets)	*No*	*Yes*	*Ofloxacin / ornidazole (Tablets)*	*No*	*Yes*
Clindamycin (Tablets)	*No*	*Yes*	*Phenoxymethylpenicillin (Tablets)*	*No*	*Yes*
Clindamycin (Injectable)	*No*	*Yes*	*Tetracycline (Tablets)*	*No*	*Yes*

Enumerator: Remove the unused antibiotics and put the used antibiotics in front of the respondent.

102 *Which of the antibiotics in front of you have you to your child in the **last 14 days**? Please pick the ones you have given to your child in the **last 14 days** and put them into one pile.*

Antibiotic (Formulation)	*Recognise*		*Antibiotic (Formulation)*	*Recognise*	
Amoxicillin (Tablets)	*No*	*Yes*	*Cloxacillin (Tablets)*	*No*	*Yes*
Amoxicillin (Suspension)	*No*	*Yes*	*Cloxacillin (Suspension)*	*No*	*Yes*
Ampicillin (Tablets)	*No*	*Yes*	*Cotrimoxazole (Tablets)*	*No*	*Yes*
Azithromycin (Tablets)	*No*	*Yes*	*Cotrimoxazole (Suspension)*	*No*	*Yes*
Benzathene Penicillin (Injectable)	*No*	*Yes*	*Doxycycline (Tablets)*	*No*	*Yes*
Benzylpenicillin (Injectable)	*No*	*Yes*	*Erythromycin (Tablets)*	*No*	*Yes*
Cefalexin (Tablets)	*No*	*Yes*	*Erythromycin (Suspension)*	*No*	*Yes*
Cefixime (Tablets)	*No*	*Yes*	*Flucloxacillin (Tablets)*	*No*	*Yes*
Ceftriaxone (Injectable)	*No*	*Yes*	*Flucloxacillin / amoxicillin (Tablets)*	*No*	*Yes*

For peer review only - http://bmjopen.bmj.com/site/about/guidelines.xhtml

Cefuroxime (Tablets)	*No*	*Yes*	*Gentamicin (Injectable)*	*No*	*Yes*
Chloramphenicol (Tablets)	*No*	*Yes*	*Levofloxacin (Tablets)*	*No*	*Yes*
Chloramphenicol (Injectable)	*No*	*Yes*	*Metronidazole (Tablets)*	*No*	*Yes*
Ciprofloxacin (Tablets)	*No*	*Yes*	*Metronidazole (Suspension)*	*No*	*Yes*
Clarithromycin (Tablets)	*No*	*Yes*	*Norfloxacin / metronidazole (Tablets)*	*No*	*Yes*
Amoxicillin / clavulanic acid (Tablets)	*No*	*Yes*	*Ofloxacin / ornidazole (Tablets)*	*No*	*Yes*
Clindamycin (Tablets)	*No*	*Yes*	*Phenoxymethylpenicillin (Tablets)*	*No*	*Yes*
Clindamycin (Injectable)	*No*	*Yes*	*Tetracycline (Tablets)*	*No*	*Yes*
*If any antibiotics are in the given in the last 14 days pile, the child is **ineligible** for study participation.*					

Nasal Sample Collection:

103	*NP swab collected?*	***No***	***Yes***
104	*If no swab was collected, specify why not.*		
105	*Was the sample you collected 'adequate'?*<br>*(Adequate: swab passed to the back of nasopharynx for at least 3 seconds and twisted 360°)*	***No***	***Yes***
106	*Is there nasal mucus on swab?*	***No***	***Yes***

107	*Scan/enter the Lab barcode*	

Rectal Sample Collection:

108	*Rectal swab collected?*	***No***	***Yes***
109	*If no swab was collected, specify why not.*		
110	*Was the sample you collected 'adequate'?*<br>*(Adequate: swab passed to the rectum for at least 3 seconds and twisted 360°)*	***No***	***Yes***
111	*Is there faeces on swab?*	***No***	***Yes***

112	*Scan/enter the Lab barcode*	

113	*Form completed by (Enumerator Code):*	*Code*	|___|___|___|
114	*Form completed by:*	*Signature*	

For peer review only - http://bmjopen.bmj.com/site/about/guidelines.xhtml
